# Patients’ responses to incidentally discovered silent brain infarcts – a qualitative study

**DOI:** 10.1186/s41687-019-0112-7

**Published:** 2019-04-15

**Authors:** Lester Y. Leung, Paul K. J. Han, Christine Lundquist, Gene Weinstein, David E. Thaler, David M. Kent

**Affiliations:** 1 Department of Neurology, Tufts Medical Center, 800 Washington Street, Box 314, Boston, MA 02111 USA; 2 Center for Outcomes Research & Evaluation, Maine Medical Center Research Institute, Portland, USA; 3 Predictive Analytics and Comparative Effectiveness Center, Tufts Medical Center, Boston, USA; 4 Department of Radiology, Massachusetts General Hospital, Boston, USA

**Keywords:** Incidental, Counseling, Uncertainty, Stroke, Silent stroke, Silent brain infarction, Silent brain infarcts, Patient centered outcomes research, Patient preferences

## Abstract

**Background:**

Incidentally discovered silent brain infarcts (id-SBIs) are an understudied condition with probable clinical significance, but it is not known how patients respond to or prioritize this condition. We sought to assess reporting of id-SBIs and how patients approach their diagnosis.

**Methods:**

Patients with id-SBIs were identified from sequential scans between 12/2015–5/2016, were referred by treating clinicians, or self-referred for the study. This study used qualitative semi-structured interviews. Purposeful sampling was used to achieve diversity in acuity, setting, and recruitment strategy. Interviews were audio-recorded and transcribed. A constant comparative method was used to develop a coding schema, find consensus, and iteratively explore emergent themes until thematic saturation was achieved.

**Results:**

Only 10 of 102 patients prospectively identified by neuroimaging were informed of the imaging findings. Twelve participants in total were interviewed. Among the study participants, the primary themes were cognitive, emotional, and behavioral responses to diagnostic, prognostic, and therapeutic uncertainty regarding id-SBIs. Clinicians described id-SBIs to participants as an ambiguous condition. Participants feared potential consequences of id-SBIs, including symptomatic stroke, dementia, and disability. Participants attempted to reduce uncertainty with strategies including equating id-SBIs with symptomatic stroke, self-education about stroke, and seeking second opinions.

**Conclusion:**

Participants considered id-SBIs to be a serious medical condition. Ambiguous counseling by clinicians on id-SBIs provoked or failed to attenuate fear, leading to participants adopting strategies aimed at reducing uncertainty.

**Electronic supplementary material:**

The online version of this article (10.1186/s41687-019-0112-7) contains supplementary material, which is available to authorized users.

## Background

In clinical care, diagnostic uncertainty often complicates medical decision making, as well as the counseling of patients by clinicians [1, 2]. Silent brain infarcts (SBIs) are a prime example with important health implications. SBIs affect approximately 20% of adults over age 50 and have consequences including symptomatic stroke and dementia in neuroimaging-screened cohorts [3–6]. However, in the absence of standardized screening, SBIs are by definition discovered incidentally in routine clinical care during the evaluation of other conditions. Considering that SBIs are asymptomatic and lack an overt, immediate functional impact for patients, clinicians may be uncertain about the diagnostic significance of SBIs, potentially leading to inconsistent reporting to patients and counseling of patients with this condition. [7]

Adding to this uncertainty is that patients with incidentally discovered SBIs (id-SBIs) are a completely unstudied population facing an emerging clinical decision making scenario: prior studies of natural history and outcomes have only assessed patients with SBIs undergoing protocol-driven neuroimaging. The American Heart Association/American Stroke Association (AHA/ASA) has recommended preliminary management strategies and called for further research to address multiple knowledge gaps, specifically highlighting studying the reporting of radiologic findings and improving understanding of id-SBIs and their potential differences from SBIs in screened cohorts [8]. Previously, we interviewed a diverse group of clinicians from various specialties who had experience providing care for patients with id-SBIs, and we found that they endorsed considerable diagnostic and prognostic uncertainty that they viewed as barriers to addressing this condition [7]. In this study, we explored the perspectives of patients on these issues, focusing on how patients with id-SBIs interpret and respond to the diagnosis of id-SBIs and how they manage uncertainties surrounding this diagnosis.

## Methods

### Study design, participants, and data collection

This study employed individual semi-structured qualitative interviews of patients with id-SBIs to explore their concerns, priorities, and approaches to addressing this condition, to identify new concepts and hypotheses, and to build new theoretical understandings. Participants were recruited by telephone or face-to-face from inpatient and outpatient practices at a tertiary care medical center (Tufts Medical Center, Boston, MA). Participants were adults ages 18 and over; had no prior history of stroke, transient ischemic attack (TIA), or dementia; had to consent and participate in the interview in English; had to be aware of the neuroimaging findings prior to the interview; and had to have received the diagnosis from a clinician other than the interviewer (LYL). Participants were identified and recruited through at least one of three strategies: (1) identification through radiologic reporting of brain infarction with verification of clinical silence, (2) identification by a treating clinician, or (3) self-identification. A vascular neurologist (LYL) reviewed neuroimaging directly to confirm infarction (based on consensus definitions) and verified clinical silence through review of clinical documentation or discussion with treating clinicians [4, 9]. Purposeful sampling was used to achieve diversity in sex, race, acuity of SBIs (acute, chronic), clinical setting, and recruitment strategy. Participant characteristics were collected from the electronic medical record including demographics, medical history, and SBI features. One-on-one interviews were conducted by LYL (male attending vascular neurologist with clinical equipoise regarding SBIs) in the hospital room or neurology clinic and lasted 30–60 min. Interviews were audio-recorded and transcribed verbatim by a professional transcription service.

### Interview content

The interview guide included both open-ended questions and closed-ended probes to explore patients’ perceptions and interpretations of their diagnosis, advice received from their clinicians, and their responses to their diagnosis (Additional file 1). The guide was developed by three investigators (LYL, PKJH, CL) and was pilot tested with two patients from which the initial codebook was developed. Single interviews were conducted in phases (groups of four) with iterative revisions to the interview guide between phases to explore unanticipated themes. Interviews were repeated until thematic saturation was achieved.

### Data analysis

Two investigators (LYL, CL) performed line-by-line, software-assisted coding of anonymised interview transcripts (Additional file 2) using NVivo (V.11; QSR International, Melbourne, Australia). Three investigators (LYL, PKJH, CL) developed an initial codebook through independent review and team-based reconciliation of the first two coded transcripts, using an open-ended, inductive, “grounded theory” approach in which the investigators strove to minimize preconceptions, allowing important themes to emerge and categorizing thematic content in a hierarchical logically coherent conceptual schema [10, 11]. Through an iterative “constant comparative” method, emergent themes were incorporated into the codebook following each phase of interviews, independent coding, and coding reconciliation [12]. The investigators met at the end of each phase to discuss coding and recruitment decisions, resolve disagreements, and revise the interview guide and codebook.

## Results

**Fig. 1:**
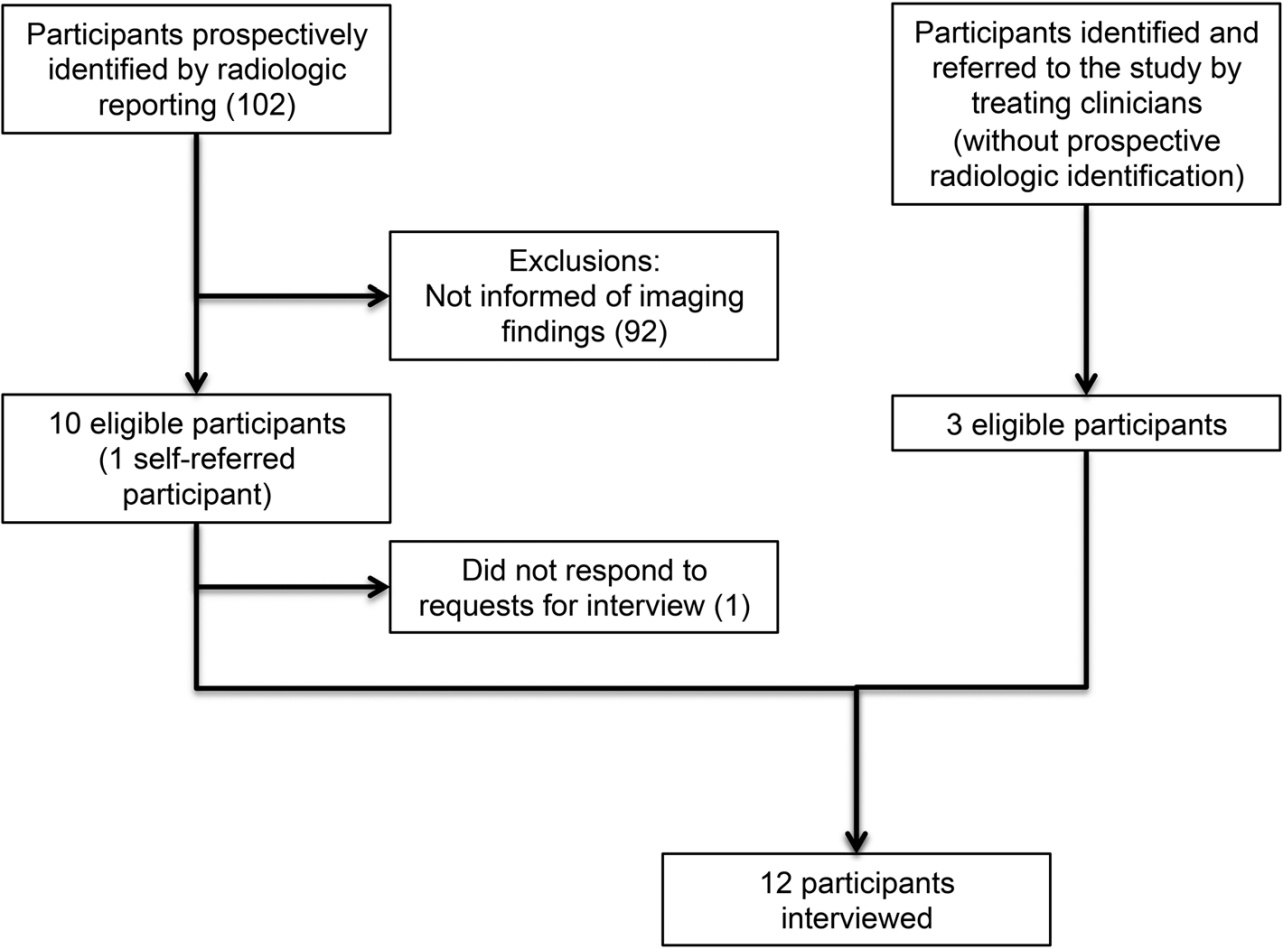
Recruitment of participants

**Fig. 2:**
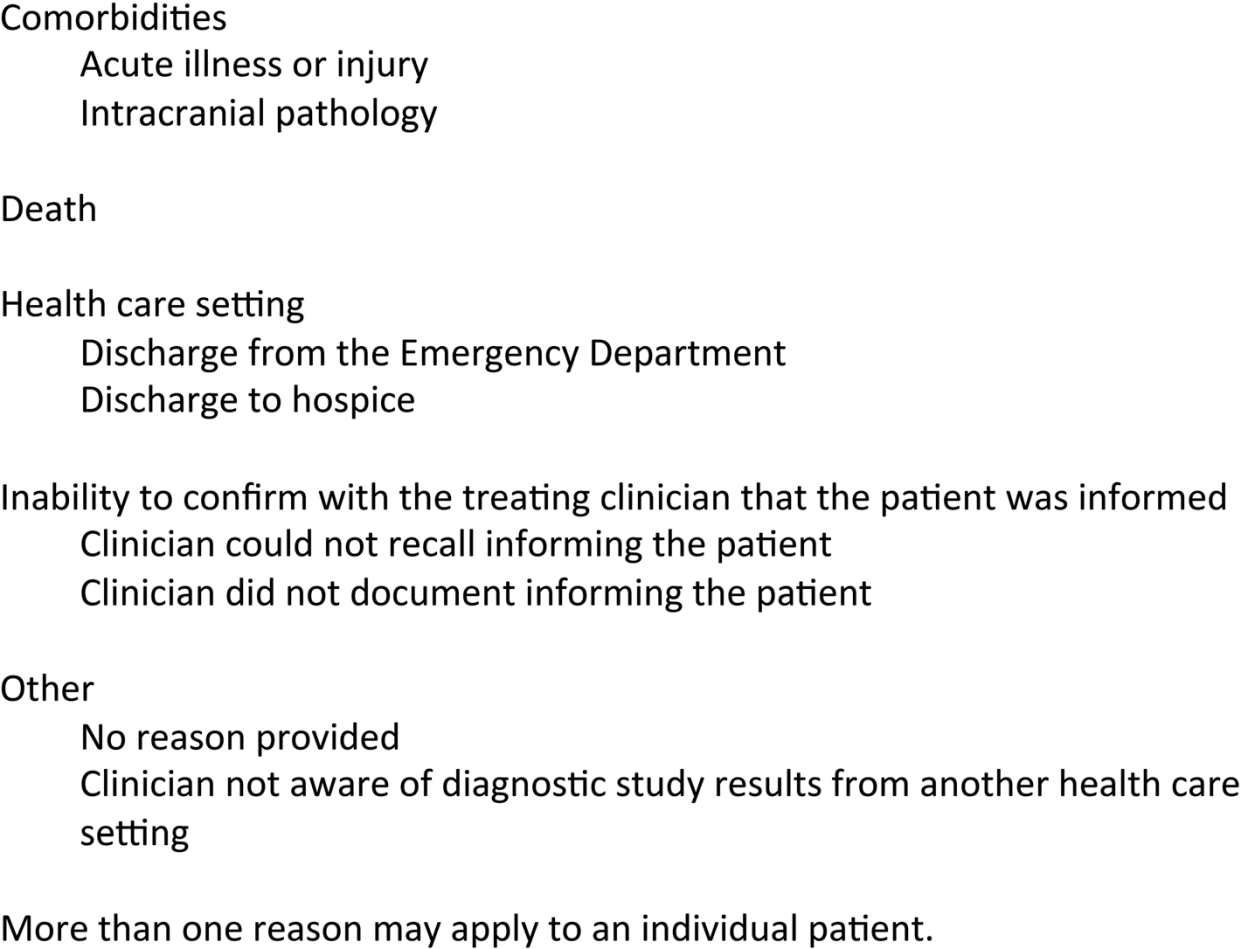
Reasons for lack of reporting of SBIs to patients

Recruitment proceeded simultaneously with all three aforementioned strategies, often used in combination (Fig. 1). Between 12/2015–5/2016, 102 patients were prospectively identified as having SBIs by neuroradiologist report and clinical verification. No patients had more than one study identifying SBIs during this time period. Ninety-two patients with SBIs (90%) were not informed of the imaging findings and were thus ineligible for this study. One of the remaining 10 informed patients did not respond to requests for interview. Three additional patients were referred by treating clinicians with brain MRIs performed at other facilities or outside the December–May prospective identification time window. One of these three referred patients learned of her infarcts by reading a radiology report, after which she contacted her treating clinician. In total, 12 participants completed interviews. Participant characteristics are shown in Table 1. Reasons for lack of reporting of imaging findings to patients are described in Fig. 2. Interview transcript analysis revealed three categories of responses to the detection of id-SBIs: cognitive, emotional, and behavioral responses (Table 2). Constant comparison of these categories with data from subsequent groups led to the identification of several specific themes which are presented below. Participants’ perspectives were not noticeably influenced by variables used for purposeful selection.

**Table 1:** Characteristics of patient participants

Characteristic	Subcategory	Median (IQR) or n
Age (years)		61 (54–72)
Sex	Men	3
	Women	9
Race	Black	2
	White	10
Acuity of SBIs on neuroimaging	Acute/subacute	4
	Chronic	8
Location of SBIs	Only deep/subcortical	8
	Only cortical/juxtacortical	2
	Both	2
Presence of white matter disease	Yes	10
	No	2
Clinical setting	Inpatient	4
	Outpatient	8
Type of clinician delivering the diagnosis	Internist	1
	Neurologist	7
	Other	4^a^
Comorbidities^b^	Atrial fibrillation	1
	Coronary artery disease	2
	Concurrent tobacco use	1
	Diabetes	4
	Hypercholesterolemia	6
	Hypertension	7
	Migraine	6
	Obesity	2
	Obstructive sleep apnea	1

^a^Includes two SBIs reported by Neurosurgeons, one by a Cardiologist, and one where the patient read the imaging report directly

^b^More than one may be present

**Table 2:** Participants’ responses to the incidental discovery of SBIs

Categories	Subcategories	Representative quotations
Emotional	Fear	“I was in a panic, in a big panic.” – P2 “I’m afraid I’m going to have dementia or a major stroke.” – P2 “These were minor strokes that I didn’t even feel … any time I get a headache now I’m concerned.” – P2
	Surprise	“I was caught unawares when the word ‘stroke’ came up.” – P10 “Nothing was mentioned about a stroke to me originally …” – P4 “My primary doctor: she was surprised. She looked so sorry for what’s happened … my body, my health was good.” – P8
	Personal responsibility	“There’s damage that’s already been done. I could eat healthy for the rest of my life, but it won’t necessarily undo that damage.” – P3
Cognitive	Equating SBIs and strokes	“I just want to know what I can do to make myself not have another stroke.” – P11 “What is the difference between a stroke with symptoms and a stroke without?” – P11
	Wake-up call	“It was a wake-up call. No one is promised tomorrow.” – P12 “There’s a lot I’m going to change.” – P1 “As long as it’s not a drug.” – P1
	Discounting	“I don’t want this to be the 800 pound gorilla on my back for the rest of my life … I’m not going to have it be like I have a stroke.” – P10
Behavioral	Information seeking	“Well, I know very little except I had an MRI of my brain.” – P9 “I really don’t know what I can do. Is there a stroke diet? If you exercise or if you change behavior, will that reduce the chance of having another one?” – P10 “I’ll ask more questions regarding this and gather more information.” – P5
	Seeking second opinions	“I’m weighing it all out now with everybody’s thoughts … Sometimes there’s different opinions. I just got to take them all out and see, put them all together and decide what to do.” – P4
	Seeking social support	“I don’t hide my sickness from nobody. Not my friends, not my children. I call them up and I tell them … I call my son … His son calls his aunt, his nephew, his sister, they call everybody … I call my friends, I tell them … My daughter called him, and she called my two sons in Barbados, and they called me … It’s not right to hide sickness from no one.” – P7

*P#* Participant number

### Cognitive responses

With limited guidance from clinicians on how to think about id-SBIs, participants adopted cognitive strategies to manage the uncertainties around their diagnosis. These responses were closely intertwined with emotional responses and framed behavioral responses.

#### Equating SBIs and strokes

When participants were given the diagnosis of “stroke,” their clinicians used modifiers such as “silent,” “small,” or “old,” with one notable exception. Nonetheless, participants did not perceive id-SBIs and symptomatic strokes as distinct entities. Participants defaulted to referring to their id-SBIs as “strokes,” worried about future consequences including “another stroke,” and expressed views indicating that they considered id-SBIs to be as grave as symptomatic stroke.

*“I don’t want to go back to not thinking about what I eat, because I’m afraid I’ll have another stroke. This time I know I’ll end up in the hospital or even dead.” – Participant 11*

The exceptional participant reported that the clinician did not seem to differentiate between id-SBI and symptomatic stroke:

*“I was under shock when he said to me ‘you have stroke.’ My daughter ask doctor, ‘Is that because not big symptoms? Is that maybe mini stroke?’ And he said, ‘Stroke is stroke. Doesn’t matter big stroke or little stroke.’” – Participant 8*

#### Wake-up call

A third of the participants specifically described thinking of the detection of id-SBIs as a “wake-up call” to inspire behavioral changes to reduce risk of future adverse health consequences. These participants expressed a high level of motivation to improve their health.

*“This happened, and it was a wake-up call. It’s never going to happen again, God willing, because I’m going to do everything in my power to make sure.” – Participant 3*

However, by contrast, participants did not find specific instructions conveyed by their clinicians to be memorable or impactful. Four were advised to start or change an antiplatelet medication, six were advised to see a specialist, and one was advised to stop smoking cigarettes; there were no observed patterns of participant and SBI characteristics (in Table 1) related to whom clinicians gave advice. Perceiving minimal clinician guidance, participants expressed preferences to implement lifestyle changes rather than medical therapies to reduce their health risk.

*“I would prefer to modify diet and exercise without taking another medication.” – Participant 10*

Implicit in this cognitive response was a perception that the risk of future stroke was in fact reducible, as well as a perception of confidence in one’s ability to enact risk-reducing changes.

#### Discounting

Almost all participants expressed conflicting perceptions regarding the degree of concern warranted by their id-SBIs: some were initially very worried and then were reassured, whereas others were initially placated but later became progressively more anxious about their health. One participant described a constant struggle to find the appropriate level of concern and attention to give to the id-SBIs, ultimately leading to him discounting their importance:

*“I’m not a hypochondriac, but this concerned me … I never gave it much thought because I didn’t want to dote on the fact that it could be something wrong … My thought process with the stroke is I have enough other problems … even though it could be life threatening, my quality of life is fine, it hasn’t altered how I see anything.” – Participant 10*

When asked about how he prioritizes his health issues, he responded:

*“This is number one. I don’t take it lightly, but I can’t let it live for me. Once I’m made aware of something that is bad for me, I do whatever I can to avoid it … I really don’t want to make this the center of how I’m going to live the rest of my life … Right now, there are other things that physically I have. I’m in pain. That registers with me that something’s got to be done. But the stroke thing? I mean, should it be number one on my priority list? Probably should, but right now it isn’t.” – Participant 10*

### Emotional responses

Participants described strong emotional responses upon learning of the id-SBIs. These emotional responses were triggered by clinicians mentioning “stroke” when delivering information on imaging findings, drawing upon the cognitive strategy of equating id-SBIs and stroke.

#### Fear

Among participants in this study, fear was the predominant emotional response. With one exception, participants informed of id-SBIs by a clinician were given a diagnosis of “stroke”: these participants uniformly endorsed fear associated with this diagnosis. Two participants reported feeling “panic” and “despair” in response to the diagnosis.

*“Being told I had a ‘slim stroke,’ scared the life out of me. I didn’t know what to expect.” – Participant 1*

*“I honestly don’t remember part of the conversation after that because I was so stuck on the word ‘silent stroke.’” – Participant 3*

By contrast, the participant who received a different diagnosis expressed that she was told she had a “TIA”: she did not endorse initial fear or concern.

*“I hear there are many people who have many TIAs and get through life okay. They don’t all necessarily end in having a major stroke. So I’m not really worried about it.” – Participant 9*

Despite variable or ambiguous counseling provided by clinicians regarding the potential impact of id-SBIs on their health, all participants expressed concerns about future consequences (including the participant who was told she had a TIA). Specifically, participants independently identified symptomatic stroke, dementia, and loss of independence as potential outcomes of id-SBIs provoking fear.

*“I’m not afraid of dying. I’m more afraid of living and having to depend on somebody to take care of me because I’ve had a stroke.” – Participant 2*

Half of the participants traced their fear to uncertainty about whether they would experience a future asymptomatic or symptomatic stroke.

*“It is scary not knowing when you’re going to have one, if I’m going to have another one again … if I do have another one, is that one going to show physical signs next time?” – Participant 12*

#### Surprise

All participants were “surprised” or “shocked” when they learned about the id-SBIs. This emotional response was often connected to an expectation that an important medical condition should have overt symptoms.

*“I was under shock how I had a stroke, because I didn’t feel nothing special.” – Participant 8*

A surprised response was also tied to delayed or absent reporting of the imaging findings.

*“She said it showed I have had a previous stroke. It was a big surprise, I had no idea at all. No one has ever mentioned that to me” – Participant 6*

This response also triggered reflections on the prior health of the participants. In some cases, id-SBIs were unexpected due to a perception of good health prior to their detection.

*“I’ve lived a pretty healthy life. That’s why I’m really surprised this is all happening.” – Participant 2*

This emotional response was sometimes observed by participants in their clinicians, and the participants may have taken cues from their clinicians to express this feeling.

*“She was somewhat taken aback. She had been my primary care for a while, and I took by her demeanor there was a concern there. It was something that maybe she was not expecting.” – Participant 10*

#### Feeling of personal responsibility

While the detection of id-SBIs provoked surprise in some participants based on prior perceptions of good health, the process of reflecting on prior health and behaviors triggered feelings of personal responsibility for health status in some participants.

*“I would like to know if I did something in my past that may have caused this.” – Participant 2*

These participants suggested associations between risk factors for stroke (e.g. hypertension, low levels of physical activity, dietary patterns) and id-SBIs. They cited the need for behavioral change, assuming that they had “not done enough” in the past to preserve their health.

*“I’m just trying to do better: be the healthiest I can be with exercise and diet and that kind of stuff.” – Participant 4*

### Behavioral responses

Participants described changing their behaviors in response to the id-SBIs. These behavioral responses were self-initiated, self-directed (independent of clinician guidance), and influenced by their emotional and cognitive responses.

#### Information seeking

Participants reported being given very little information regarding their diagnoses. One participant described her experience learning about her id-SBIs:

*“I know nothing about my diagnosis. I didn’t know I had the strokes until they let me know that I had two stroke spots on my brain … Even though they just came out and told me, they didn’t ask me any questions or anything. I would have liked to have known some things. You get nervous when you hear things like that. And they don’t ask questions. They don’t give me any medicine. So you’re like, ‘It sounds serious, I had two strokes. Are you going to give me any medicine to start me doing something?’ That worried me.” – Participant 11*

Following detection of id-SBIs, a third of participants described active efforts to improve their knowledge. Instead of learning about SBIs specifically (and their possible differences with symptomatic strokes), participants described seeking actionable information on how to prevent recurrent stroke. This information was primarily acquired by asking questions to clinicians or seeking written material elsewhere.

#### Seeking second opinions

Participants often encountered multiple clinicians following detection of id-SBIs, and they often received variable and sometimes conflicting interpretations of imaging findings and their relevance.

*“It caused a lot of confusion because one doctor said there was nothing, another looking at the scan was concerned, and then after speaking to the doctor at Tufts … it’s just very confusing.” – Participant 2*

In most cases, primary care physicians or internists referred participants to specialists for further guidance. However, in two cases, the participants independently sought additional medical opinions. The first participant learned about her id-SBIs by reading the report of her brain MRI ordered by a neurosurgeon and obtained as a screening study in the context of a family history of aneurysms. The neurosurgeon recommended seeing a vascular neurologist, but when the neurologist and her primary care physician offered differing opinions, the participant decided to seek counsel from a cardiologist as well:

*“I have differing opinions from my primary and neurologist... Which way to lean? Not sure, but at this point, I was leaning towards not taking the medication because I’d rather not... I guess the cardiologist will hopefully break the tie here. It’s hard and obviously everyone’s going to have different opinions. Being a patient, you’re like, ‘Well, who do I listen to?’” – Participant 4*

In the second scenario, the participant went to an Emergency Department for an evaluation of a left arm sensory disturbance. She underwent a brain MRI that did not show an acute infarct, but it did show a left cerebral hemisphere brain lesion that could represent an unrelated id-SBI. She was diagnosed with a peripheral nerve compression. Nonetheless, she actively sought a subspecialist evaluation in the neurology clinic, concerned that she may have had a stroke, despite reassurance from the clinicians in the Emergency Department.

#### Seeking social support

Three participants described feeling it was important to share their diagnosis with their support networks, usually family members or friends, as a means of obtaining emotional support.

*“When I stay at home … I think more and more... and worry. Every time worry. But now I started work, that’s better for me, because I talk with my friend there, we try make joke about it, because they see that I’m depression and I scared...” – Participant 8*

One participant highlighted the gravity of the detection of her id-SBI and the importance of personally sharing this burden with a key supportive figure in her life, her father:

*“My husband actually told most of my family and my friends … So I didn’t say much to people … I told my father … He was upset. I could tell from his voice he wanted to break down, but he didn’t. He was the strong person … He stayed on the phone with me, let me let everything out, talk with me, consoled me, and just told me everything was going to be okay. If anything, he would be here … I’ve always talked with him and told him after every appointment.” – Participant 12*

## Discussion and conclusion

### Discussion

**Fig. 3:**
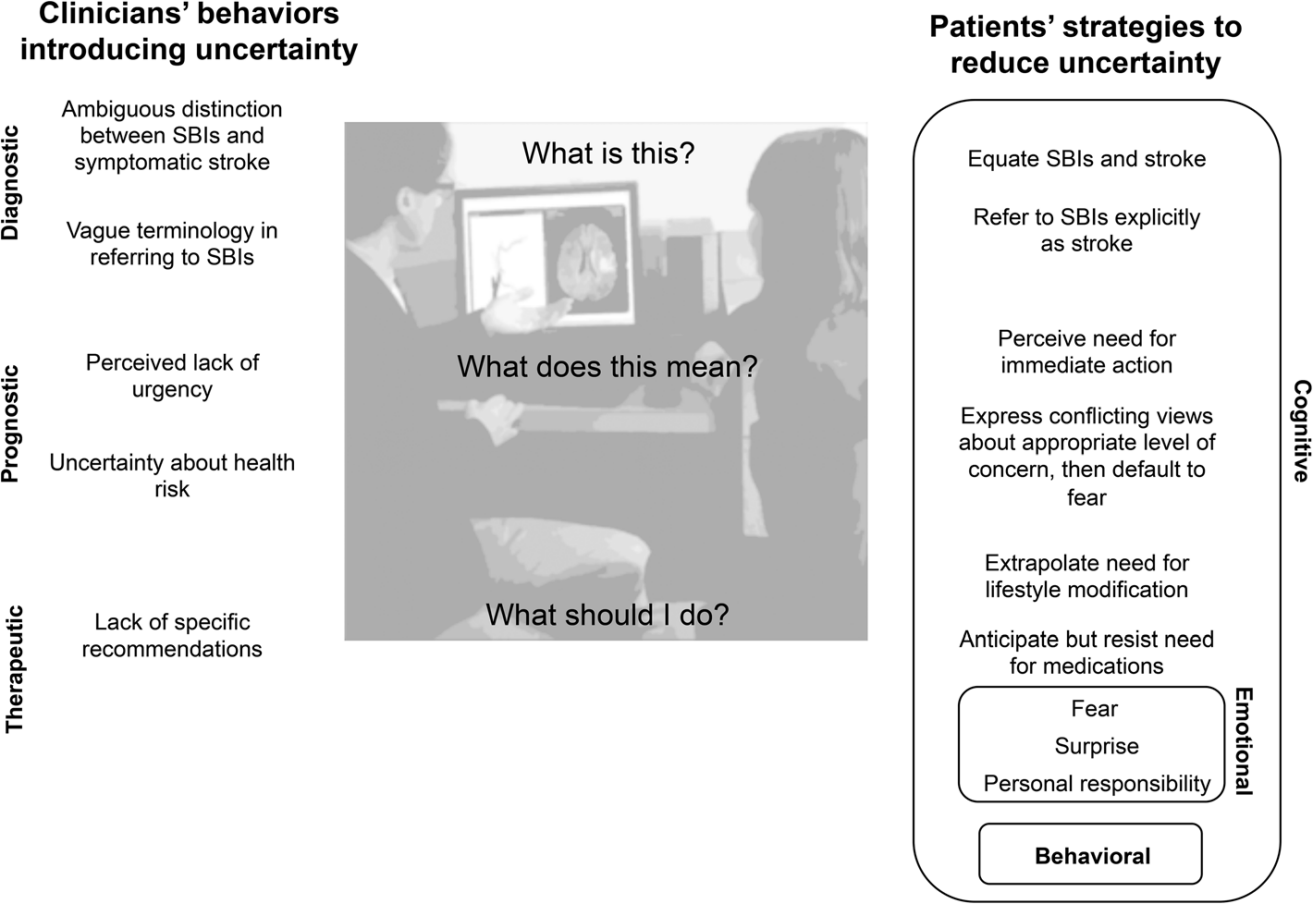
Uncertainties introduced by clinician behavior and participants’ strategies to reduce uncertainty

To our knowledge, this is the first study to assess the perspectives of patients with id-SBIs: this study yields important insights on their responses to the diagnosis and its associated uncertainties. This study reveals that these participants took the diagnosis of id-SBIs seriously and encountered difficulty navigating the uncertainties around this diagnosis, highlighting the need for clinicians and policymakers to develop a more structured, patient-oriented approach to this emerging condition. Participants’ responses (Fig. 3) were consistent with a recently developed conceptual model of “uncertainty tolerance,” a set of psychological responses—cognitive, emotional, and behavioral—to the conscious awareness of ignorance about particular aspects of the world [13].

Participants notably reported strong emotional responses—fear, surprise, and a feeling of personal responsibility—to this ambiguous diagnosis. These responses co-occurred with cognitive responses equating id-SBIs and symptomatic stroke and a “wake-up call” to take urgent action, as well as negative appraisals for their current health and future health risks. This mixture of cognitive and emotional responses set the stage for a critical discussion of risk reduction strategies with their clinicians and a heightened need for certainty about how to manage their condition.

However, patients often felt that their clinicians failed to meet this need: they perceived their clinicians’ statements on the significance, prognosis, and management of id-SBIs as vague and uncertain. These perceptions correspond with the uncertainties expressed by clinicians in a parallel study of clinicians’ perspectives on id-SBIs [7]. In response to this persisting uncertainty, participants demonstrated “ambiguity aversion”—a phenomenon characterized by pessimistic appraisals of risk and a tendency to “devote excessive attention to the worst case scenarios” when risks are unknown, or “ambiguous”—i.e., lacking in reliability, credibility, or adequacy [14, 15]. Notably, perceived or real ambiguity in clinicians’ statements on id-SBIs may have been scientifically justifiable, given the current state of available knowledge [7, 8]. To participants, however, such ambiguity was psychologically intolerable; they desired unambiguous and immediate answers, perceiving id-SBIs as a medical condition needing urgent attention. Despite its objectively non-urgent nature, the diagnosis created what Kruglanski has called a high situational “need for closure”—the “desire for a firm answer to a question and an aversion toward ambiguity” [16].

Where responses diverged from ambiguity aversion, however, was in the behavioral domain. Instead of avoidance or deferral of decision making or action—the usual behavioral response to ambiguous risk information—most participants were predisposed towards taking action to reduce their risk of symptomatic stroke. In other words, they adopted a precautionary perspective—treating ambiguity in their diagnosis as warranting action rather than justifying inaction. This tendency was not universal: one participant discounted the risk of stroke (leading to inaction), whereas most participants responded to ambiguity by seeking either further information or second opinions.

Participants’ behavioral responses highlight a missed opportunity for continued conversations between individual clinicians and patients with id-SBIs. When approaching a condition with considerable uncertainty, clinicians may be able to help patients by explicitly identifying areas of uncertainty, guiding them with currently available knowledge, and inviting them to participate in an ongoing conversation with subsequent counseling incorporating new research. For these participants, the clinicians were not able to effectively attenuate their fear or aid them in tolerating uncertainty. The need to manage uncertainty is particularly great given the emotional significance of the term “stroke” (even with “silent” as a modifier), which provokes great fear among many patients. Without sufficient direction from their clinicians, participants sometimes sought alternative sources of information, as well as pursuing non-informational means of managing uncertainty, including seeking social support or attempting to improve their health without clinician guidance.

Although participants were often uncertain about the best approach to reduce their health risks, most were motivated to improve their health. Furthermore, despite clinicians’ uncertainty about the significance of id-SBIs, our participants were clear about their priorities: they were interested in reducing their risk of symptomatic stroke, dementia, and loss of independence (i.e. known outcomes from studies in neuroimaging-screened cohorts). These patient-oriented outcomes should be included in future treatment studies for patients with id-SBIs.

This study has several strengths. First, as this population of patients has not been previously studied, this study is the first to highlight empirically that the rate of id-SBI reporting is low (approximately 10%). Accordingly, when reassessed in larger cohorts, individuals with id-SBIs will have to be identified through novel means (i.e. not through clinician reporting, documentation, or diagnostic coding). Second, this study is valuable in its timing: id-SBIs are a condition for which there is no consensus regarding optimal strategies for counseling, detection, and management. [7] While the AHA-ASA has suggested an approach to SBIs for clinicians, it is uncertain if clinicians will uniformly follow those recommendations for all patients with SBIs [8]. This may arise in part as a result of heterogeneity of clinical scenarios in which SBIs are incidentally discovered, but this has not yet been studied. [7] As a result, patients with id-SBIs will likely continue to encounter ambiguous or conflicting recommendations from their clinicians without further research on id-SBIs. Third, this study’s participants were recruited through strategies aimed at increasing diversity: there were differences in the acuity of the imaging findings (possibly influencing clinician certainty in the diagnosis and recommendations), clinical setting, clinician specialty, and method of identification. Accordingly, these participants likely encountered clinicians who provided a wide spectrum of perspectives on SBIs.

This study also has important limitations. First, because a minority of patients identified through radiologic reporting were informed, the sample may represent a select group for whom id-SBIs were interpreted by reporting clinicians as being significant. For other patients with competing risks, clinicians might consider id-SBIs to be a low clinical priority, and thus not disclose the id-SBIs. As such, our participants may represent a select sample of patients who have been pre-identified as possible candidates for stroke prevention. Second, our sample was small; however, the course of findings that emerged from our interviews led us to believe that we achieved thematic saturation for our topic of interest, and prior qualitative studies have indicated that even modest numbers of interviews are reasonable for providing adequate coverage of relevant themes [17, 18]. Third, this study was performed with limited resources; we were not able to implement additional methods to ensure validity such as member checking. Finally, this was a single center pilot study which was not designed to be truly generalizable; however, it is valuable in providing the first assessment of the patient perspective on this important problem, providing a foundation for future multicenter studies.

### Conclusion

Incidentally discovered SBIs are an important but ambiguous condition. This study highlights patients’ responses to the uncertainties expressed by their clinicians surrounding the diagnosis of id-SBIs, and the implications of these responses on the potential for decay of the therapeutic relationship between clinicians and patients with this condition. This study provides evidence of the importance of id-SBIs to patients informed about their neuroimaging findings and their need for improved guidance by clinicians. Until future studies establish appropriate diagnostic and therapeutic management of id-SBIs, further studies are needed to guide improvements in patient counseling and education by clinicians to help patients tolerate the uncertainties related to this diagnosis.

## Additional files

Additional file 1: Interview guide (final). This is the final version of the iteratively developed interview guide. (DOC 56 kb)

Additional file 2: Interview transcripts. These are the original, anonymised interview transcripts for all participants. (DOC 445 kb)

## Abbreviations

AHA-ASA: American Heart Association-American Stroke Association
Id-SBIs: Incidentally discovered silent brain infarcts
MRI: Magnetic resonance imaging
SBIs: Silent brain infarcts
TIA: Transient ischemic attack
