# Supplementary material for: Patients’ responses to incidentally discovered silent brain infarcts – a qualitative study
Source: J Patient Rep Outcomes. 2019 Apr 15;3:23. doi: 10.1186/s41687-019-0112-7 (PMC6462438; doi:10.1186/s41687-019-0112-7)
Supplement: Supplementary file 1 — Interview guide (final). This is the final version of the iteratively developed interview guide. (DOC 56 kb) [file 41687_2019_112_MOESM1_ESM.doc]

**Additional File 1 - Interview Guide (final version).**

**Exploratory Questions (required)**

E1. Tell me what you know about your diagnosis. (Knowledge)

E2. Who gave this diagnosis to you, and what were the circumstances? (Information Transfer)

1. What type of doctor gave you the diagnosis?
2. How did he or she present the diagnosis to you? (Information transfer)
3. Were the terms “incidental” or “unexpected” used in describing this stroke?
4. What does it mean to you that this was “incidental” or “unexpected”?

E3. What are the symptoms that first brought you to your doctor? (Relation)

a) Do you think your original symptoms are connected to this diagnosis?

E4. What advice were you given by your doctor? (Information Transfer, Certainty/Uncertainty)

1. How certain did the doctor seem about the advice he or she gave you?
2. Is there anything that he or she emphasized?
3. How concerned did the doctor seem to be?
4. Did he or she express any doubt? If so, what did he or she express doubt about?
5. In general, how well do you think doctors understand this diagnosis?

E5. How did learning about this diagnosis make you feel? (Emotional response, Fear)

E6. What are your concerns about having this diagnosis? (Concerns)

1. How bad do you think this is for your health? (Risk, nature)
2. Why do you think that?
3. How concerned are you about your health after learning about this diagnosis? What are you worried about?
4. Thinking about your other health issues, how does this rank in priority?

E7. So, now that you’ve been told you’ve had a silent stroke: do you think this might cause other bad things for your health? (Risk)

1. What sort of problems?
2. In your opinion, what are the chances that you will have future health problems because you had a silent stroke? (none, low chance, high chance?)
3. Why do you think that?
4. Do you think this is connected to your other health issues? If so, which ones?

E8. How did you describe this news to your family and friends? How did you describe this issue to your other doctors?

E9. How would you have wanted this finding to be explained to you?

E10. Do you think you will change anything at this time now that you have this diagnosis? (Behavioral change, Uncertainty, Urge to change)

1. Do you want to?
2. Do you think there’s any uncertainty or lack of clarity about how to deal with this?
3. How urgent is it for you to take action?
4. What kind of things do you want to do?

E11. We’re almost done with the interview. After being asked these questions, has your perception of covert strokes changed? If so, how? (Study-related)

E12. Do you have any questions or additional thoughts on this issue?

**Focused Questions (as many as possible, time permitting)**

F1. If I told you that there currently are no national guidelines and no specific research studies guiding physicians in selecting tests or therapies for silent stroke, how concerned would you be about this? (Stakeholder role in future research)

a) Would learning this fact make you more or less concerned about this condition than you were before?

b) Does knowing this affect how you perceive or trust your doctor’s advice?

c) How important is it to you that this condition be studied more?

d) If studies were to find modest (relatively small) health benefits with medications or lifestyle changes, do you think that you would be motivated by these studies to take new medications (e.g. take another daily pill) or change your lifestyle? (e.g. exercising, change diet, stopping smoking, etc.)
