# Supplementary material for: Patients’ responses to incidentally discovered silent brain infarcts – a qualitative study
Source: J Patient Rep Outcomes. 2019 Apr 15;3:23. doi: 10.1186/s41687-019-0112-7 (PMC6462438; doi:10.1186/s41687-019-0112-7)
Supplement: Supplementary file 2 — Interview transcripts. These are the original, anonymised interview transcripts for all participants. (DOC 445 kb) [file 41687_2019_112_MOESM2_ESM.doc]

**Additional File 2 – Interview Transcripts.**

**Participant 1**

**Q:** All right, so this is the first interview with our first patient. So first question for you is to tell me what you know about your diagnosis, the one that we talked about before.

**A:** Just that I Had-- I was coming down with a sick cold. And next thing you know, I fell down a flight of stairs. And then I found out a few other things had happened during the fall. But I felt all right, and I have been here in the hospital ever since.

**Q:** And so during my time seeing you, I gave you a diagnosis as well, or told you about something. Do you remember what that was?

**A:** Can't think of it right now. [00:01:04] refresh my memory, then I can go from there.

**Q:** Sure. I mentioned to you that you had a stroke. Is that right?

**A:** Yes.

**Q:** Okay.

**A:** Never had one before that I know of. But they said that the stroke that I had happened while I was here in the hospital. And it was, I guess, caused because of the fall.

**Q:** So I mentioned this to you when I saw you as a neurologist that was consulted by your medical team. Had anybody else talked to you about this before that?

**A:** No.

**Q:** Or was I was the first person to tell you the situation?

**A:** You were the first one to tell me.

**Q:** Okay. And I'm asking you some questions as if I don’t know the answer to this already, if you don’t mind.

**A:** That’s okay.

**Q:** When I told you about the diagnosis, what were the terms that I used to describe it? How did I describe it to you?

**A:** You told me what it was, and then you explained it to me of what the situation was. I got emotional because I knew people that had strokes. And I was looking at it as the worst way, because I was always told, or what I could remember, was the left side controlled the right, and the right side controlled the left. And I had aunt that was completely-- I don’t know why she’d say disabled, but it was her left side that was completely gone. She couldn’t speak, she couldn’t use her left arm or leg. But if she left her house to come with my uncle, he always knew the straight route to take to get from her house to mine. That’s the only way I can explain it. Everything else was-- she was out of her mind. I mean she was familiar with the area, where she lived. But she knew all the time where my uncle was, and all the kids would be out looking for her, between my father and my mother, her husband, all my cousins, my older brothers. But we-- and there she was, sitting out in front of our house. And thank God she was never hurt, you know. I mean she didn’t get hit by a car or stuff like that.

**Q:** So one of the distinctions between-- This is your aunt, right, who had a stroke?

**A:** Yes.

**Q:** Her stroke, you described pretty clearly that she had some symptoms and some disability related to it. And one of the ways that I described your stroke to you is that, as far as we can tell, there is no obvious symptoms that came along with it, or nothing on your examination that is really related to it, as well. One thing I wanted to ask is, you mentioned that you came to the hospital for a few things. You had a big fall when you were at home, and then came to the hospital, and you were sick with some other issues, having an infection and other things. Do you think that your original symptoms that brought you to the hospital are connected to the stroke?

**A:** No, because at the time that I came to the hospital, I didn’t find out until days later that I had had the stroke. I thought it was because, if I remember correctly, it was the start of a urinary tract infection again. And from there, because I was in the hospital back in August, and I got a urinary tract infection and stuff like that. And I wouldn’t wish it upon my worst enemy. [00:05:15] back in August was the first time I ever had a urinary tract infection. And it’s not a very comfortable feeling.

**Q:** Sure, sure. And one thing I wanted to ask you is, I think I had mentioned to you yesterday that this finding of the stroke was, in some ways, an incidental finding, or your doctors were looking for something else, and then they found out that the stroke was present. So have you-- Do you have a sense for what that means, when something is incidental?

**A:** I don’t understand what you're saying.

**Q:** Okay. When something is described as being an incidental finding, what does that mean to you?

**A:** They weren’t looking for that. They were looking for something else. They came across that.

**Q:** Okay. How does that make you feel when something is kind of found unexpectedly or incidentally?

**A:** It doesn’t bother me, but at the same time, it’s good that they did find it in case something happens again, or it gets [00:06:32].

**Q:** So I talked to you a little bit about some things, and actually the internal medicine doctors have probably given you some advice as well. With respect to the stroke, what sort of advice were you given?

**A:** Just basically watch certain things that could happen. Like I said, I always knew that the left side controlled the right, and vice-versa. I can't think of what else to remember, what to look for, in regards to somebody--

[side remarks]

**Q:** Okay. So just continue from that frame. What I wanted to ask is, so you're talking about how there are certain things to look for. Are you referring to symptoms of stroke?

**A:** Yes and no, because some things I do know to look for, other than-- I have no idea what to look for.

**Q:** As part of this interview, what we’ll always do is give you a little bit of stroke education afterwards. So I’ll talk to you about those symptoms to look for following this. I think I had mentioned some of them a couple of days ago, but it’s perfectly understandable not to remember all of them. I'm going to give you some information on that following the interview.

**A:** Okay.

**Q:** Having heard that you had a stroke, one without any obvious symptoms, do you have any concerns about that? What are your concerns about having that diagnosis?

**A:** Being told-- should I say a slim [00:08:33] stroke scared the life out of me. I didn’t know what to expect. But once I heard “stroke,” I thought I was either a goner or certain things were completely-- like I said, all I could think of was my aunt, left side is dead, can't speak. But then, you know, I didn’t give the time for somebody to tell me what was going on, you know. And maybe I jumped too fast into it, rather than letting people explain. And I'm always one, “Oh, I’ll just do it that way, just take care of whatever this is right here,” instead of asking what are the outcomes? What can we do here to try to fix it? Instead of hitting me with one thing, hitting me with five things of how to take care of it and fix it.

**Q:** So it sounds like it made you feel pretty scared.

**A:** Yes, because I never-- never expected to hear something like that, because I already knew that heart and cancer ran on both sides of my family. So hearing about stroke never occurred to me. I gave it no thought.

**Q:** Did it make a difference that this was a stroke that did not have any symptoms, as opposed to one that has symptoms? Does that distinction make a difference to you?

**A:** No. I mean I know I'm not trying to be mean, but if something’s going to happen, it’s going to happen. You can't prevent it not to happen. If I'm about to take a heart attack right here and now, there's no way of me stopping it, other than you doctors helping me. You know, I do have one request, that he was my mother’s heart doctor over at Mass. General. But the only way I can try to prevent it from going further than what it is by the doctor’s care, doing what they can, so it doesn’t go further than what it went. You know, I don’t think nobody asks for something major to happen to them. And it is, I mean I can't speak for everybody, but I think there's that scary feeling there in the person.

**Q:** Okay. So now that you’ve had the stroke, do you think it puts you at risk for any other medical problems?

**A:** I don’t know. Sometimes I wonder. Like when am I going to get the next?

**Q:** Okay.

**A:** You know.

**Q:** I'm not expecting you to have any background medical knowledge, but I'm going to give you a list of symptoms or medical conditions and ask you if you think that there's any connection between them, or if you think that having the stroke without symptoms puts you at risk for these issues. And there's no right answer or wrong answer. I'm just enlisting your opinion. So for which of the following do you think you're at a higher risk, now that you have this diagnosis of a stroke without symptoms?

So the first is, a stroke. Do you think you're at high risk of that?

**A:** From having the stroke? I wonder, because I mean I never had one before. But other than small ones that they told me that I have. But I'm not going to let it get the best of me.

**Q:** Okay. Do you think you're at risk of having what's called an intracerebral hemorrhage, which is a bleed in the head?

**A:** Is than aneurism?

**Q:** An aneurism can cause that type of hemorrhage, but it’s not the only thing.

**A:** No, the only reason I'm asking is because my aunt had one. And that’s how she passed away.

**Q:** Oh, I see.

**A:** And her daughter, my cousin, has one. But she has to get it checked every so many months. So that’s why I was asking.

**Q:** Okay.

**A:** You know, I'm not trying to sound mean, doctor. But when my time is up and He wants me, I'm coming.

**Q:** I understand. I understand. I don’t mean to upset you.

**A:** No, it’s not you, doctor. You guys have been fantastic to me here, great to me here.

**Q:** I'm glad that you feel that your care is good. How about heart attack? Do you think that having stroke puts you at greater risk?

**A:** Heart attack scares me, because of both of my parents having them, my mother had-- my father had three, plus a quadruple bypass. My mother had a massive. And for both of them, my mother lived 30-some-- 32 years to the day. My father lived quite some time. So of course, a heart attack is always on my mind. I think about it.

**Q:** Do you think that’s connected to stroke?

**A:** I'm a little leery on that.

**Q:** Okay. How about high blood pressure? Do you think having a stroke puts you at risk of having high blood pressure?

**A:** No, because I never-- coming from a large family, I remember it’d be only two of us, and they're cousins that have high blood pressure.

**Q:** Okay. Do you think the stroke puts you at risk of having high cholesterol?

**A:** Cholesterol is always the one that’s with me.

**Q:** Okay. How about diabetes? Do you think the stroke puts you at high risk of having diabetes?

**A:** I don’t have that neither, which I thought I would have had that. My doctor and I was completely sideswiped, I want to say give or take a few years back, because he got a reading back from my bloods, and we thought that I had blood, um, sugar diabetes. And I didn’t. I mean on my father’s side, my father was one of 15. And there's one, two, I think there's like four of them have the sugar diabetes.

**Q:** I see. Let’s focus a little bit on the stroke that’s happened and what effect that might have on your health. Do you think it puts you at risk of having headaches?

**A:** At times, yes. And a lot of times, if I get a headache, they're not big, huge ones. And I try to go as long as I can without asking for aspirin or a Tylenol.

**Q:** Okay. How about vertigo, sort of like a spinning sensation. Do you think the stroke will make it more likely that you’ll have that?

**A:** I don’t remember that ever happening.

**Q:** Okay. How about seizures, when people stiffen and shake?

**A:** No.

**Q:** Do you think this stroke might make that more likely?

**A:** Never happened to me.

**Q:** Okay. How about in the future, do you think that will be a problem?

**A:** I hope not.

**Q:** Okay. How about falls? Do you think that this stroke is--

**A:** That goes without saying, for me.

**Q:** I know that you’ve had falls. But do you think that the stroke might make that worse?

**A:** Yes and no, to the extent of just pay attention to where I'm walking.

**Q:** Okay.

**A:** And make sure somebody is with me.

**Q:** How about memory loss? Do you think the stroke is going to make that worse?

**A:** Probably, but I don’t know.

**Q:** Okay. Dementia, do you think the stroke can put you at risk for that?

**A:** That’s the way a lot of people are going now, with the dementia, and going all the time. And I just hope it doesn’t happen. I mean it’s probably going to happen to me anyways, but I just hope it doesn’t happen to me for a long time down the road.

**Q:** Okay. How about anxiety? Do you think stroke could make that worse? It’s like when you feel very worried about a lot of things.

**A:** Oh, I'm always worried about things, doctor. And I can't say it’s because of everything that has happened to me. I'm one of those ones that worry. You name it, I worry about it.

**Q:** How about depression? Do you think the stroke would make that worse?

**A:** I’ve caught myself being depressed plenty of times, not to the extent of going to do something stupid and hurt myself or hurt somebody else, but I have caught myself into a very, very sad[?] mood.

**Q:** Do you think the stroke would make it worse, though, or more likely for your depression to be bad?

**A:** No. Once it sees me doing what I want to do, I think it might just help me pull out of that situation.

**Q:** Okay. Last thing is schizophrenia. Do you know what that is?

**A:** I heard of it, but I can't think of it.

**Q:** Okay. I was just wondering if you thought stroke might put you at more risk for that.

**A:** What is it?

**Q:** It’s when people have sort of a distorted view of the world and might have hallucinations and other problems and behavioral problems. It sounds like you're not familiar with that. So that’s okay. So we’re getting close to the end of the interview, but I wanted to ask you just a couple more questions. Is there anything-- Now that you know that you have this diagnosis of a stroke, where you didn’t have any symptoms related to it, sometimes we call it a silent stroke or a covert stroke. Is there anything that you think you’ll change now?

**A:** Oh yeah, there's a lot that I'm going to change. And I’ll just get myself back to where I need to get back to. Not that it’s never going to happen again, or whatever, me to get sick again, but if I don’t do it now, it might be too late down the road.

**Q:** What do you think you’ll change?

**A:** My eating habits, things that I need to do to try to stay healthy.

**Q:** Like what?

**A:** I never knew how to say no to everybody. I always put myself on the back burner. “Oh yeah, I’ll take care of that. I’ll take care of it myself later.” But like that saying goes, one day at a time.

**Q:** So you want to focus more on your health as opposed to taking care of other people?

**A:** Yes.

**Q:** I see. Okay. One thing I think it’s important to know is that there aren’t any current national guidelines or specific research studies that guide physicians, doctors, in selecting tests or therapies for people who have silent strokes or covert strokes. Knowing that fact, how concerned would you be about that? Does that bother you?

**A:** Before I give you an answer, doctor, is there a difference between the silent stroke-- should I word it as a noisy stroke?

**Q:** That’s one way of describing it.

**A:** But, I mean, I think--

**Q:** So you're asking is there a difference between a stroke without symptoms or stroke with symptoms?

**A:** I think people would like, if it happened, I think people would like to know that there is a stroke happening to them that can give them some awareness of what's happening, so if there are people around them, they can try to get some help, compared to noticing them and they don’t know what's going on. And then they’ve probably got people standing there, looking at them like, “What's going on? Why are they acting like this?”

**Q:** So it sounds like that is, that the idea of strokes without symptoms are a concern to you.

**A:** Yes.

**Q:** Okay. The question that you asked about whether or not strokes with symptoms-- you kind of described it as being noisy strokes versus silent strokes or strokes without symptoms. That’s actually a question that we don’t have 100 percent knowledge on yet. And there might be a disagreement between doctors in terms of what's the right thing to do in that setting. For me, I have a particular opinion as a stroke specialist. But other doctors might have different opinions. And so that’s a very good question that you're asking.

**A:** Well, too many people have the same-- even I might have the same challenge as someone when you guys, four others over here, that don’t feel the same way.

**Q:** Knowing that there might be this disagreement or different opinions, and that there aren’t really guidelines in terms of figuring out the right testing or the right treatments, how important is it to you that this condition be studied more?

**A:** [00:23:22] be studied more, so the doctors and whoever’s involved get more information of what they can do to help somebody that [00:23:38] a seizure. I mean on the silent one, you don’t know at the time that it’s a silent seizure or a stroke.

**Q:** Mini stroke, yeah.

**A:** So how can you help the person, when you don’t know what's really happening inside their head?

**Q:** Yeah, that’s a good point.

**A:** You know, unless you find out all the information to see what to do in regards to it.

**Q:** So we’re hoping in the future to do studies like this to help people know what's the right thing to do. If there were studies that found that there were some modest or relatively small health benefits with medications or specific lifestyle changes like exercise or diet or something like that, do you think you would be motivated by the studies to change what you're doing, whether it’s taking a new pill, or changing your lifestyle?

**A:** Exercise more.

**Q:** Yeah, do you think you would make a lifestyle change or take new medications if studies found a relatively small benefit, in terms--

**A:** It might take time, but yes, yes, as long as it’s not drugs.

**Q:** I'm sorry?

**A:** As long as it’s not a drug.

**Q:** Not a drug? You wouldn’t want to take more medications?

**A:** I don’t know what happens, we’ll see down the road.

**Q:** So for example, what if a study suggested that you should take an aspirin or a cholesterol medication?

**A:** That’s fine.

**Q:** Do you think that that’s something you’d be willing to do if the benefit was relatively small, but there was some benefit?

**A:** Yes.

**Q:** Okay. So we’re actually very close to being done right now. I wanted to know if there were any thoughts that you have or any questions that you might have about strokes without symptoms?

**A:** No.

**Q:** As we’ve been going through these questions, has your perception or understanding of this issue changed?

**A:** No.

**Q:** No? Okay. Is it something that you're more worried about now, or as worried as you were before?

**A:** I'm a little bit at ease, you know, maybe I was looking too heavy into it, of losing to Talmud, having no more shaking in my hands. I forget what happens to the right hand, but how your left hand can go dead, or you're dragging your left foot, that’s how I was looking at it. And I know that there was probably more than one stroke, but I never bothered to look into it, or what to look for. I mean like even looking at people with cancer, my father was lucky. He didn’t lose-- if he lost any of his hair, it was very, very little. He didn’t lose no weight. And then I look at other people, three of his brothers didn’t lose a pound, didn’t lose a strand of hair. And, I mean, one of my uncles was really lucky [00:27:11].

But how can they give a date when they're going to leave you? I mean I know cancer is a bad disease, but some of them-- when you look at people you do know that lose their hair, they go from 150 pounds to 105. Where did all that weight go to? And I know there's all kinds of different-- It was very hard for me to deal with.

**Q:** Right.

**A:** You know, like we all-- like I guess my brothers, we all deal [00:27:58] with both mom and dad. Even though they passed away many years apart, but at least we can go on saying we had both our parents. And it’s a very-- I have my days, she’s like you guys have your days. And then I go back and talk about them every day of the week [00:28:19].

**Q:** Let me ask you to clarify two short things. You mentioned that you're more at ease now, after we’ve been talking. What makes you more at ease now?

**A:** In regards to?

**Q:** The stroke.

**A:** Because I'm learning more now than I've ever known. Because the only time I knew about a stroke was when my mother had it. I didn’t know that I had taken one in here. I didn’t know that I had taken one or two prior of my way in here, whether or not it’s all in that until now. But I never ever knew it. And there's something-- The only time that something was said to me about a stroke was the other day, yesterday, when the doctor told me that I had a stroke. But up to then, I never knew that I had one.

**Q:** Okay. Well let’s stop the recording here right now, I wanted to thank you for doing this interview.

END OF INTERVIEW

**Participant 2**

**Q:** I'm going to start the recording right now. And so this is our second patient that we're interviewing today. And I'm going to ask you about eight questions, but there might be some side questions that I'll incorporate along the way. So, the first question is just a very open-ended one, but it’s just tell me what you know about your diagnosis, or the issue that you have?

**A:** I guess what I do know is that I have white matter disease, and I believe there was 12 spots. I also had supposedly six strokes, mini strokes, that I had. And I have a slight bleed. That's what I know of.

**Q:** Is there anything in particular that-- anything else that you'd want to share about that right now? I'll ask you some more questions, too.

**A:** Well, no, just very worried about maybe getting dementia eventually down the road, and only because my primary care actually told me that.

**Q:** So, who is the first person who gave the diagnosis to you and what were the circumstances?

**A:** The first person-- I went to the county hospital to have an MRI and I was to see if I had sarcoidosis in the brain because I have it in other parts of my body. And the first doctor told me that everything was fine, and they didn't find anything in the scans. And then after that, that doctor left and a new doctor came in and he basically said the same thing. He was reading it off the report. And when I asked him to take a closer look at the x-ray, he kind of panicked a little bit and sent me to Tufts where I spoke to Dr. L, who basically gave me this information. It actually caused a lot of confusion because one doctor said there was nothing, another doctor after looking at the scan was concerned, and then after speaking to the doctor at Tufts, it’s just very confusing.

**Q:** So, there are a lot of names, or a lot of terms, that people use for these findings on the brain scans. And I think you mentioned a few of them, but what were the terms that were referred to you? How did people describe these findings?

**A:** I think the terms were very technical, and long words, so I don't remember a lot of them. And that's one of my problems, I have a bad memory. But what I do remember is white matter disease and possibly dementia and ischemia and a bleeding, I guess. That's what I remember.

**Q:** Sometimes, people will use terms for these that include-- I'm actually going to mention a few of them and see if any of these were used. Some people call them silent strokes or silent brain infarction, silent cerebral infarction, covert stroke, covert brain infarction, sub clinical stroke, subtle stroke or asymptomatic stroke. Were any of these terms used?

**A:** I believe silent stroke.

**Q:** And you mentioned there are at least three doctors that you encountered. So, what type of doctors were those that helped-- or that brought up this diagnosis or brought up these findings?

**A:** At the county hospital, there were neurologists as well as at Tufts. They were all neurologists.

**Q:** You mentioned that you had the scan in the setting of looking for sarcoidosis in the brain. Was that prompted by symptoms that you had or was it sort of a screening test?

**A:** More of a screening because they found a sarcoidosis. They believe they found it on the outside of the heart, and they found it in my lungs, they believe it’s in my skin. And they wanted to put me on prednisone, steroids, and my primary doctor doesn't think I should do that because it’ll go to diabetes. But they wanted to see if it was in my brain, and if it was, then there'll be no question that I would actually go on these drugs. But it was not found in my brain and they're still trying to get an MRI on my heart. I'm kind of on hold with everything right now.

**Q:** And when these were found, did anybody use the term incidental or unexpected when describing the findings on the scans?

**A:** The heart doctor at the Carney who actually looked at the scan, he was the one that actually brought it to my attention, that there was more wrong than what I was being told. And that's why I went to the second neurologist and I asked him to look at the scan because the cardiologist looked at it as well. And he said that the symptoms that he sees on the scan, it was of an 80 year old person, that I had the brain of an 80 year old. And that there may be concern that I'm only 57 and there was worry about that. I have a 96 year old aunt who has sundown dementia and I just kind of panicked when I heard that.

**Q:** Sure, that's understandable. I'm going to pause very briefly just to check on the sound, okay?

**A:** Okay.

**Q:** So the next question is what advice was given to you by your doctors when these findings were discovered on the scans?

**A:** Let me see, I'm thinking of how far to go back here. The second doctor who actually did look at the scans after the heart doctor told me there was a problem suggested I go to Tufts and meet with his colleague, somebody who would know more about this. He did seem very concerned about it. I'm not sure. I actually forgot the question, I'm sorry.

**Q:** I was just wondering if there's any particular advice that was given to you? So it sounds like the second doctor who, I believe, was a neurologist, right?

**A:** Well, before the second doctor, I spoke with the cardiologist who actually said there was an issue on the brain scan. Wasn’t sure what it-- they told me there wasn’t, and then I saw a different neurologist at the Carney because the first one left, and he again was concerned and sent me to Tufts.

**Q:** So the advice--

**A:** So they basically-- sorry.

**Q:** Go ahead?

**A:** The third doctor at Tufts basically said not to worry right now, and I kind of felt that they got the impression that he should do the worrying and I should not. So I'm trying to think differently than I was. I was in a panic, I was in a big panic. We're both sitting, me and my partner, are both hitting retirement age and we want to plan our retirement out. We don’t know where we're going at this point. I know it’s a fact that there's something wrong and I'm going to have problems later on in life, of possible problems, we want to retire now while we're younger and so we can have some time together. But if it’s not an issue, then we’ll just wait until he’s 67, which will be about six years.

**Q:** What are your concerns about having this diagnosis?

**A:** Basically, I'm going to dementia or having a major stroke. I mean, these were minor strokes that I didn't even feel, but I'm always-- any time I get a headache now I'm concerned. With the heart problem and the breathing problem, I just worry. I'm more afraid of-- somebody said to me the other day, “Are you afraid of dying?” I'm not afraid of dying, I'm more afraid of living and have to depend on somebody to take care of me because I've had a stroke.

**Q:** Understandably. Do you think that this diagnosis is connected to your other medical conditions?

**A:** From the reading I've done on the internet, and I probably shouldn’t be doing this, but I do go on and check out-- use [?] Doctor M.D., there's two things I read. One was that this could be caused by a congenital heart defect that I had when I was four years old and had heart surgery. Or, it could be a connection with the afib kicking up blood clots that might have caused this.

**Q:** Okay. In general, after learning about-- I think you've answered this question already because you've been very eloquent in your explanation, but in general how did you feel about-- after you had learned that you had this diagnosis?

**A:** Kind of despair, I guess, is what I was feeling because I do have all these-- I have heart, lung, kidney, bladder disease. I have all these diseases and then the sarcoidosis, diabetes, high blood pressure, and I thought if anything was going to be clear, it was going to be my mind. But I did notice a couple of years ago, I started forgetting things and saying things and I'm having trouble retrieving information, stopping in the middle of a sentence. So, I knew something was wrong, but I thought it was just stress, or maybe part of getting old. So, I was really shocked when it came back that it was a problem. But probably not as shocked as I really thought I was, if that makes any sense.

**Q:** No, that does make sense. My next question was actually going to be asking you about conditions for which you think this might put you at risk, but I think you've already explained that you feel this puts you at risk for a serious stroke or for dementia. So, what I'm going to do, actually, is I'm going to give you a list of potential conditions or symptoms that might be related to the silent strokes and white matter disease. And the framework for that question is for which of the following do you think you're at high risk now that you have this diagnosis? In other words, do you think that the silent strokes put you at risk for these conditions that I'm going to list?

So you can say, for each one, you can say yes, no, or give some qualified answer somewhere in between that. So you ready?

**A:** Yes.

**Q:** Great. So the first one is stroke?

**A:** Yes

**Q:** Second one is intracerebral hemorrhage, which is a bleeding into the brain tissue?

**A:** Yes.

**Q:** The next one is heart attack?

**A:** I would say no.

**Q:** The next one is heart failure?

**A:** Actually, I need to change that answer before. I'd say yes and yes to heart attack and heart failure only because I'm afib and the connection with this problem.

**Q:** Okay. The next one is high blood pressure?

**A:** Yes.

**Q:** After that, the next one is high cholesterol?

**A:** I would say no.

**Q:** The next one is diabetes?

**A:** Yes.

**Q:** The next one is headaches?

**A:** Yes.

**Q:** The next one is vertigo, which is the sensation of spinning?

**A:** Yes.

**Q:** The next one is seizures?

**A:** No.

**Q:** The next one is falls?

**A:** I'm sorry, say that again?

**Q:** Falls, like tripping and falling?

**A:** Oh, fall? Yes.

**Q:** The next one is memory loss?

**A:** Yes.

**Q:** The next one is dementia?

**A:** Yes.

**Q:** The next one is anxiety?

**A:** No.

**Q:** The next one is depression?

**A:** Yes.

**Q:** And the last one is schizophrenia?

**A:** I would say no.

**Q:** Okay. So the next question is now that you've learned that you have this diagnosis, is there anything that you think you'll change?

**A:** Yes.

**Q:** What sort of things do you think you'll change?

**A:** I've already started changing diet, trying to keep my blood pressure under control. Just trying to stay calm, let things kind of roll off. Go to church, trying to do some spiritual-- something I haven't done for quite a while. I'm just trying to find peace in my life right now, and also spending time with my grandson and just trying to do things that are fun and just try to stay out of stress as much as I can.

**Q:** Those sound like good things to do. The next question is a hypothetical question. So, if I were to tell you that there are currently no national guidelines or no specific research studies that guide physicians in selecting tests or therapies for silent stroke, how concerned would you be about that fact?

**A:** Could you say that again, I'm sorry?

**Q:** Sure. It's a complicated question, so I'm happy to repeat it as many times as is needed. The idea is hypothetically if I told you that there were no guidelines on a national level or no specific studies that guide physicians in selecting tests or treatments for silent stroke, how concerned would you be learning that?

**A:** I'd be very concerned. You know, how can I be treated if a doctor has no guidelines or any studies in the past? So it’s very concerning.

**Q:** How does it affect your perception or your trust of the advice that doctors have given you?

**A:** Oh, it affects it very much, I would say, because if they don’t know, how am I supposed to know?

**Q:** For you, how important would it be to have this condition studied more?

**A:** In my opinion, it would be much more because I'm 57 years old, I have this stuff going on. I would like to know if I did something in my past that may have caused this. I never drank or anything, alcohol, I never did drugs. I just don’t know where all these health issues are coming from, the brain as well as the rest of the stuff going on in my body. So it would be really important to know if I did something to contribute to this.

**Q:** Right, that makes sense. Also, in this hypothetical framework, let’s say that there are some studies that are done to try and figure out the best way of preventing the health consequences that might follow silent stroke. And let's say they were to find some benefits in terms of health, but they're relatively small benefits related to being on a medication like an aspirin or maybe some lifestyle changes like what you're describing with diet and exercise. If these studies show that there was benefit, but it’s a relatively small one, do you think you would still be motivated to make these changes, like taking a pill or changing your lifestyle?

**A:** Absolutely. I really believe I'm headed in that direction. I'm on a diet and exercising more and I'm trying to do the very things-- yeah, I definitely would. There would be no question I would do whatever I can to live as long as I can in a productive way.

**Q:** So every little bit counts, that's kind of the way that you would approach this?

**A:** Exactly, exactly.

**Q:** Okay. So we're almost done with the interview. I want to ask-- so I've asked you a number of questions to help you explore this topic. And after being asked these questions, do you think your perception on this topic, on silent strokes, do you think that's changed at all?

**A:** Maybe a bit. I mean, I didn't realize that there wasn’t studies being done, or more being done to help people with these silent strokes. And I think any education is something, you know. And I did get a little bit out of this today just by you asking the questions.

**Q:** My last question is a very open-ended one, but do you have any additional thoughts or any questions about this or any-- if you have questions, I'll answer them afterwards from my perspectives and what I know about the field. But I wanted to see-- I wanted to give you a chance to voice your thoughts.

**A:** Okay. My thoughts are is that I know you don’t have a crystal ball and it’s hard for you to predict this. But has it shortened my life, is my quality of life going to be interrupted early in life? Like I said earlier, I'm only 57. I have a million questions. Am I going to get dementia like my primary care said? She says I have to keep my blood pressure under control. I kind of put it back on her because they're the ones writing the prescriptions, I'm taking the prescriptions. So if my blood pressure’s not under control, it’s really up to them to help me get it under control.

And the diabetes, I'm really working on that hard, too. I went from 50 units of Lantus down to 20 recently, and that was just because I made a slight change in my diet and exercise. So, I think I lost track of what that question was.

**Q:** It's just very open-ended, it’s just if there's anything else that you're thinking about on this topic, or if there are any additional questions you might have?

**A:** I'm thinking of dying young, that scares me. Especially now having grandchildren. I have a great life with a great partner, we've been together for years. We have a couple of dogs and a nice house and things are really going good for me in life right now. And the thought of struggling with all these health issues is really tough. And then to have this sprung on me, too, it’s just-- and like I mentioned earlier, my 96 year old aunt who has sundown dementia, and how she goes in and out of life. It just sounds-- I know I make a lot more mistakes now. I think I'm just more noticing those things. But family members and friends have told me they’ve seen the difference, too, so that's concerning, you know.

**Q:** I think you've explained pretty clearly your concerns and thoughts. If I'm hearing you correctly, some things that are very important to you are making sure that your quality of life remains good and that there's no progression or worsening of your symptoms over time?

**A:** Exactly.

**Q:** It sounds like you're worried that silent strokes might contribute to that.

**A:** That and also the ischemia and the bleeding and the afib. I mean, there's a lot of things going on. I've lived a pretty healthy life. That's why I'm really surprised this is all happening. But having ASD repair in 1962, which is quite a few years ago, I think I've kind of dodged a bullet. I do remember the doctors told my mother in front of me that I would probably would live to be 25, and I've already doubled that and I'm hoping to triple it.

**Q:** I think that's a good goal. So, I'll stop the recording right now and then give you a little bit of a debriefing and then I'll answer any additional questions you might have about this, okay? So, going to be one second. I'm just going to stop the recording.

**A:** Okay.

END OF INTERVIEW

**Participant 3**

**Q:** So let’s get started right now. So the first question is-- a lot of these are very open-ended questions, but this first question is just tell me what you know about your diagnosis?

**A:** My diagnosis in general, all encompassing or the new one when I first saw you?

**Q:** You can start from the beginning, if you'd like?

**A:** Okay. So my diagnosis is acromegaly, because I have pituitary brain tumor that was growing for approximately ten years before it was discovered. And with that comes other problems, but generally when I go to the doctor or I'll see Dr. S in endocrinology next week, is acromegaly. So that's like the whole thing, and then now I have the other brain stuff because I thought I had a stroke because I'm a spazz, but it’s better that I thought I had a stroke because now I don’t eat bacon anymore.

**Q:** Let me just check the volume, sounds good.

**A:** Okay. And then the other portion of that is the damage that you showed me on my most recent brain scan from where I had the radiation and possibly the damage from the violent migraines I used to have that were a component of the brain tumor.

**Q:** Okay. So, maybe walk me through a little bit in terms of how this was found or how this problem was discovered?

**A:** It would have been found a lot sooner had I been going to a place such as Tufts. But in Connecticut where I lived, you had one doctor for this, one doctor for that. You didn't necessarily, where I lived, go to a hospital for your doctor care. So, really I had a bunch of doctors that didn't talk to each other and a lot of symptoms that presented that could easily be explained by something else. Oh, you know, she has ovarian cysts, oh migraines. That has to do with-- they couldn't really figure out the migraines, but that has to do with something else. Oh, there's-- I had like prolactin was an issue. I didn't know it at the time. But I would have a lot of like milk in my breasts that would be like, why is this leaking? And they're like, oh well, that's connected to the polycystic ovarian system. Like, no one ever connected that it could be a pituitary issue.

And it wasn’t until I went to my new general practitioner at the time, because the migraines were so violent that I couldn't get the doctor I had been going to, because I usually would have the doctor near where I worked. She said, “I can't believe you've been going through this for this many years and no one ever asked you to have an MRI.” So that's really how it was discovered. And I was working in conjunction, or she was working in conjunction, with an endocrinologist that she recommended I see because of the polycystic ovarian syndrome. And it was from the endocrinologist, and I don't know if you want me to talk about that now or later, who scared the crap out of me with the way she told me I had a brain tumor. So it’s definitely the way not to do it.

**Q:** So, at some point in time, before you came to see me in the neurology clinic, you had gone to the emergency room.

**A:** Correct. I had gone to the emergency room, I don't remember the date now.

**Q:** That's okay.

**A:** But it was definitely before Thanksgiving, I'm pretty sure, because I had been having problems with my left arm and because my mother had been an RN for over 50 years and she herself at one point had a stroke in 2009 because she was an older mom who smoked. I was aware of symptoms of a stroke, and symptoms of a stroke for women, they're different than, like, what necessarily a guy may have. So, I'd been having problems with my left arm and I thought that perhaps it was a pinched nerve.

But when my arm went, like, not numb but fuzzy and unresponsive, and though I could still open and close my hand, or whatever, when I would put it on my leg, it would just fall off, like the way I had shown you. And I came to the emergency room because I thought I was presenting with the symptoms of a stroke because not only did I have that going on, I had a headache that had come out of nowhere. And so I'm like, “Okay, I've got a tumor over my pituitary-- over my carotid artery, I've got this goofy, fuzzy arm, I've got sweaty, clammy hands because I think at that point I'd whipped myself into a frenzy, and I have a headache. I'm having a stroke, I should go to the emergency room.” So that's how I wound up here.

**Q:** And what was that experience like when you were in the emergency room?

**A:** It was not good. The triage, no offense to you personally, the triage at Tufts when I got here at the ER was horrible. Because I came in and I said, “I believe I've either had a stroke or I'm about to have a stroke.” And the security desk girl was like, “Okay, well fill out that sheet.” And she shows you like the little blue piece of paper. And I couldn't focus because you're just somewhere else. Like, am I going to drop dead filling this out? And I was like, “Okay, it’s filled out, but when can someone see me? I think I have had a stroke.” And she was like, “Uh, they’ll see you when someone calls you when they pick up the piece of paper. Take a seat over there.”

And I found that very contrary to every other experience I've had with Tufts. And God forbid I actually had had a stroke and she's just like, “Whatever, put your little form in the basket.” And I had died, thank God I didn’t. So that didn't set the tone off because I'm just like, I'm sitting here in the waiting room, they're taking these other people. They all look like they're fine. I need to see someone. And I checked back and she was like, “You know, I told you someone’ll take the piece of paper.”

And it’s not like I was being obnoxious, but I was concerned because I know if you are having a stroke, you have like windows of time for things. And I know that I already have like a compromised brain, so I can't have a whole bunch more cells dying off because she can't be like, “Hey Nurse Jane, this girl thinks she's having a stroke.” So that was very stressful.

I was in what I would call the older part of the emergency room, which is fine, a bed is a bed. And I was next to a nurse’s station. But it didn't seem like there was a lot of patient management in terms of the guy in the bed across like the street from me, they were playing rap music on their phone, like really loud. And finally I had to say, “I'm sorry to be a bother, but could you please tell them to turn that off?” And it was-- it added to the stress because the first doctor who saw me, I think, was from the psychology or psychiatric department because he was on the rotation to the emergency room.

And I felt that some of his questions, yes I understand you have to answer a set, generic list of questions, but it was very difficult because I didn't get a sense that he was understanding what my history was. So thank goodness, he did get someone else to assist, but it was a very long process. I was there from about 10:30 in the morning, and I didn't get out until 9:45. So, it was just--

**Q:** What did they tell you you have, or how did they explain your symptoms?

**A:** Well, when I say his name was Dr. M who was like the official, “I'm in charge of the emergency room guy.” When he came over, because everyone did the same thing. Everyone’s like, “Squeeze my hand, squeeze it.” And I'm like, “Okay, the last 14 doctors had me do this. I can do this, you know.” And I know they have to do it, I understand that, I get it. But thank goodness the doctor before Dr. M, whichever one that was, who at first said, “Oh, it’s just probably a pinched nerve,” because my blood pressure was fine, my finger, my oxygen and everything, and my blood was oxygenated. Once I said that I'd had the tumor, the brain tumor in the carotid artery, everything changed and I secretly wished I hadn't even said that, though they would have found that as soon as they brought up my Tufts card number in the system. Because it was like no one quite knew what to do with me at that point.

And again, we determined I hadn't necessarily had a stroke, so I'm no longer a high priority patient, but I still need a little more info. I need someone who’s going to be like, “Hey, I'm your guy. I know we're really packed in the emergency room. Here's what we think, get you up, get an MRI, whatever.” But everything took so long and that was hard. And again, I understand emergency rooms are for emergencies and if someone comes in in an ambulance, they're an emergency. I walked in from the Orange line, so you know.

**Q:** So you had the experience in the emergency room, eventually an MRI of the brain did occur. And you actually referred to follow-up with me and the stroke neurology clinic afterwards.

**A:** Correct.

**Q:** Was I the first person who talked to you about the spots or abnormalities on the scan, or was it somebody else?

**A:** Yes. No, you were the very first person. Because after the MRI was done, it was hard because-- because they didn't know what they would need to do, I wasn’t allowed to have anything by mouth. So no water, no food, anything. I had eaten breakfast at like 8:30 in the morning. It’s now three. I'm dying of thirst and I'm starving, so I'm even more cranky. So, when they did my MRI and I became nauseous and I had to end it early, as I told you, because they gave me like an Ativan push and I hadn't had any food, all they did was the nurse came over and said, “Well, the doctors are trying to decide if they should discharge you. One wants to admit you and one’s like it’s okay for you to go home.” So that doesn't give me any information.

Well, one thinks I'm dying, and one’s like, “Go with God. If you get home, that's awesome.” So, I didn't know what the basis of their disagreement, their different points of view. Not that I should even know that because I'm not a doctor. But just come in and say, “We saw some stuff. Nothing to be alarmed about. We're going to send you home, but we want you to follow up with you.” Finally, it was just someone came, was like, “Okay, you can go.” And I'm like, “Okay. I'm supposed to have a doctor’s note, I need a prognosis, I'm supposed to have a prescription?” “Well, I don't know anything about that.”

And I get it, it wasn't a shift change yet, but still take ownership. But again, that makes me sound like a horrible person because I know in an ER, there are a lot of things going on. But really, I think because I was raised in that environment and I'm from Connecticut down in the New York area of Connecticut, things are different. And it just seemed really-- this is the second time since I've lived in Massachusetts-- I moved here in 2009-- this is the second time I've been in the Tufts ER, and both times-- once was in 2010 and once was-- both times were like chaotically disorganized.

**Q:** No, I think it’s very good to hear that both actually for the study as well as for quality improvement purposes, and that's certainly something we can address. Moving ahead a little bit, when you came to the clinic, we looked at the scans together and--

**A:** We did and that was kind of creepy for me because even though my mom was a nurse, I have no-- like, I was super brave. It grosses me out seeing stuff like brains and stuff. I mean, I understand it’s mine, but that-- so though I loved that you did that and I got such a sense that you truly wanted me to really understand what was going on. And I can't even tell you how much I appreciated that. When I called my sister I said, “You know, our mom-- Mother would love him because he was so thorough in how he explained everything.” And you weren't like a fear-monger, like. “And then look at this dot.” So, thank you.

**Q:** Appreciate your honesty, I'll go with that. [laughter] At that time, I know something that I was faced with was figuring out the best way of explaining that to you, and that's part of the reasoning for this type of study. But I described to you a number of possibilities. Because there is one spot, it’s only a single spot on the scan, that can-- that could be described in a number of different ways. And so we went over your medical history a little bit, tried to come up with potential explanations and also potential pathways or things you might do as a result of one explanation or the other.

**A:** Right.

**Q:** So I think the thing that I mentioned as being the most concerning possibility is that it could be that that spot represented a silent stroke or a stroke without symptoms, which can occur for a variety of different reasons. And when that does happen, oftentimes what we try to do is figure out what caused it in order to figure out ways of preventing it. And we also talked about how people with migraines often have more spots on their scans, too.

**A:** Which I did not know. You were the first person to tell me that.

**Q:** And there's some variability in terms of the field in terms of describing or hypothesizing whether or not those are meaningful, whether or not there's a symptom or significance in terms of health as a result of finding those.

And then the third part we talked about is because of the pituitary tumor, you had had some radiation therapy in the past, and sometimes people with radiation therapy will have spots on their scans that could be a result following that, that that's a common finding.

**A:** Yes.

**Q:** And so, one of the questions I was going to ask is if anybody had used any particular terms, or ways of describing it, but I think from what I'm recalling and what you're telling me is I'm the first person who told you that and the only one you talked to about that. Is that correct?

**A:** Correct, because-- and it’s twofold. The first is when I found this-- when they found the brain tumor, it was 2005. So, a lot has changed in ten years in terms of procedures, I would think the technology because all of my scans, and I was at St. Rafael’s, which did a lot of pituitary tumor surgeries because I was with the neurosurgeon who perfected the going up the nose thing. Everything was film, and as I mentioned to you, I have issues. So my mother, he’d be like, “Let me explain.” I'd be like, “Okay, will you let my mom know and I'm going to go out into the waiting room and I'm going to read Good Housekeeping,” because they would start to get, you know, medically, which is fine. And then she’d come out and she would just translate, and I'd be like, “Okay, cool.” Because to momentarily jump ahead, when I was going to have the surgery, and I may have mentioned this to you, he had everything out and he wanted to show me exactly how it’s done, or it was going to be done.

I said, “No, don’t show me because if you show me, I'm going to walk out and I will not have the surgery.” And he thought I was joking. And my mom’s like, “Don’t show her.” So, she went through, she took all the notes and she came out, she's like, “Nothing to worry about.” Explained it in super generic terms for me, kind of vague and I'm like, “Okay, fine, I'll do it.” And same thing with the radiation, when they put the-- bolted into my skull, I'm like, “Yeah, explain all that to her. I'm going back in the little waiting room.” And it’s just because things like that freak me out. Sometimes for me, too much knowledge is bad and I won't do it.

**Q:** Okay, fair enough. Let me pause for just one second. Make sure you're okay.

**A:** Sure. Nope, I'm good, thank you.

**Q:** Do you remember when I brought up the possibility of this being a silent stroke, do you remember what advice I gave you at that time or suggestions I had in case we wanted to go down that pathway?

**A:** I remember sitting on the exam table and you saying the words silent stroke, and then there were some other options of what else it could be. And I may have asked you a question, but I remember you saying, “Well, the only way we’d really know if it was a blood clot is if we went and did brain surgery, and we're not going to do that.” Something like that, possibly. Or, that's how I remember it, because I'm super paraphrasing, he was really good.

It was very difficult because generally I write things down when I go to medical appointments, and I didn't write anything down because I didn't know what to expect because I was always used to things being vague or, you know, Dr. S would just say, “Oh, you know,” because he would order MRIs because he monitors and he would just be like, “Oh, your MRI’s fine, nothing is-- it hasn’t grown back.”

So, I didn't think to bring anything. So, I honestly don’t remember part of the conversation after that because I was so stuck on the word silent stroke and my next thought was I probably should never have eaten so much bacon. And that honestly was my thought after you said those words.

**Q:** Okay. Knowing that that's just one possibility of what the abnormality on the scan could be, leaving the appointment, did you leave with any particular concerns about that potential diagnosis?

**A:** No, because I actually prefer the diagnosis of it was a silent stroke rather than it could be a blood clot. Because a silent stroke meant holy crap, you're super lucky. Let's not have this happen again. I've made a ton of life changes. You know, I message with Dr. T all the time on the portal. But, it could be a blood clot means you could die at any moment. So I don’t even think about that one. I just think, “Oh, it was a silent stroke and the hand of God reached out and touched me.” Or, “It’s radiation damage.” I don’t like thinking about it could be a blood clot.

**Q:** Okay. Let me ask actually to explain a little bit what you mean by that? So in terms of the distinction between a silent stroke and a blood clot and how-- what is the difference there?

**A:** Because to me, a blood clot-- and part of this is because my best friend for over 30 years, his mother died out of nowhere from a brain aneurysm. So for me, knowing that there could be a blood clot in my brain that if it burst, and I didn't get medical attention like ten minutes before it burst, chances are it’s going to suck afterward. I can't even think of that because if I do, I'm going to have to erase that from my mind when I leave here, it will paralyze me with fear and I will not do anything for fear of, “Oh, I'll jostle it and it’ll break.”

**Q:** I see.

**A:** So, I will stop living my life. So that’s why silent stroke is like, “Oh, silent stroke,” past tense, already happened. Warning sign, got the message, rocking it out in 2016.

**Q:** Okay, I think I understand where you're making the distinction now. Sometimes, we refer to strokes as-- stroke is a broad term to represent multiple types of diseases. And so one type of stroke we call ischemic stroke where a blood vessel gets blocked and usually by blood, small blood clots and oxygen and nutrients aren't going to part of the brain. So that part of the brain is starved of oxygen and nutrients. Whereas in another case, sometimes there's a blood vessel that for some reason gets weakened. For example, it’s an aneurysm and that blood vessel can break and rupture and there can be bleeding into the brain.

It sounds like when you say blood clot, you sort of mean that second category of some sort of bad bleeding into the brain, which is similar to that antidote that you provide.

**A:** Right. Because I always think blood clots can break off and blood clots can travel. And part of that is, unfortunately, knowing too much of that because my mom was a nurse. So, I sometimes secretly wish that I didn't know some of this stuff, that I just grew up thinking that other kids all knew, too. Because then I can over-think things.

**Q:** Sure. In a more general way, in terms of how you felt when you learned about this potential diagnosis, how did that make you feel?

**A:** The potential diagnosis that we just had?

**Q:** Yeah, like a silent stroke?

**A:** It made me feel-- this is going to sound so bizarre-- it made me feel grateful that it wasn’t a debilitating major stroke. It wasn’t a stroke that killed me. It made me feel like I was being given the gift of a second chance, that there was time to not necessarily right the wrongs, but to make positive changes so I wouldn't legit have a stroke. Possibly not the way most people would think about it.

**Q:** No, I think that's a reasonable response, yeah.

**A:** Okay. Because when I had the original diagnosis of the brain tumor, I did not want to have the surgery, I refused. And I said I would just rather wait for it to explode and one day I'm just like dead, and my mother wasn't having that. The woman never swore. Holy crap, I can't even say on your tape what she said to me.

**Q:** Fair enough. I think that is a very reasonable response and everybody responds differently, that's why we're interested in--

**A:** You have to make it into a positive. Because if you don’t, if you can't find the positive, and it really is like a gift, then again, it’ll go back to before. Like, oh my life is over and I can't-- I'm so fragile. It’s like, no, I had-- this happened, and it was wake up call and it’s never going to happen again, God willing, because I'm going to do everything in my personal power to make sure. And then if it still happens, I'll know that it was-- it would have been God’s still, not to get like overly Catholic, I'm sorry.

**Q:** No, that's fine.

**A:** But that's how I have to think about it. That if it does happen, it won't be by my own hand because I've made so many changes, like a total 180 on everything.

**Q:** Thinking about the consequences that can follow a health condition or some sort of issue, do you think that having a silent stroke would put you at risk for something else?

**A:** Yes, it would because it’s happened once. That area could be compromised, it could be weakened. You know, there are blood vessels, though sturdy, if it’s already gone through some sort of trauma, and I also think about the trauma of radiation, even though it’s ten years behind me, it will lend a certain fragility, in my opinion, to like my brain. So, something could happen down the line, something that's not a stroke.

**Q:** What sort of things do you think it would put you at risk for?

**A:** I always think Alzheimer's. I do think Alzheimer's. The radiation damaged a lot of my thinking process initially, and we may have briefly skated over that when I initially met you. But I had to go to cognitive behavioral therapy because I lost my short-term memory. I couldn’t remember, I would like leave the house and be like, “I'm not wearing pants,” and then I'd look down and I have on pants and I wouldn’t remember getting dressed. I would drive somewhere and not know how I got there. And it’s not like it was for a little bit after it, it was for a good seven months.

And my doctor in Wilton, who was the one said, “I can't believe anyone hasn’t MRI’d you,” she thought based on how I was testing out and the damage, she said, “You're probably not going to be able to work again and we're going to have to apply for SSI for you.” And I'm just like, “No, that's not acceptable. What do I have to do to make this be different?” I still have problems. If I'm tired or I'm very stressed, I will have problems speaking. And I know that that's always to be.

There are times where I have little blips where I joke with my sister that, “Yup, there it is. I'm getting Alzheimer's. It’s starting now.” But for me, that's always something in the back of my mind. Knowing what happened with the radiation and how intense it was and what happened to me afterward.

**Q:** Okay. No, that's very good. I'm glad you had the chance to describe that. What I'm going to do next is actually I'm going to list a series of medical conditions or symptoms and the framework for this question is whether or not you think that having a silent stroke would put you at risk for these issues. And you can answer as yes or no or some qualified answer somewhere in between that. And not all the answers are yes or no. So, it doesn't-- there's no right or wrong answers per se, it’s more just getting a sense for how you're thinking about this in terms of its impact on your health.

So again, for which of the following do you think you're at a higher risk because of having this abnormality on the scan? So, the first one is stroke?

**A:** So, yes, I'm-- that could happen again.

**Q:** The second is intracerebral hemorrhage, which is a bleed into the brain tissue?

**A:** Uh-huh. That one I'm not really sure.

**Q:** Okay. The next one is heart attack.

**A:** Yeah. And you're like, “What?” And I say that, though, because it’s kind of not all the way about this. I mean, this connects with my heart because they can see on the tape that I'm pointing at-- I forgot about that, sorry.

**Q:** That’s okay.

**A:** But all of the foods that caused whatever issues, because I was, just as like an aside, because I was a laxative bulimic in the ‘80s, and then I was also a binge eater and as you can tell from my fabulous figure, I still have food issues. I made the worst food choices that you would ever want to make, like horrible, everything with sodiums and fats and bacon and frosting using Keebler Fudge Sticks as a spoon. So, there's a damage that’s already been done, that I could eat healthy for the rest of my life, but it won't necessarily undo that damage. So yes, I'm sure that's another reason why, you know, I'm Fitbitting, I need to get my weight down. I'm working very closely with that. I joined the Y this weekend. So going to rock it out in 2016.

**Q:** Great. How about heart failure?

**A:** Like congestive heart failure?

**Q:** Right?

**A:** I have personally wondered about that. And again, I would say that if that happened to me, it would be more through previous past poor choices than anything with my brain.

**Q:** Sure. Do you think having the abnormality on the scan puts you at high risk of high blood pressure?

**A:** No.

**Q:** How about high cholesterol?

**A:** No.

**Q:** Diabetes?

**A:** Already have it.

**Q:** Headache?

**A:** Yeah.

**Q:** Vertigo, just spinning sensation?

**A:** I do have vertigo. It’s not as bad as it used to be, so it could come back. So that's almost like a wash, the vertigo.

**Q:** Okay. Do you think that silent strokes put you at more risk of that, though, or make it worse?

**A:** No.

**Q:** How about seizures?

**A:** Yes, definitely, definitely, definitely.

**Q:** Okay. How about falls?

**A:** I'm already so uncoordinated, I don’t need any help with that. No, because to me that would link back into a vertigo issue or an equilibrium issue. So, that would be more an inner ear than anything on my brain.

**Q:** Okay. How about memory loss?

**A:** Yes.

**Q:** Dementia?

**A:** Yup.

**Q:** Anxiety?

**A:** Anxiety in what respect, that I'm stressed about having a spot on my brain or that the spot on my brain is a catalyst for some sort of chemical imbalance that would cause it?

**Q:** You can interpret it either way?

**A:** Darn it.

**Q:** Your choice.

**A:** I would say anxiety, just to a certain degree because I would be so focused on the spot that I would psych myself out and anything that happened would be, “It’s the spot,” and I'd get all whipped into a frenzy.

**Q:** Okay. How about depression?

**A:** Possibly, because in the past I suffered from depression. So I was medicated inefficiently at the time, and I'm not on anything now. You don’t have enough recording time for that.

**Q:** Take your time.

**A:** Because of a lot of the circumstances of my family and inter-dynamic family relations and whatever. But, I think that a hormonal imbalance have a lot do with depression, so if anything it would be more related to if the pituitary tumor became active again. And then that, to me, would trigger the ability for depression.

**Q:** Okay. And the last one is schizophrenia? Do you think that abnormality on the scan would put you at risk for that?

**A:** Yes.

**Q:** You do? Okay.

**A:** I do because, again, that's another-- I don't know, for example, what quadrant of the brain or what section of the brain is where schizophrenia would live. Like, if you were going to-- see, I'm losing my words. If you were going to look at someone’s brain and say, “Oh, well, they have brain damage here, and everyone with brain damage here gets schizophrenia,” I don't know what section of the brain is the one that's vulnerable, so I have no idea where that giant spot is. Don’t tell me, oh my God, because I will have schizophrenia as soon as you tell me. Thank you.

**Q:** Okay. So that's it for the list. You've already alluded to this in terms of behavioral change, but what sort of things will you change as a result of learning about the finding on the scan?

**A:** Okay, so I'm very lucky that my doctors here at Tufts are very actively engaged, and it’s not the doctor’s job to babysit a patient, but to just be a resource and give guidance and give support. So, after I left the hospital, like the emergency room, my diet immediately changed, like I legit threw everything out that I had because it was all crap. And I've been food journaling, I've got a Fitbit, I've been doing the 10,000 steps a day except on Sundays, it’s a little hard. I've added in more activity, I've joined the Y, I'm watching my sodium. I'm having some issues testing my blood, which I let Dr. T know because I'm waiting to see the dermatologist because I'm showing you my fingers, I've got some problems with my skin right now. But I have been testing and monitoring if my sugar is high. I look back and see what did I have, what time did I have it?

You know, small changes like that. The changes I've made are all small, doable changes, but I don’t have ridiculous goals of, “I'm going to lose 50 pounds by tomorrow.” I'm not weighing every day because that makes me crazy because I get a little OCD and then I start going back and thinking, “I'm going to have to buy Correctol,” and that's not the way to do it. But, I've been weighing once a month so I've had two weigh-ins so far and I've already lost three pounds as of the pre-Christmas weigh-in, so that was good.

It’s just I can't-- I was going to say a bad word, I'm sorry. I can't mess around with this because I'm 47, 48, I don't know, I'm something, math is not my strong point, and I don’t have a lot of time. You know, it’s not like I have another 60 years ahead of me, I've got another, what, like 30 years ahead of me. So I need to make sure I'm as healthy as possible because the only one I'll hurt is myself if I don’t.

**Q:** Okay. The next part, I'm coming close to the end of the interview, but the next part is a little bit of a series of hypothetical questions. So, these questions have focused mostly on this possibility that this is a stroke that didn't have any symptoms and occurred because of a type of injury that's related to the blood vessels. And so, if I told you that there weren't any current national guidelines or specific research studies that guide physicians in terms of selecting tests or therapies for patients who have a stroke without symptoms, or a silent stroke, is that a fact that would concern you?

**A:** Yeah, that's crazy ridiculous. And I say that because-- I know I mentioned this to you on the phone when you asked me about this-- is ten years ago, I had two different dentists because one, whatever. And I had some teeth pulled one by one dentist, and one by another dentist. And when I went back to the last dentist I had, and it was maybe not even a year, just around a year later, and he said, “I can't believe it doesn't even look like I pulled a tooth. All your teeth have filled in.” And if he had had any training in dental school about acromegaly and how it affects people whose skull plates have already fused together, so not children, because that's one of the things. Everything grows, your nose. I have a giant clown nose now, giant-- you know, whatever. And now that’s something that they do because one of my visits with Dr. S, he said, “I have a dental resident. I've asked her to sit in because I want her to learn about what to look for with acromegaly.” I'm like, thank you. So yes, so the fact that it’s not something that's even on the radar right now, that's horrible.

**Q:** It sounds like something that would concern you if--

**A:** Yes, it is a concern because if they don’t know in ten years like how will they make any changes? How will they be better equipped to help people without studying what's going on in here?

**Q:** Let me ask one more question as a clarification. But, I gave you some advice, but did any other doctors give you advice after our visit?

**A:** Well, Dr. T, and I took like everything except I still can't eat salad because it’s nasty. And I haven't seen Dr. S yet. I'll see him next week. But a lot of what I know he would ask me to do, Dr. T has already covered because the two of them message about me. And the dermatologist is unrelated. So, really it’s you, Dr. T, Dr. S.

**Q:** Does knowing about the absence of guidelines or studies looking into this specific issue, would that change the way you would perceive our advice or the amount of trust that you'd have in that advice? And I'm asking you to sort of give an unbiased answer. It doesn't matter what you tell me for the purposes of the interview.

**A:** I don't think it would because you have to start somewhere. So, my thought is you're basing your own thoughts and your suggestions and sharing with me your perceptions about this on the experiences you yourself have had as a doctor for however long you've been doing this. Whatever journals you're reading, things like that. And again, I say that because I know how much my mother would keep up on things. And sometimes, there would be things in like RN Magazine, or the nursing magazines, that hadn't really been even implemented or thought about yet. And then in like five years, they’re like, “Oh, we've got this new thing to do.” And she's like, “Really?”

So, there's no reason for me to distrust you because you're not making any suggestions maliciously. You're also not overzealous like, “Let’s do this and see how it goes.” You're well informed, and I get the sense that if you truly didn’t know the answer to something, or was unsure that you would say, “You know what, Lisa?” Or, sorry, that's okay, I don't mind. “I really don’t know the answer. Let me check with some colleagues and see what they have to say.” And I would appreciate that. But, because you're being real, you're not trying to bluff your way through it.

So when I moved here, I was going to a doctor in Somerville who-- whatever, my roommates go to her-- and I was having some problems with vertigo again, so I made an appointment. And she said, “Oh yeah, come in. I can help you with the vertigo. There's a procedure to do.” Except she didn't know the procedure and she thought she saw something on “Good Morning, America.” So I sat there while she tried to look for YouTube videos of what she saw and she's like, “Okay, this could work or this could make it worse and I've never done this before.” I'm like, “I'm leaving now, bye.”

So, you know, she instilled no confidence. But, I have that level of trust with you, Dr. T, Dr. S, that if you didn't know the answer, you would whatever. And you're just really using your best judgment. You guys haven't been doing this for ten minutes. If you were to say, “You know what, Lisa? Let’s cut open your brain,” I'd be like, “Yeah, I'm busy that whole year.”

**Q:** Fair enough.

**A:** Because I don't know that, at least right now, that's the right thing for me. But at some point, they should have some sort of research, or look at silent stroke, like are people who pass away, for example, that their doctors suspect or have been able to confirm that they’ve had a silent stroke, do they ever say, “Would you mind donating your brain so we can study it?” Do they do that? I mean--?

**Q:** Not yet, not for that specific question, no. But there are people who have-- who agree to autopsies or post mortems. A lot of things are looked at in that setting.

**A:** Right, because that would be something that-- and I have to redraw-- I have a living will and all this stuff because my mother has passed away and she was on everything. But that is going to be one of the things that I will be including because of how much interest in Connecticut ten years ago, what was going on in my brain was, and they wanted to study me and I'm like, no. But that was a whole different time and a whole different thing.

But, if what I have can help someone learn something to help other people, why not do that? So, hopefully they’ll, you know, they’ll do studies or they’ll do something because I'm sure I'm not the only person with this.

**Q:** So it sounds like it would be important to you that it’s something that's studied more?

**A:** Very much so. Yeah, and especially because my mother had-- it was a TIA, but who knows if she’d had silent strokes before that she wouldn't go in the MRI machine so we never got a scan. And my sister, for some reason, didn't want to do an autopsy. Whatever. So, we’ll never know.

**Q:** In the context of, again, the situation of not having guidelines or studies, if there were studies that showed that specific behavioral changes, for example like changing diets or exercising or specific medications provided some benefit in preventing consequences of silent stroke-- for example, having a stroke with symptoms-- if there was benefit that was found, but they're relatively small benefits or modest benefits, do you think that would still be motivating for you in terms of, “Yeah, I would take this pill,” or, “Yeah, I would make this change to my exercise regimen or my diet or so on?”

**A:** Exercise regimen and diet, yes. But because for so many years, again because I had all these different doctors who didn't talk to each other, I was on too many medicines because one would give me this and I would have side effects. So I'd get something else to counteract that side effect. Because when I moved up here, I had like eight prescriptions for things. So for me to take another pill by mouth-- I mean, obviously I'm taking the Lipitor and the baby aspirin. But for me to take something that would have a TV commercial with people running slow-mo across the sand?

**Q:** Sure.

**A:** There would have to be a lot of benefits because when you hear the disclaimers on all the things that are the side effects, I can't imagine how anyone would ever want to take them because the side effects are out of control. And I know everyone doesn’t get every side effect. But for some minimal benefit, I wouldn't.

**Q:** Okay. That sounds like for something like aspirin and Lipitor, you're okay with that because they're medications that the side effect profile you feel is acceptable and you know that there's benefit.

**A:** Correct.

**Q:** But for other things, you would need some high burden of proof to show that there actually is a big benefit?

**A:** Correct. Like when we talked about at my visit, if I needed a different blood thinner and I said, “Not Xarelto because of what happened with my mother and how we're involved in a Xarelto suit and everything,” and to know the suffering that woman went through when they just could have put her on warfarin. So, I'm very aware of the pros and cons of what you get versus what you end up with.

**Q:** Sure. So we're almost done. There are two more questions.

**A:** All right.

**Q:** One is just reflecting back on this interview. So the process of going through this exploratory exercise, has that actually changed your perception on what you have or the idea of silent strokes?

**A:** No, because again, growing up in kind of a medical environment and we had all this-- the “American Journal of Nursing,” and all those magazines in the house with their creepy, icky pictures, and always being around nurses and doctors, there's really no change for me. There's no perception change because it’s just like another day of whatever. But this has been extremely helpful because it’s allowed me to kind of step back for a minute, especially the question about how did you feel after we said, “Oh, it could be a silent stroke?” And then like, you know, I really had to think for a minute, well how did I feel? So that's good. I mean, I heard it, whatever, and I put it away. But in actuality, it was actually a blessing for me.

**Q:** So, the last thing is so after this I'm going to go through just a very brief debriefing to kind of let you know what the state of the art is and what we know in the field. But I wanted you to have a chance to voice any additional thoughts you have, or additional questions you might have now that we've come to the conclusion of the interview.

**A:** Well, I'll say, which I had mentioned before you put the tape on, the worst way to tell a patient that they have something like a brain tumor is-- and I don't know if it would work this way at Tufts because you're a hospital, so you're always open-- but you know it’s really bad when your endocrinologist who’s been on vacation calls you on a Saturday morning when these no office hours and tells you she has to speak with you right away, it’s an emergency. You need to meet her in her office at whatever time it was. I'm like, “It’s a Saturday.” She goes, “Well, I'm going to be there.” And I'm like, “What's-- you know, is everything okay? Am I going to die?” She's like, “We’ll talk about it there.”

But it was because it turned out she was so freaked out because she had never seen a pituitary tumor that was this large because it was down in my left sinus as well and over the carotid artery and had dented the bone. She had never seen anything go unnoticed for so long, so it freaked her out. And that’s a human response. But when you're a doctor, sometimes you-- and not you, but generically, you got to reel that in because it freaked me the hell out. And I'm like, “I'm not going.” And my mother’s like, “You are getting in that car.” I'm like, “Okay, fine.” And we went and it was very dramatic, like it would have been fabulous in some Bravo reality TV series, the way it all unfolded.

**Q:** Yeah.

**A:** And it struck so much fear into me, like that was it, my life was over, I might as well just go step out in front of a car. And I wasn’t going to have the surgery. And it was very difficult to get the appointment with the neurosurgeon to even see me before I had the surgery because he was the one that everybody wanted and I was in so, so, so much blinding pain from the migraines that I do not remember the name of the drug, but it was actually like an anti-seizure medicine somehow that had some sort of pain killer effect that I took an overdose of it because I couldn’t stand the pain anymore and I got carted off in an ambulance. Which was super embarrassing having to explain that to my landlord. It's like, “Well, I kind of tried to kill myself, but I had a really bad headache.”

So, it’s hard. You know, the brain-- I almost want to say the brain is scarier than the heart because the brain, you're like, will my speech be impacted? Will my sight be impacted? Not that your heart’s not important. So that's why I think when you're dealing with patients and talking to them about issues of the brain, calm. You were awesome, you were like so chill about it. You didn't make a big deal. I mean, it’s important, but you didn’t, like, whip me into an hysterical frenzy.

**Q:** Okay.

**A:** And I loved that so much. And that really was so helpful for me to be able to engage and listen to you except for the part where I blocked out after you said silent stroke. [laughter] But usually, I'm better than that.

So, yeah, it just really-- it’s, I think, possibly bedside manner, even though it's not a bed. But you got to treat the patient and the delivery of the news as delicately as you would treat their brain if you were operating on it.

**Q:** Sure.

**A:** Brain’s scary.

**Q:** I'm glad that you shared that. I'll stop the recording right now.

**A:** Okay.

END OF INTERVIEW

**Participant 4**

**Q:** Okay, so we’ll get started right now. There are about eight questions in terms of the standard questions. So, this is the interview for our fourth patient, so thank you again for joining us. The first question is very broad, but the idea is just for you to tell me just in a very open-ended way what you know about your diagnosis, or what you were told about it?

**A:** Okay. Well, basically from what I understand, I had gone-- my first visit, we were basically following up on an MRI which was originally I got into because of my mom’s history and her mom’s history look at an aneurysm. The other findings, of course, the white matter, just wondering why I was having those changes, I guess a moderate amount is what, I guess, it was said on the first finding. And I think that didn't change much, is what I was hearing on the second. The first one I was told maybe the migraines, that I'd had migraines often when I was younger. On a second visit was thinking at least apparently with the test, it looked like there was more [00:01:19] had seemed to see more in a year’s bit of time. So, we were wondering, you know, if I'm having migraines, still what might be going on.

So, but apparently I guess, too, I guess Dr. [00:01:32] a different machine, a different test and it really wasn’t a big difference in a year, is what my understanding was. I've also been having more episodes of high blood pressure, mostly at work, you know, when I did decide-- actually, I would have never thought of checking them at work until, you know, I'm hearing about my-- you know, about my test and there being a change. And I thought, well maybe I have something going on besides the migraines from years ago that might have caused this. So that's, you know, and at work, it was quite high. So, you know, so I guess like I said, maybe the idea of whether it be silent strokes, things like that, you know, causing this and maybe being preventative, putting on a statin. You know, I already was taking the aspirin the previous year, so basically as I think that's my understanding just to do prevention was the best bet with the statins. Which I will be honest, I actually haven't started taking yet. He prescribed 80 milligrams which I had intended to take. I did have a primary appointment [00:02:40] on that, was well-- she wasn’t-- different calculations everyone uses for the risk factor and she had hers and she said, “Well, I'm not so sure about that right now.”

So I do have a cardiovascular appointment coming up in February this year with her, too. So I'll be honest, I haven't started taking it yet. But yeah, basically I guess just from my understanding, just a little vascular small blood vessels, basically at risk and not-- he didn't seem very concerned or was a great risk, but there was a risk enough to consider being on a statin even with all my, I guess, triglycerides had been normal the past several years. But I guess that's the new protocol on times is preventative with the high dose statin. So that's where I'm at. And just haven't started it yet, so.

**Q:** So you mentioned multiple people, Dr. M, the neurosurgeon, Dr. T a stroke neurologist, your primary doctor. Who was the first person who told you about the findings on the scan?

**A:** Well, the first person who told me about the findings on the scan-- I got a phone call from the first scan, which I guess that was a year and a half ago, from the MRI that I had [00:04:00]. And basically question [00:04:04] aneurysm at that time. And at that time, also-- well, actually I'll say at that time no, they didn't tell me anything about the white matter changes. I didn't know anything about that until I asked for a hard copy myself, which I tend to do when I get obsessed because I like to know what's going on.

**Q:** Of course.

**A:** Because I have found out when I had [00:04:21] replaced that at first I didn't have any real problems. And I found out by reading my own report that I did. More so than I was told. So, you know, because I actually wasn’t told anything about that. And I thought, “Well gee, I was more worried about that than the aneurysm,” because I figured, well, they can keep an eye on that. But what are those white matter-- so it’s like yeah, you do start thinking, like-- and a moderate amount. And I thought, “Well, gees, I'm really young still,” or so I thought, you know, to be having all this, you know, knowing there's various-- as I'm aware-- various possible reasons for white matter just that, you know, you think all kinds of things. You know, you start Googling this, that, whatever. So certain-- so yeah, so basically finding it and reading that myself and started to [00:05:03] about the white matter changes as opposed to the aneurysm which I originally went in to have the MRI for.

**Q:** Okay.

**A:** It was only a screening that my mom talked me into, but I mean, I guess I blame it on the moms. [laughter] Just knowing [00:05:17].

**Q:** When you first broached the subject, or when you started asking doctors about it, how did they describe it to you, or what sort of terms did they use to frame the topic?

**A:** Hmm. Well, really, first doctor I did see about it was Dr. M. You know, for the aneurysm, of course, that was the original reason I-- and he did say, you know, that I should see a neurologist for that. He just did say at that time, basically, you know, there are some white matter changes, I believe there could be various reasons but it would be good to see a neurologist. Have him, you know, look at it more to see what they feel. And Dr. T because, you know, just based on, I guess, my history I hadn't had smoking, high blood pressure, at least hadn't been diagnosed or had-- that I know of at the time or, you know, any unusual risk factors, cholesterol had been fine.

So at that time, you know, again, just saying, “Well, you know,” asking, of course, if I had any symptoms of any-- you know, stroke-type symptoms, any little-- you know, anything that-- over my lifetime but I could recall nothing that I have had. I've had no symptoms. I've had nothing that would lead me to think I had anything like that. So I guess at that time the focus was maybe I had many years of monthly migraines. Maybe. This was my first MRI. Who knows what they were-- would have been if I ever had one in the past. But, I mean, that was the thought.

And again, not having them in many, many years and, you know, I only went back to follow up and question of it being any difference. No, there's not much difference, but I mean basically since I've had high blood pressure, he did say, “Well, this is something certainly to look at, possibly high blood pressure medicine.” He referred me to my primary to see about that. Of course, it was very high in his office, too, as it tends to be sometimes, that white coat syndrome thing. It was not too bad in my own doctor’s office, so that's just a routine exam. I mean, it was higher than it should be.

So, you know, she wasn’t against the high blood pressure medication, but I'm still waiting on that for doctor-- the cardiologist. That's something I probably will end up doing and the statin, I guess, obviously there's always a different opinion. So she wanted me to[00:07:57] I'm waiting on that. I'm a little leery of-- I've made it to 54 without taking anything prescription-wise. So I was hoping to see, well, if they don’t think I should, maybe I'll hold off. I don't know, I'm also trying other things; diets, I get more exercise. I've always done it, but I'm doing more. So yeah, trying all kinds of things. But yeah, basically I think they just-- my understanding from Dr. T was just that-- I mean, I get the feeling it’s-- which I guess is true, we don’t exactly know what the cause is exactly, but it could be this, it could be that. You know, blood pressure could play a factor. You know, all these things. Not sure, basically, based on a history of not having any particular history, any risk factors that we know about.

So, I guess that's often the case. Obviously, don’t always know exactly what, you know, the specific cause might be if there's no definite like, “Oh, you've had this and this and this,” and I hadn't had that. So, I guess I'm just supposed to be trying to treat, you know, prevention-wise, as I understand it, with the statins and aspirin and stuff like that. So, the risk factors that I apparently have, so. I mean, [laughter].

**Q:** That’s very good. You actually answered about four or five of my next questions.

**A:** Oh, try to get the [00:09:17].

**Q:** We can kind of explore it a little bit more, too. I was wondering, so it sounded like you--

**A:** [OVERLAPPING VOICES] I guess I kind of went off. [laughter]

**Q:** It sounded like Dr. T had asked you about maybe some specific stroke symptoms. Was that the first time that the idea of stroke came up in terms of a doctor bringing it up or for you to think about that? Or did it occur before that or after that?

**A:** When I first read the report, obviously that was one of my concerns. Previously, the first year, which a year and a half [00:09:51] now, obviously I hadn't had any symptoms but reading the report, you know, I thought myself, of course, “Wow, this seems like, you know, could be a risk for stroke.” And I was trying to think if there was any symptoms I could recall, blood pressure, at that time was, you know, fine as far as I had known anyway. So I don’t smoke and I hadn't had any risk factors to my knowledge.

So again, you know, you're just kind of thinking of various things. But yeah, that certainly, in my mind, came up back then and you start trying to do better with diet and this and that and all that stuff and more exercise and that kind of thing. So, thinking, you know, want to do everything you can to prevent stroke being as I see plenty of it at my age, people coming in. So yeah, especially when you see it and, you know, you're around it and you think more about that, even when I first got the report, I read it so-- before anybody brought it up, I'm already reading it myself and think, I got to be careful about this and this, yeah.

**Q:** We're very interested in terms of the words that people use. So I'm actually going to read out a few names that people have given for these. So, sometimes if people-- if the doctors will come to a more specific diagnosis, they’ll call this a silent stroke, a silent brain infarction, a silent cerebral infarction. Some people call it a covert stroke, a covert brain infarction, a sub clinical stroke, subtle stroke or asymptomatic stroke. There is a gray zone, but did any of these terms ever come up before I spoke to you about this study?

**A:** Well, I thought silent stroke was mentioned as a possibility of what could have happened. But again, my understanding, you know, also not knowing, not being sure, obviously, and not me having symptoms that I can recall. But I believe that's the only term that I heard mentioned regarding any possible strokes, just based on what they were looking at, as I recall.

**Q:** Out of curiosity, do you remember which doctor used that term or which doctors?

**A:** It would be Dr. T I thought, yeah. Dr. M, again Dr. M, he mostly referred-- he just sort of was dealing with the aneurysm risk and the angio and all that, just confirmed aneurysm and all that. But referred me to Dr. Thaler [00:12:25].

**Q:** Did anybody ever describe findings as being incidental or unexpected?

**A:** Well, I think Dr. T mentioned this is, you know, that came up, he mentioned this can be fairly common. People a lot of times-- I mean, if I hadn't been strings [?], you know, I mean if this is what people usually obviously [00:12:55] and aware, you don’t know you have any particular thing going on. It's when most of these things come up and looking for one thing and people find the other things, and then you start questioning that because, you know, obviously it’s not what I went in there for. So it’s one of those-- yeah, I think it’s a finding brought up because of the fact that it’s not why I was going there to check on an aneurysm and all of a sudden everything else comes up about well, why all these white matter changes and that. So, yeah, that was brought up, I believe, yeah.

**Q:** My next question was about the advice given to you by your doctors. And I think you mentioned some of them already and some of the conflicting recommendations. So I know you mentioned the statin, which it sounded like Dr. T had recommended that you take a cholesterol medication. That your primary doctor had performed a calculation of your risk and thought that it wasn’t quite necessary. So it sounds like you're going to talk to your cardiologist about that as-- to see whether [OVERLAPPING VOICES].

**A:** Yeah, which I haven't-- the GP referred me to and just thought, you know, if we're unsure, and she was unsure. And obviously I'm unsure so I hadn't started taking it which I had planned to when I left there. But, you know, not having had such a medication, of any significance anyway, you know, that I've had to use yet, I just was hesitant. So I was like well, I just want to wait. And Dr. T even said, you know, he certainly could wait and discuss it with the primary. So, I had wanted to wait at the time, I ended up waiting. So yeah, I mean, yeah, there is-- and that's understandable. There always is different thoughts. But, a medicine, that's how it is. So I'm kind of weighing it all out now with everybody’s thoughts.

**Q:** Right.

**A:** Whether I should or whether I shouldn't. I know that's-- and I wish actually-- I don't know. I meant to ask more. I guess I wanted to know more about-- and I still want to know more about-- the preventative-- how the 80 milligrams of the high dose helps and I guess there was a study regarding that, my understanding, because if people-- other people are on it, there are many patients on it for preventative, many that actually have already had, you know, stroke incidents.

But I mean, even if your labs, your cholesterol, your triglycerides, all that is normal and I hadn't asked further about that, actually, and I wished I had. But, why is it that it really-- I guess I just find myself questioning more why, how it works that way. Even if your lab’s always been fine, that's-- I'm trying to wrap my head around something. Well, my lab, all this stuff is fine. You know, do I really need that much? And I guess I just am trying to-- I have different thoughts on it, I know and it’s certainly not uncommon to use the high dose, as I understand, and I see myself. But I'm just not-- wasn't quite sure about it, before I get into, you know, taking a-- that dose, but yeah.

**Q:** Right. I could tell you my thoughts about it afterwards and what I know about it and all we've talked about.

**A:** Thoughts, yeah.

**Q:** Besides statin, were there other specific medications or lifestyle modifications that your doctors suggested?

**A:** Well, Dr. T was the first one, he suggested the aspirin when I went the first year, which I didn’t start taking that-- well, everybody over 50’s on an aspirin, so I figured why not. Might as well [00:16:32] so I did, you know, did do that. And not a lot was mentioned, I think, of other things. I actually said on my own, I am trying to-- being as I understand a fair amount about this, healthy, I'm trying to, you know, certainly better diet, eat more vegetables and all the healthy stuff, trying to do more exercise. So I said it on my own, I don't think anyone had actually suggested it to me because I was already saying that's what I'm trying to do. But I assume if I hadn't already said I was doing this, they’d be telling me that's probably a good idea.

**Q:** Right. You mentioned that Dr. T had acknowledged a degree of uncertainty about what to do in this type of situation. Or it sounded like that's what you were describing?

**A:** Well, not so much-- no, I think he was-- thought it was a good idea, it’s pretty common to use a statin. But not what to do so much as-- which is understandable. Not exactly sure, you know, what exactly might be causing it, you know, which is the case, often is the case, you know. Because me not having any symptoms or any risk factors that we know about, you know, I think it’s still kind of-- at least I understood it, it was not definite what was the cause. Not so much what to do about it. I think he felt like it was a good idea to do the statin, which I'm not saying it’s not. I'm not sure that it’s not. I'm still kind of just laying it all out with my doctor telling me, “Well, hold off.” Which is often the case. Sometimes there's different opinions and then I'm kind of like just trying to figure out-- I just got to take them all out and see, put them all together and decide what to do.

**Q:** Your primary doctor had a conflicting opinion about the statin. Did your primary doctor seem certain about that recommendation in terms of what to do?

**A:** Yeah, she was very surprised on that much based on her calculations. So, I wouldn't say slightly surprised, she was very surprised. So I have differing, now, opinions from my primary and neurologist. So that's kind of like-- she was even close to thinking, well, I'm going to go with-- go ahead and start. But she was very much to the other-- she gave me, I think, a 1.6 or whatever on her calculation. So, yeah, kind of-- so now I'm kind of well, which way to lean, you know? Like not sure which one-- well, but at this point I was actually leaning towards not taking the medication because I'd rather not, of course, even though-- basically, I guess the cardiologist will hopefully break the tie here, decide what I'm doing from there. I guess. You know, it’s-- it’s hard and obviously everyone’s going to have different opinions. But, you know, as being a patient, you're like, “Well, who do I listen to?”

**Q:** Right.

**A:** So, that is-- I mean, I've known her for many years, actually this is-- but then again, too, you know, I go to Tufts, I bring my mom to Tufts. I have a lot of faith in everybody there and obviously a specialty and everything. So, I'm kind of not still sure yet, though, of what I want to do. So, you know, so actually hearing your opinion will be helpful.

**Q:** Sure.

**A:** I'm looking to get all the info I can on it, actually.

**Q:** You mentioned even at the very beginning when you had the first scan, reading the report and being worried about the risk of having a stroke based on how you're interpreting the text of the reports. Is that the main concern that you have, or are there other concerns that you have as a result of finding out about this?

**A:** No, that was the main thing, actually. Is my mom obviously had it when hers related to a ruptured aneurysm, obviously. You know, and so when you see that, when you deal with that and you are [00:20:57] someone with that, aside from seeing it at work and then you read your own report and you're like, you know, you worry about what's my situation. So, yeah. With the aneurysm-- again, all I was told over the phone when I first got the report verbally was, “Well, you know, whether it be there's a formation that [00:21:17] vessel, it may be formed, or aneurysm,” they weren't sure and that's where the testing came up. Nothing was mentioned about a [00:21:24] to me originally because as often is the case-- not often is the case, but sometimes the case-- where the person-- I don’t want to say it was the nurse telling me the report. When I called my doctor, it was kind of like that wasn't the primary reason I was going for the MRI, the other wasn't mentioned because it’s-- and I think the words in there were, you know, chronic on the report, chronic [00:21:50] ventricular [00:21:52] so it was kind of like, well, that's-- chronic and that's there and nothing was mentioned about that. So I'm reading it and to find it out it’s like, it’s [00:22:01]. Sort of thing. [laughter] So I'm not sure.

**Q:** If you were to somehow quantify or estimate the degree of concern that you had after finding out About this, how would you say-- how concerned would you say you were?

**A:** On a scale of one to five or something like that or something?

**Q:** Sure?

**A:** Maybe like a five being greater concern? Yeah, probably had at least a three, probably, concerning. It was concerning. You know, it’s on-- again, dealing with, you know, my mom going through that. So, and reading my own report, you know, which is sometimes good, sometimes bad. Sometimes it makes things worse. If I never had a scan, I'd probably be like, well. So, who knows? You weigh those things out. But, again, then it’s probably good you do more and I think I'm probably leaning towards that, that it’s-- incidentally-- incidental finding, which is good for me to know so maybe you do take things more serious as far as like prevention, you know. So I suppose it’s good, so. [laughter] Oh yeah, concerned, I would say.

**Q:** Okay. In sort of a broader sense, in terms of when you learned about the diagnosis, how did it make you feel?

**A:** [laughter] Well, old comes to mind. Oh, I'd just say, you know, I was just like, Jesus. I mean, I think it-- I actually had two hips replaced already which, you know, in my 40s, my early 40s. So, I felt way too young for that. And I was okay, that went well and now I get to 54 pretty quick, 53 the first time. I'm like gees, now something else. So then, I start thinking, gees, I'm getting old. You know, over 50, like now this. So, it was probably my first thought, actually. So, and then just what to do about it. Just trying to, like, you know, moving into more healthy things there. Like I said, five servings of vegetables and flax seed and all this, and whole grains and trying to do more of all the things you should do. Again, be careful about all these things. So, yeah.

**Q:** I think you sort of answered my next question already, which was--

**A:** Sorry. [laughter]

**Q:** No, it’s great. It’s always nicer for things to come out more fluidly and more naturally. So some of these questions might sound a little bit repetitive. But the next question was thinking about whether or not you think having these findings puts your health at risk? And it sounds like you already think that to some degree it does. And it sounds like you think this might put you at a risk of having a stroke. Is that fair to say? Am I summarizing that correctly?

**A:** Yeah. I'd probably say that because probably-- yeah, you know, if I hadn't had the test, I probably wouldn't really think-- because, you know, not-- I mean, I've always tried to at least exercise, I do more probably [00:25:17] and try to do better. I didn't really think I was probably someone really at risk based on never smoked and that kind of stuff there and not had blood pressure [00:25:29] and that kind of stuff. So really probably hadn't-- wasn’t really on my radar speed until obviously this thing popped up, so yeah.

**Q:** I'm going to ask you actually about some specific medical conditions or symptoms. And the framing of the question is to see if you think that having these findings on the brain scans puts you at higher risk for these. And you can say yes, no or give a more qualified answer. And just for your reference, the answers aren't all yes and they aren't all no. It's more just to get you to think a little bit about it and see if any of these-- if you connect any of these to having these findings on the scans.

So again, the question is which of the following do you think you're at higher risk for now that you have these findings on the brain scans? So the first one is stroke?

**A:** Well, yeah. I think I'm at higher risk for that.

**Q:** Okay, the second is intracerebral hemorrhage, so a bleed into the brain?

**A:** Yeah. I mean, I do think I'm maybe at higher risk than the average person not knowing-- even though they didn't determine it was an aneurysm. I know I do have some unusual vascular makeup and my mom’s history, I'm still not convinced I'm not at higher risk than normal. So based on what I know, yeah probably more than the average person probably anyway.

**Q:** How about heart attacks?

**A:** Well, yeah. I suppose I, you know, I do think maybe somewhat. It’s not a big concern of mine. You know, again, trying to exercise, that kind of stuff. But with some of the blood pressures I've had at work that get really high, the few times I've checked at work, you know, it’s something to think about, but it’s not something I've really given much thought as opposed to other.

**Q:** How about heart failure?

**A:** No, it really hasn’t been on my radar.

**Q:** High blood pressure?

**A:** Well, I know-- I mean, being as I'm at work most of the time and when I've checked my blood pressure at work it was 140, 50 over 100 often enough. And yeah, I do worry about that because I'm at-- if I wasn't working, I'd be a-okay. Many of us would. [laughter]

**Q:** How about high--

**A:** My lottery number or something.

**Q:** Right.

**A:** [00:28:14]

**Q:** If only.

**A:** I'll get a ticket.

**Q:** How about high cholesterol?

**A:** Yeah. I mean, I do worry about that. My dad’s high cholesterol, even though he was a health fantastic, he was a runner and he ate everything perfect, but he had high cholesterol. He would never take medication for it, but he ran in the 300s. Yeah, but he was followed for that and would never take medication for it. And maybe some of that's in my head from, you know, from him as well. So, he had various thoughts on it and read many books on it and all that. So, of course that was many years ago, so you know. But yeah, that is something that's in my mind, is the family history, his [00:29:00].

**Q:** How about diabetes?

**A:** No, I really haven't worried about that. I mean, my grandmother had it, but like I said, try to do well with diet and exercise. It really hasn’t been something that I'm worried about.

**Q:** And how about headaches?

**A:** I'm sorry, what did you say?

**Q:** How about headaches? Do you think you're at more risk for headaches now?

**A:** I really haven't given it that much thought. Like I said, I used to get migraines, but those are in my-- mostly around monthly cycle time and that was in my 20s, 30s. But I only had a problem with headaches-- it’s been very rare I've had a headache in the past something years. It's a rare occasion, so I really don’t worry about that much right now.

**Q:** How about vertigo, so that's when there's a spinning sensation?

**A:** You know, I have had-- you know, rare-- not a lot. And that's why actually I don’t think I actually mentioned I even had it. I went in because, you know, when you say it, I have had episodes where sometimes your-- I get up and I've felt a little bit lightheaded. Or sometimes even just occasionally-- and again, very rare, so it’s not that I really even think about it. But now that you mentioned it, I'm thinking, well, a couple of times just standing somewhere and just-- it’s such a-- one of those things that's so slight that I kind of like, ah, you know, not enough to kind of-- when you notice it kind of thing. But it’s been rare, so I really haven't given it a lot of thought. As you say it, as we're talking about it, you know. But again, a rare thing, not something I worry too much about. But I've had a few occasions of that.

**Q:** Do you think you're at more risk of having seizures?

**A:** I haven't given that a thought, actually. My mom, being again had that situation, I probably am but I actually haven't really given that much thought, actually, but probably.

**Q:** Okay. Do you think you're at risk for having falls?

**A:** Not right now, it’s not something I've really given any thought to. No, I really haven't had any problems with that, balance, no balance issues or anything like that. You know, I don't think so, no.

**Q:** Do you think you're at more risk of having memory loss?

**A:** Yeah, but that's come to mind as some people, you know, and every time you forget something that you just went into a room for, everyone-- I'm sure we all do. [laughter] But I remind myself that-- it’s not so much forgetting where you put your keys or forgetting what the keys are for, it’s like I tell myself, okay, it’s okay, we all forget where our keys are sometimes. But, yeah, actually-- I remind myself-- actually, compared to-- I actually have a pretty good memory for a lot of details. I do detail oriented work nursing. I do MDS, lot of paperwork. So I remind myself my memory’s usually pretty good, actually. I remember things that come up in conversation from months ago. I do-- my memory’s pretty good, but like everybody else even without scans or-- I do sometimes, you know, I forget this. I just knew it and I just walked in the room and I forgot what I was-- I've had that happen, but I know-- I tell myself that's not that crazy in this fast paced-- that's what I remind myself anyway, so. But yeah, I worry about it occasionally.

**Q:** Taking it a little bit further, do you think this puts you at any higher risk for dementia?

**A:** Yeah, that's come to mind, too, yeah. Basically working-- my office is like on a dementia unit, so. I see that all the time, so yeah I worry about that.

**Q:** How about higher risk for anxiety?

**A:** Yeah, well probably. It's caused a little anxiety, so I try to not worry about it too much. So I [00:33:37] that medication either, so. But yeah, certainly it has caused some anxiety, so I would imagine that's certainly a possibility.

**Q:** How about depression?

**A:** Well, certainly, you know, could be. If I had any more problems I would know, but I haven't thought about that much so far.

**Q:** Okay. And the last one is do you think it puts you at higher risk for schizophrenia?

**A:** Haven't really even thought about that one. Thanks for bringing it up. No, I'm just kidding. [laughter] Yeah, I hadn't thought about it so I'm going to say no.

**Q:** Okay. The next question’s a little bit of a thought experiment in some ways. But, if I told you that currently there are no national guidelines or specific clinical trials or studies that guide physicians in terms of selecting tests or therapies for silent or covert strokes, how concerned would you be about that in terms of learning that fact?

**A:** Probably not any more concerned. I mean, understanding a lot of things are trial and error, basically. A lot of things are learning and tests and studies like this. So, yeah, I would certainly get that a lot of things are just seeing what works best and based on information that you have. So yeah, there's no set hard guidelines that-- I get that that’s often the case, so.

**Q:** Would learning that affect how you perceive the advice of your doctors?

**A:** Well, probably-- I kind of already-- there are [00:35:47] thoughts from everyone I've talked to, so we’ll see what the cardiologist says. So, somewhat maybe, but again I'm just going to-- like everybody has to do, just kind of put all the thoughts together and make the best educated decision that I can based on, you know, what everyone presents me. I'll just kind of make the best decision I can with what I'm told by-- weigh it all out. So that's just how it is sometimes, you know?

**Q:** Okay. So you've clearly thought a lot about this already. But we're actually coming close to the end of the interview right now, and I wanted to know if your perception on this issue has changed at all even just walking through these questions and thinking about it this morning?

**A:** A little, but most of it, again, I've already kind of thought about. So, but maybe a little bit. I mean, brought up some situations I hadn't actually thought about. Not sure I want to think about. But, no, it’s still good to at least hear any information you can so another reason I figured I'd want to do this because whatever’s helpful in making me-- helping me decide what I do want to do is [00:37:17]. But so maybe a little bit but not, you know, not a lot.

**Q:** The last question I'll just record this part of it and then I'll answer your questions to the best of my ability, or sort of express my thoughts to you and what I know, and go through the debriefing. But just the final question is what, for you, just to voice what questions you have about this issue or what concerns or additional thoughts you have?

**A:** I guess my only-- really, my only thought and concern right now is, you know, what is the best thing to do. And I'm kind of, as I said, kind of in that limbo with differing opinions as to what I definitely will do. You know, so that's really, again, aside from that, I'm just trying to do better with healthiest I can be with exercise and diet and that kind of stuff to, you know, do my own prevention the best I can. But yeah, no, that's really my only-- my main concern now on that. I think I kind of just want to know, you know, what is the best thing to do regarding medications and the best prevention.

So, I can't think of anything. That’s really kind of where I'm at, it’s what more to do aside from what I'm doing. I mean, all of the non-medicinal type things there, it was kind of basic, as everyone knows you don't always do. When you find out something, you know, well maybe makes you want to do it and [00:39:02] done it but now I find I'm doing three to four days a week, now I'm doing five, six, you know, trying to do better with my diet and all that kind of stuff. So that's all on me, you know, doing the best I can with that. I just want to know what more I can do like medically, what I should do. Again, that's something I'm going to have to-- that's my concern, is the medication. Which [00:39:29] and what do I listen to and what do I end up doing. So that’s kind of just where I'm at, basically, that's really about it.

**Q:** Yeah.

**A:** That's where I am right now [?]. [00:39:38] there for a while.

**Q:** Okay. Well, let me stop the recording and then we can go through-- I can kind of let you know my thoughts and we can do the debriefing as well.

END OF INTERVIEW

**Participant 5**

#### Q: So let's start the recording right now. Can you say hello? A: Hello?

#### Q: So this is our interview with actually our fifth patient in the study. And so I'm going to ask you a number of questions. There are about eight questions, but there are a few sub-questions as well, just depending on what's on your mind and sort of what things, what questions you have or ideas that you have. But they're very open-ended and it's really just an opportunity for you to express what you've been thinking about.

#### So the first question is: tell me what you know about your diagnosis in terms of what we talked about in the clinic.

#### A: I know very little about the diagnosis. I kind of wanted to have some type of exam regarding the– because I have had some– with the arteries[?] I've have had in the lower part of my body. So everyone thought– so I'm always open to learn as much as I can. And it helps me function a lot better.

#### Q: So can you tell me briefly about what occurred that resulted in you getting a scan of your head? What were the circumstances? A: Well, I had lost a lot of weight. Probably not eating and very bad habits. A combination of probably getting dental[?] And just not eating. And it was too much weight. So I had gone to the hospital to get that checked and get a series of checks. In the process, of course, of my blood pressure, which was high, and I was just taking that. And so, actually I don't know what happened after that. I just went to work and I was sitting. My coworkers will tell me that I had kind of been non-functional for about ten minutes and non-responsive.

#### And when I kind of became aware I was looking at the ambulance coming in the building, and I assumed they were coming for someone else. And of course, it was me. So for about five or ten minutes, I'm not really sure what occurred.

#### So the hospital took over from there. And it was a combination of things that they were trying to study, which was the weight loss, the cause of that, and also why the loss of conscious awareness. And that's why now I'm going through, of course, a series of different exams, just to rule out any possibilities.

#### And the Tufts Medical Center, they had recommended a couple of things in combination with Boston Medical, which I'm a part of. And one of them was, of course, to see the neurologist and also the hematologist, which I have done that. So the neurologist is where I am right at this point.

#### Q: I'll just clarify, too, that I'll ask you questions that I know the answer to, but for the sake of the interview we can pretend that I don't know the answer to those questions, so that you can express your thoughts.

#### So you went to that evaluation after you passed out and at some point had a CT scan of your head, which showed a small kind of old stroke or silent stroke. And when was the first time you heard about that information from the CT scan? A: That's the first time that I'd heard about it, regarding the possibility of a stroke. The principal concern at the time, I guess, was the treatment of the pressure. Pretty much that's what I had going to the doctor's for, was just to get the pressure the normal stage.

#### Q: So was the finding of that little stroke, did somebody tell you about that in the emergency room or is that something that happened later? A: No, that's something they told me about later, that there was that possibility, that I'd had the stroke, based on the findings that they had seen in the exam. So that's the first that I had become aware that that existed.

#### Q: Who told you that information? A: It was one of the doctors. I'm really not sure what doctor did mention that to me. I think I was more concerned, relieved that they had told me everything was okay and that regard, but that I had had the, at some point had that occur. So to me, I was more or less concentrated on just being relieved with the fact that they had ruled out any possibility that I'd had a possibility of a stroke at the time. They did mention that it occurred in the past. So I kind of left it there.

#### Q: So let me make sure I understand you. You're saying that when you were told about this little stroke on the CT scan, you were reassured that this is something that appeared to be old and something that had occurred a long time ago, and that you were reassured that this was not the cause of your symptoms that caused you to pass out? Is that what you're saying, or are you saying something else? A: No. My area at the time was the weight loss. And so, that had been the main attention that I was given. I was more concerned with that. That it had no bearings on that. I was just trying to correct that. To make sure that that was anything that would hamper or block the progress of trying to correct the weight loss. And being able to function. So I kind of just left that to the doctors and just kind of was open to any other information that I could get.

#### Q: Can I ask you one more time, what was the information that was reassuring to you? A: That it was an old occurrence, by what they could see. And that it was okay.

#### Q: Sounds good. So there are a number of ways that people can describe what happened, like an old stroke or a silent stroke on a scan, and sometimes people use a bunch of different names. Sometimes they'll say it's a silent stroke, or a silent brain infarction, a silent cerebral infarction, a covert stroke, a covert brain infarction, a subclinical stroke, a subtle stroke, and asymptomatic stroke. Did anybody use any of those terms from what you can recall?

#### A: I'm not aware of it, I don't think it had.

#### Q: Do you remember, you mentioned you weren't sure which doctor told you about it, but do you remember what type of doctor told you about it? A: No. I'm not sure. When I was in Tufts, I was really, was meeting new physicians as they were coming by the bedside. And they were introducing themselves, but I don't think I retained very much of a remembrance.

#### Q: Okay, that's fine.

#### A: At the time, there was a lot going on. Still is in terms of all the procedures that I've so far gone through. When I left here, of course I went back to Boston Medical. They continued in their studies, which was just a drastic weight loss.

#### Q: Did anybody when describing this to you ever use the terms incidental or unexpected to describe what was seen on the scan? A: No.

#### Q: Did your doctors give you any particular advice in relation to what was seen on the CT scan? A: No, actually. We just didn't stop there. It was a results of gathering of information again. So they may have, but we didn't concentrate on there. They had recommended seeing a specialist, a neurologist, of course, and the other procedure was the blood, the hematologist. Some other recommendations which they I guess sent to Boston Medical.

#### Q: Did the neurologists recommend anything after you saw them?

#### A: No. Well, the first time that I saw the neurologist was, the day that I was in her office, and that is yet to be continued [10:18] I'm not sure when it is. But I'm not sure what recommendations will come out of that.

#### Q: I can remind you after this, we can look into the calendar. What are your concerns about having had a silent stroke in the past? A: It makes me aware that it does exist. And so to get any information I can to try to reduce the possibilities, I'm always open for any type of information or anything that I can do to correct or whatever I can do to try to make sure that I have an option, a better option of it not happening again.

#### Q: Do you think that having had a silent stroke is a serious problem? Is this a serious health condition? A: No, I think it is, I think it's something you take very serious. But it's something that I just have a way of saying, you have to continue on functioning whatever you do. So that's sort of my main focus. As long as I have the ability to continue, that I will do so. At this point, I am really interested in gathering as much information as I can.

#### Q: How concerned about your health are you after learning about having had a prior silent stroke? Like a little concerned? A bit concerned? Very concerned?

#### A: I'm not concerned. I have to go back to the basic – get up and take care of your body and yourself. You eat right and try to maintain good health habits daily. So that's what I'm probably more concerned with in my approach to it. It would be trying to, again, like I say, gather as much information as I can and try to go in that direction.

#### Q: Just to get you to explore it a little bit more, what sort of things are you concerned about? A: I'm more or less concerned more or less about getting up, eating properly, exercising and just going by daily life.

#### Q: So you're concerned about what should you be doing to improve your health? A: Always.

#### Q: Do you think that the silent stroke was connected in some way to any of your other medical conditions? A: No, I don't try to put a label on it. I will try to gather any information I can. And then go from there. What has occurred, it just happened, and it's awakening in a lot of ways, because I've seen the results of strokes and what they can do. And I'm still functional, so that gives me the opportunity to at least try to do something to prevent that.

#### Q: How did learning about this make you feel? A: Well, it made me consciously aware. And seeing it again, then it reminded me that I could be a part of maybe less of a chance of having another stroke if I learned as much as I could about it and work that way.

#### Q: So you feel that having had a silent stroke, that you're worried about having a stroke that causes problems, disability or symptoms? A: Oh, yes, yes. I wouldn't be aware that that could occur.

#### Q: Do you think that it might put you at risk for anything else besides a stroke? A: Well, I've seen the result of stroke, a minor stroke. And I've seen the procedures or the outcome of the mini strokes and what they, what happened to the individual. Went from a small stroke to their speech, their thoughts or their attitude. And it got larger and magnified itself as time went on simply because it was new to that person and they didn't, I guess obviously, know how to handle it. So it just got larger in a lot of ways, going back and forth, or therapy, different things that that person went through.

#### Q: Let me just ask one additional question. You mentioned that stroke might be able to affect somebody's communication, their talking–

#### A: Communication, attitude.

#### Q: Attitude.

#### A: Right. Physical ability. In that area. The person sort of kind of lost interest in a lot of different things after that, and over a period of time they didn't want to exercise. They just felt that whatever happened happened. That's not my belief; I'm always believing that you should scratch for every else of life you have.

#### Q: Definitely. So I'm actually going to expand on that a little bit. I'm going to mention a number of different medical conditions or diseases and a number of symptoms, and see if you think that a silent stroke puts you at higher risk for any of these. So that's the framework. I'm going to ask you: for which of the following do you think you're at a higher risk because of having had a small, silent stroke in the past. And you can yes or no or give some other type of answer if it's not straightforward. Does that make sense? A: Sure.

#### Q: So the first one is stroke. Do you think having a silent stroke puts you at risk for a symptomatic stroke?

#### A: Well, not having that information, I would say yes.

#### Q: Just your best guess, how you would think about this. Does having a silent stroke put you at risk for having a bleed in the head? A: I would say yes.

#### Q: Does having a silent stroke put you at a risk of having a heart attack?

#### A: Again, I would say yes.

#### Q: Does having a silent stroke put you at risk of having what's called heart failure, where the heart's not pumping as well? A: Again, I would say yes.

#### Q: Does having a silent stroke put you at risk for having high blood pressure? A: Yes.

#### Q: How about high cholesterol? A: I would say no in that regard that that wouldn't be a factor.

#### Q: How about diabetes, which is high blood sugar? A: I would say yes.

#### Q: Does having a silent stroke put you at risk for having headaches? A: Yes.

#### Q: Does having a silent stroke put you at risk for having vertigo, which is a sort of spinning sensation? A: Yes.

#### Q: Does having a silent stroke put you at risk for having seizures, which is when the electrical activity of the brain is not working well. Sometimes people stiffen up and shake.

#### A: Yes.

#### Q: Does having a silent stroke put you at risk for having falls? A: Yes.

#### Q: How about memory loss? A: Of course, yes.

#### Q: Dementia? A: Again, yes.

#### Q: How about anxiety? A: Yes.

#### Q: Depression? A: Definitely yes.

#### Q: And the last one is schizophrenia.

#### A: Yes, possible, yes.

#### Q: We talked about you having had a silent stroke or a stroke on your scan that didn't cause any symptoms in the past. Do you think that you'll change anything at this time now that you know you have this? A: Well, I'll be aware and I'll learn as much as I can based on that and try to do whatever is recommended. I think I'll ask more questions regarding this and gather more information.

#### Q: Sort of engage a bit more and try to understand how this happened or what to do?

#### A: Yes.

#### Q: Have you made any lifestyle changes or sort of changed the way that you live? A: No, well, not really in terms of– my whole life consists of getting up, going to work two parts of the day, and the other going home and just resting. And the weekends I kind of have one routine. I get up and drive. We go food shopping. And I take the little grandson out. And that's my life, and so. I wish I had more time, devote more time to that. But otherwise [21:03]

#### Q: So I'm going to give you some hypotheticals. And I'll tell you afterwards, kind of confirm whether or not these are actually the case. So oftentimes when we're providing care for patients, we follow guidelines. So we look at all the best studies that we had in the past and then a panel of experts will come up with a set of general sort of guidelines or rules that most people can follow for most patients in terms of what's the best care that we can provide. If I told you that there currently are no national guidelines or no specific studies to help physicians in terms of figuring out the best treatments for silent strokes, how concerned would you be about that?

#### A: I would accept it as a fact. If they haven't yet determined what is available, then I would accept it as a fact, that something has to occur, that maybe some day there will be a breakthrough.

#### Q: Does learning about there being no guidelines, does that make you more or less concerned about having had a silent stroke?

#### A: No, it just makes me more anxious to learn more in terms of what I can do to just try to help the situation.

#### Q: Does knowing that there aren't any guidelines or any studies in terms of prevention treatments affect how you perceive or trust the advice of your doctors about this?

#### A: No, that's kind of being honest. If I got that information and it's a fact, then I would have to accept it and it wouldn't change how I feel about living.

#### Q: How important is it to you– certainly there are a lot of things that would keep you busy in any given day, but this particular topic, how important is it to you that it be studied more, that people look into it more? A: I think there's room for that to happen and I think it would be beneficial for a lot of people if there was a breakthrough. That would be very helpful.

#### Q: Let's say that some studies are done to help work on prevention treatments. And as you kind of guessed, you're worried that having a silent stroke might mean that asymptomatic stroke could occur in the future. So let's say we did some studies and found out that making some lifestyle changes or maybe taking some medications might have a little bit of a benefit, sort of like a small benefit towards preventing stroke, do you think that you would be motivated enough by those types of studies to kind of change what you're doing, whether it's exercising more or taking another pill or changing your diet? Do you think that that would be enough to motivate you? A: Well, it would be enough to motivate, but I have to accept things as they are presented to analyze if that would be something that I would, could do. And I would certainly consider all possibilities.

#### Q: When we try and figure out what's the best thing to do in terms of the best way of advising people or best way of preventing health problems, we have to set up outcomes. So, set up things that we look at later on to see can we prevent those things from happening. So in terms of outcomes, what do you think would be the most important ones for us to look in to? So let's say we know that a large number of people have silent strokes. What sort of things should we be trying to prevent? A: I suppose any type of scans of the activity area, that machine that you have, procedure that you have, I think that something could be structured so that periodically at a given time people are tested for the possibilities that something may be occurring. I think that's the only possible way that you could determine if something like that would be occurring, is an exam based on the best knowledge that you have.

#### Q: Let me see if I understand what you're saying. Are you suggesting that because silent strokes don't occur with symptoms, that people should be screened? People should periodically have a test to look and see if they're having silent strokes? A: Well, that would be one answer. I think the other is making them aware of certain things that they could do. And so it's like taking certification or recertification that makes you, of course, aware. And then it gives you an opportunity to follow the guidelines and reduce the percentage that may happen.

#### Q: It sounded like you were suggesting– correct me if I'm wrong, but it sounded like you were suggesting that we should be looking to prevent or at least sort of see if silent strokes are occurring over time. So do you think it's important to prevent silent strokes from happening? A: Well, yeah, of course, I would always believe that that would be the correct thing to do. Again, it's just how do you approach the learning curve. Sometimes you can say something but then if you're not really sure of the person, they don't focus on what you're saying a lot of times, it doesn't penetrate their conscious level enough to take those steps or to function properly. I don't know what that could be, but again, it's like some way that when people are tested or people are seeing their physician that they can also go through some type of mini procedure. That would make them aware, of course, that the possibility exists.

#### Q: So we're actually almost done with the interview. After talking about this for the past about half-hour, has your impression or understanding of silent strokes changed? A: No, it just adds to the information that I would like to gather regarding the physical health. And so, it's just adding to the things that I would like to know.

#### Q: Do you have any other particular questions or thoughts about this? A: No. Only just the medication. What would that be. What type of activity would be beneficial. Those kind of areas, that's really what I would be interested in.

#### Q: Okay, great. Thanks so much for your time. I'm going to stop the recording right now.

#### END INTERVIEW

**Participant 6**

#### Q: So this is an interview with our sixth patient. Can you say hello?

#### A: Hello, good morning.

#### Q: Wonderful, thank you. So I'm going to ask you about eight questions, and there might be a few side questions connected to this. And you could just answer them to the best of your ability or you can answer them with as much detail as you'd like. But I might ask you to sort of explore it a little bit more.

#### So the first question is just very open-ended, but tell me what you know about stroke or about sort of a silent stroke.

#### A: Usually what I know about stroke usually the people that I know that they had it, when they usually paralyzed. Somehow we never talk about anything anymore. Just the people that I have seen, usually they don't have mobility on one side of the body. And it's frightening.

#### Q: How did you learn about that? A: Usually from friends. If someone has had a stroke, somebody will call and tell you, So-and-So has had a stroke.

#### Q: We were just talking about this as part of the screening before the interview, but how did you learn about having a stroke on your scans?

#### A: Just previous the doctor will see it and she told me about it. And she told me that you will be coming and talking to me. So that's how I learned.

#### Q: Do you remember what type of doctor she was? A: No, I don't, I'm sorry.

#### Q: Okay, that's all right. That was one of the internal medicine attending physicians who came to talk to you about that. How did she describe it to you? What sort of information did she use to, or what sort of information did she give you? A: Well, she said you looked into it and it showed that I have had a previous time a stroke, and I just said to her, It was a big surprise, I had no idea at all. No one has ever mentioned that to me.

#### Q: So you're here in the hospital. You've been here for a few days.

#### A: Yes.

#### Q: Tell me a little bit about what brought you to the hospital.

#### A: I had a fall in the morning. It was a beautiful day outside. I decided to trim some of my bushes. And I went outside and I was standing on the edge, and the most important thing I think, I had flip-flops on. And one of the bricks gave out and I fell. And I fell terribly. Oh, my god, I'll never forget the sound that was in my head. I hit my head so hard.

#### Q: Oh, wow.

#### A: And there was no one– I was able to lift up my head. But then I realized I couldn't lift my left side of the body. And so I decided I was in the yard and decided if I could get to the door, thinking that I would be able to get in the house, and I rolled. And I was close to the door and one of the neighbors came out and they wanted to know if I was okay. And I said, No, but I would like somebody to get my phone because I got as far as the door. But there was no way I could lift myself to get in, never thinking about that. So she came over and she went in and she got the telephone, and I called my daughter. And I said to her, Please come over. And she says, I'm not coming over, I'm calling the ambulance and I'll meet you at the hospital.

#### And this is how I'm here. I am here since, I think it's about five days now, I'm not sure.

#### Q: And you had a fracture, is that right, in one of the bones? A: Yes, I had a hip replaced earlier, about four years, maybe. And that's the leg I broke. And I broke all the bones around the hip replacement. I guess the hip replacement was fine, but my bones were all broken. So they had to replace a new one, a longer one somehow.

#### Q: Oftentimes as a part of the evaluation, the testing when somebody has a fall is they'll do scans of many parts of the body, and they did a scan of your head, which showed these two small old strokes. When we find that, sometimes people will use a number of different descriptors or terms. Sometimes people will call these silent strokes, or silent brain infarction, silent cerebral infarction, covert stroke, covert brain infarction, subclinical stroke, a subtle stroke, or an asymptomatic stroke. Did anybody ever use these terms in describing this to you?

#### A: No. Because actually I never really talked to anyone about strokes.

#### Q: When the doctor came to talk to you about this morning, was there a particular term that she used to describe these? A: I really can't recall.

#### Q: That's all right. I think you mentioned, it sounded like they were prior–

#### A: Old?

#### Q: Or old–

#### A: Prior. Yes.

#### Q: –or something like that.

#### A: They were prior. And that you were looking into it.

#### Q: Sometimes people will use that as the description as well. And without me weighing in on this or your doctors, hearing about that now, these old strokes, and putting that into the context of the fall that you had, do you think if you were on your own, you would connect those two somehow? Or do you think that they're unrelated? A: You mean the fall and the stroke that I have had before? Q: Yeah.

#### A: I think it's unrelated, because the way it happened, I kind of, you know, I brought that on for myself by being out there and the way I put my foot down.

#### Q: Did anybody use the terms incidental or unexpected when describing these to you? A: No.

#### Q: No? Okay. Did the doctor this morning give you any particular advice about this? A: No.

#### Q: No. Okay. And I know you haven't had a whole lot of time to think about it, just since this morning, but hearing about having had a stroke, are you concerned about that? A: Yes. It frightens me.

#### Q: Why? Why does that frighten you? A: Well, because a lot of times the stroke affects the mind. [crying] And that is not how I want to die.

#### Q: I understand. Do you think that these old strokes are connected at all to your other medical conditions? A: I have no idea. I couldn't– I have a lot of stomach problems and stuff like that. So I really have no idea if that would have anything to do with the stroke.

#### Q: What other medical problems do you have? A: I have a thyroid, and– I have a thyroid condition and then I have– I'm trying to think now. I have had different problems. Most of it is all stomach problems. And I have arthritis.

#### Q: Arthritis.

#### A: Yes. Alzheimer's arthritis. Not Alzheimer's.

#### Q: Osteoarthritis? A: Osteoarthritis.

#### Q: So you mentioned that you're frightened hearing about this.

#### A: Yes.

#### Q: Did anybody in your family ever have a stroke? A: No.

#### Q: You just mentioned some of your friends.

#### A: Just friends. No, my family, thank god, they were all– my mother lived till she was about 84. And she was very, very– as a matter of fact, she died in– the accident brought it on. She decided to clean her bathroom and she stood up on the chin[?] She fell and she fractured her hip so badly. But before that, she used to run– she was slower, but she ran her own house. And if you want to visit and you wanted to do something for her, she would say, Did you come to visit? If you didn't, then leave.

#### Q: Let me ask you a question kind of putting this a little bit into context. I did mention, and the other doctor mentioned these strokes. You mentioned thinking about stroke causing weakness on one side, or being disabled on one side. You also mentioned that you have some fear about how it might affect the mind and not wanting to die that way, with your mind affected. I think one thing that is important to put into context is that your doctor described that these probably happened in the past. And you are the way you are right now. And so, knowing how you feel right now in terms of the way you think, and knowing that these happened in the past, does that affect that fear or that concern about this? The fact that these are old? A: Yes, they do affect. I mean, you're thinking, well, if it happened before, it can happen again. I don't know, maybe even it's happening right now, the way my mind is going.

#### Q: Do you think that having had old strokes or silent strokes in the past, do you think that puts your health at risk in some way?

#### A: I don't have an idea how that affects my health, really. I mean, it affects the mind.

#### Q: The health of the mind.

#### A: Yes, yes.

#### Q: I'm actually, so this is to kind of explore that idea a little bit more. I'm going to mention, I'm going to give you a list of a number of different medical problems and also symptoms and ask you if you think that having old strokes or silent strokes puts you at greater risk for any of these things. So that's the frame of the question. And I'll mention the conditions or symptoms one by one. And you obviously don't need to have known about this before. This is just exploring how you may connect things or think that they're not connected. And you can say yes, no, or you can give some answer in between. Okay? A: Yes.

#### Q: So again, for which of the following do you think you're at higher risk now that you have this diagnosis of prior or old strokes. So the first one is stroke. Do you're at higher risk of stroke?

#### A: See, I'm saying all this only guessing. I don't know anything about–

#### Q: Just your best guess.

#### A: Probably, because you had it, so it's probably more likely to happen again.

#### Q: Do you think you're at risk for having a bleed in the head? A: Well, I hope not. Because that's what I was afraid when I fell, and I didn't fracture my head on the outside. I was very afraid that I might be having a bleed inside of my head.

#### Q: Do you think you're at a risk of a heart attack having had a silent stroke?

#### A: Well, because this does give drain[?] to the rest of your body, I think. You probably do.

#### Q: Do you think you're at risk of developing heart failure from having a silent stroke? That's when the heart's not pumping as well A: Could be.

#### Q: Do you think you're at risk of having high blood pressure because of the silent stroke? A: Well, I have had high blood pressure.

#### Q: Already.

#### A: Yes. So. I'm with that.

#### Q: How about high cholesterol? Do you think you're at risk of developing high cholesterol because of– A: I have that, too.

#### Q: You do, okay.

#### A: Yes.

#### Q: That's sort of a non-issue.

#### A: Yes.

#### Q: Okay, how about diabetes? A: Oh, thank god, so far I'm okay as far as that. I don't have anything.

#### Q: Do you think having a silent stroke puts you at risk for that?

#### A: I really can't say. I don't know.

#### Q: That's all right. Again, these are just best guesses. How about headache? Do you think silent strokes might cause headache?

#### A: Yes, probably do. Q: Do you think silent strokes cause vertigo? That's when there's a spinning sensation.

#### A: Yes.

#### Q: You think so? A: It could be. Q: Do you think silent strokes can put you at risk for seizures? When people stiffen and shake.

#### A: Yes.

#### Q: You think so? A: Could be, yes.

#### Q: How about falls? A: Yes.

#### Q: Do you think it puts you at risk for memory loss? A: Yes, I think so.

#### Q: Dementia? A: Yeah, probably.

#### Q: How about anxiety? A: Could be, because it sort of all, everything goes together.

#### Q: And depression? A: That's right there, too.

#### Q: And the last one is schizophrenia. Does silent stroke put you at risk for that?

#### A: I really don't know.

#### Q: That's all right. Again, these are just best guesses.

#### So next question is: now that you've learned about this, is there anything that you would want to change at this time in terms of how you live or sort of what you do?

#### A: Well, if I know what would help to prevent it happening anymore.

#### Q: Do you know of any things already in terms of things that might prevent–

#### A:

#### Q: I know, this is relatively new information for you.

#### A:

#### Q: We're coming close to the end, but I want to ask you a few hypothetical questions. So we're used to treating patients with stroke that have symptoms. But if I told you that there are no current national guidelines or research studies that guide doctors in terms of selecting tests or therapies for silent stroke, for these strokes without symptoms, how concerned would you be about that?

#### A: Can you repeat that? Q: Sure, it's a complicated question. So oftentimes when we are figuring out the best therapies or tests for a medical problem, we do research to try and figure out what's the best thing. And when there's enough research, we convene some experts in a panel and then they come up with what we call guidelines, which is just a set of best practices to help the whole field know for most people what we should do. For silent stroke or asymptomatic stroke, there are actually no national guidelines and no specific treatment studies that help physicians in terms of knowing what to do. We have studies for stroke with symptoms, but not necessarily ones without symptoms. And so, if you knew that, that there are no guidelines or prior studies, would that make you concerned? Are you concerned about that lack of information?

#### A: Well, then it kind of makes me feel at ease more? Q: It makes you feel at ease? [laughter] Why is that? A: Well, because they're not looking into it yet, so maybe it's not as affecting.

#### Q: It's not as what, I'm sorry? A: It's not as common or affecting people.

#### Q: Does knowing that there are not any guidelines or studies about treatment, does that affect how you would perceive any advice that your doctors can give you about what to do for your health?

#### A: No, I would take the doctors' advice, whatever they tell me to do. I really would. Then if you're not going to, then why go to the doctor?

#### Q: Sure, sure. That's an interesting response about feeling a little bit more at ease. And I think it's one that sometimes some patients will describe. Interestingly though, it actually is very common. So above age 50, we now know that about one in five people has a silent stroke on a scan of their brain. Without necessarily having any obvious symptoms or problems. I guess my next question following that is: how important would it be for you that this be studied, or that people look into this? A: I think it would. It would be for yourself and would be for others if it can happen again and is more severe.

#### Q: I asked you these questions about things that silent stroke can cause, and actually what we do know is exactly as you sort of had an intuition about, it does increase the risk of having a stroke with symptoms by about two- to fourfold. So two to four times. And it does increase the risk of dementia as well, about two to three times. Although that's over a longer period of time. So we do think that it's important to try and understand what to do after we find these.

#### And again as a hypothetical, let's say we do do some studies, some prevention studies, and we find that– let's say we're trying to prevent a stroke. And we find that making some lifestyle changes like exercising or maybe starting a medication like aspirin, let's say that finds some health benefit in terms of preventing stroke, but it's a relatively small benefit. Do you think that you would be motivated to make these changes? Like adding on another pill or engaging in an exercise program? A: Yes, I think I would.

#### Q: You think so? A: Yes.

#### Q: How much of a change do you think you'd be willing to make just hearing about an old stroke on a head scan that might have happened in the past? A: I would consider even going to a lecture if someone is talking about it, or even reading about it.

#### Q: So you would want to learn more about it.

#### A: Yes.

#### Q: As we're doing studies to try and figure out how to prevent stroke and prevent other things, are there other things you would want to know about, or any other outcomes we should look at, things that are important to you? A: Well, I'm trying to think what would be–

#### Q: Sure. Yeah, no, take your time.

#### A: The heart is very important to [23:53]

#### Q: You'd want to make sure that this doesn't have an effect on the heart? A: Yes. That it doesn't have an effect on my family.

#### Q: On your family? A: Mmm.

#### Q: What sort of effect do you think this might have on your family? Or what would you worry about? A: Well, I would worry about it that it affects your mind or something like that if it's more severe type. Because probably the one that I have had, it was not that severe because if I didn't notice and no one else did–

#### Q: So just to explore that thought a little bit more, you're worried about how silent strokes might affect your thinking and you wouldn't want to be in a situation where you would be dependent on other people? A: Right, that's the most important.

#### Q: My understanding is you live alone and you're completely independent and take care of yourself.

#### A: Yes.

#### Q: Which is admirable at 83.

#### A: Yeah. And I still drive a car.

#### Q: You take care of everything.

#### A: Yes, I do everything my own.

#### Q: So maintaining independence and being able to take care of yourself.

#### A: Yeah, I have a six-family house that I need to run.

#### Q: Oh, wow, okay.

#### A: You know? Q: Okay, that's very important for us to know, maintaining independence and being able to take care of other things.

#### A: Yes. But I think now I'll have to ask for some help.

#### Q: It can be in stages, certainly. There's no shame in that. So we're actually coming close to the end of the interview, but I wanted to hear if you had– first, after talking about this, has your perception of stroke or stroke without symptoms changed? A: Well, it has, because I never knew there was such a thing at all.

#### Q: Do you have any additional thoughts or questions that you would want to ask? A: Well, not right now. Right now I'm very concerned on what is happening now. And once I get over that, then I'm sure I will be more questioning.

#### Q: Again, I'll leave you my contact information so we can always talk or you could always come see me in the clinic.

#### A: I'm sure Dr. A [?] will hear about it.

#### Q: Yes. I'm in contact with her frequently. And also, I'll spend just a couple minutes talking to you after this interview as well.

#### A: Thank you.

#### Q: I'll stop the recording right here.

#### END INTERVIEW

**Participant 7**

**Q:** All right, so this is the interview with our seventh patient in the study. Can you say hello?

**A:** Hello.

**Q:** Okay. So I'll go ahead and ask you a few questions. There are about nine questions, but depending on how talkative you feel, I might ask just a couple of other questions to get you to sort of explore your thoughts. But the first question’s very open-ended. I want you to tell me what you know about your diagnosis so far?

**A:** All I know about my-- I had a stroke in my head. And there was a clot of blood and it probably come from my heart, but I [00:00:45] for right now. So, I don't know nothing else.

**Q:** Okay.

**A:** All the time I may get some weakness in my feet, yes [00:00:59] weakness in my feet. And before that I used to bite my tongue. I wonder why is it I bite my tongue instead of my jaw? I wondering why it happening. So that's all I know about the stroke.

**Q:** Okay, fair enough. Who’s the person who told you that you had a stroke?

**A:** Dr. N.

**Q:** Your primary doctor?

**A:** My primary care doctor.

**Q:** And what were the circumstances?

**A:** I had some headaches and [00:01:42] got these headaches and they're going down my neck. And she sent me for MRI and then when I got home, she called me back and said ten minutes [?] emergency room, I had a stroke.

**Q:** Okay. Were there any other symptoms besides the headache around that time?

**A:** No.

**Q:** No? Okay. Was the headache different or unusual in any way?

**A:** I used to take me like a needle, like needle [00:02:11] me this way.

**Q:** On your left side?

**A:** On my left side, needle shot me, jamming me.

**Q:** My understanding is that you've had headaches in the past?

**A:** Yeah, I had headaches in the past. Aneurysm clipping, and I used to have a lot of headaches. Since I had the clipping, I had not very bad headaches but headaches and they stop, they stop for a while. It’s only then they’ll start back up again.

**Q:** Okay. And the sort of stabbing sensation, was this different than the headaches you had in the past?

**A:** Yes, it was like needles jamming me in the side of my head.

**Q:** Okay. So, I mentioned a little bit about the nature of the study and how we're trying to explore strokes that didn’t have an obvious sort of connection to symptoms or obvious symptoms when the brain study was performed. And a lot of people have come up with different ways of naming this, just different terms to refer to it. So sometimes people will call them silent strokes or a silent brain infarction, a silent cerebral infarction, a covert stroke, a covert brain infarction, a sub clinical stroke, a subtle stroke or an asymptomatic stroke. Were any of these terms used, or did you remember ever hearing any of these terms?

**A:** No, I didn’t. This is a silent stroke.

**Q:** Okay. But nobody had sort of mentioned it in those terms?

**A:** I can't remember, tell you the truth. They tell me it was a small stroke.

**Q:** They called it a small stroke?

**A:** Yes.

**Q:** And you described the headache that brought you to your primary doctor and then the brain MRI was performed, and then she told you you had stroke. Did you sort of put two and two together? Like, did you think that the stroke was connected to--

**A:** The headache?

**Q:** The headache in any way?

**A:** I think so.

**Q:** Okay. Did any of the doctors talk to you about that or sort of build that connection?

**A:** Some of them-- one of them tell me that headache don’t come from strokes or something like that. And like I don't know, I started getting these headaches and that is why I went to doctor. It was sticking me like needles in my head, in my temple right here. And, oh, then my neck started hurting, like-- I didn't like it.

**Q:** Sure. Did anybody describe the stroke on the MRI as being incidental or unexpected?

**A:** No. I didn’t even see the MRI.

**Q:** Okay. Well, we’ll take a look at it afterwards.

**A:** I didn't see it.

**Q:** So they just described what was seen but didn't comment about whether or not it was an unexpected finding or an incidental finding?

**A:** Right.

**Q:** Have you heard those terms used before, actually, incidental or sort of unexpected finding? Do you know what I mean by that?

**A:** I heard those in the end already, but--

**Q:** Okay. What type of advice were you given by your doctors when this was found?

**A:** They didn't tell me, [00:06:14] me anything. All they tell me is about the stroke. I can't remember them telling me nothing else.

**Q:** I guess your primary doctor was the one who ordered it and then she told you to go to the emergency room, right?

**A:** Yes.

**Q:** Did she mention anything else at the time?

**A:** No, I saw her afterwards and she told me that I have a little stroke in my head and what else she tell me? Oh, and that she wanted me to see the diabetic doctor immediately.

**Q:** Oh, I see. So let me just clarify. When you had the MRI done, did you see her immediately after the MRI and before you went to the hospital? Or did you see after the hospital?

**A:** I saw her before I had the MRI, and she called me and sent me to emergency room and I saw her, I think, a week or maybe after I come out of the hospital, yeah.

**Q:** Okay. What was that conversation like when you were on the phone with her?

**A:** When I was on the phone with her, she tell me, “Miss Phillips, I'm getting results from the MRI and you have a small stroke in your head.” And I said, “Huh?” And she tell me she wanted me to go to emergency room right now. She asked me what time I could get there and I tell her about 6:30. I was at the nail salon getting my nails done.

**Q:** When you got that phone call?

**A:** Uh-huh. I leave the hospital and I went straight to [00:08:19]. I had just get the MRI and then the nail place. And then I called my son, but he’s supposed to pick me up at 6:00. So when he picked me up, I tell him that I go to the emergency room, doctor tell me I have a stroke in my head. And he dropped-- I said I wanted to go home and he dropped me home and dropped me to the hospital [?].

**Q:** Okay. Did your doctor sound very concerned over the telephone?

**A:** Yes. She very concerned about me.

**Q:** And how did you feel when you first heard that, actually?

**A:** I didn’t feel no way. I didn't feel no way because, like, I didn't know-- I worried people that had strokes and all sorts of things for nearly 20 years, and I couldn’t believe that I had a stroke. I do [00:09:33] for all these years and all the people that got strokes and I had stroke [00:09:37].

**Q:** Uh-huh, okay.

**A:** I'm a care-- I didn’t care, I didn't think [00:09:51] me all the time. Anybody tell me anything that wrong with me, I just don’t care, whatever happen I won't. That's me.

**Q:** Okay. What type of work did you do before where you were working with people with stroke?

**A:** Home health aid.

**Q:** A home health aid? Okay. So after your doctor told you, your primary doctor told you, that you had the stroke and now that you've gone through the hospital and then saw your neurologist in the clinic again, what are your concerns about having this diagnosis?

**A:** My concern about how I get a stroke, how it happened. My blood pressure wasn’t high. I didn't know if my blood pressure was high at the time, but my blood pressure wasn’t high. So, I don't know how the stroke occurred.

**Q:** Okay. So you're wondering what--

**A:** I wondering what caused me to have that stroke. That the next time I will know what to look for. But I don't know.

**Q:** Okay. How concerned are you about having had this? So you're sitting here with me in the office. It seems like you're doing okay except for the back pain that you've had, but you seem otherwise well. How concerned are you that you had this silent stroke?

**A:** A lot. I'm concerned a lot. I'm concerned about how it happened, what make it happen, for it happened-- I live alone. For it happened, like biggest [00:11:43] and I'm alone. What happen right now if I bend over to pick up anything and I get back of my head will be spinning. I'll got [00:11:59] all the time if I bend over. Even if I hold over the basin to brush my teeth. When I hold back up my head, it is spinning. And that is some [00:12:09]. I never had that before. So, that is another thing I'm concerned about.

**Q:** When did that spinning sensation start?

**A:** That start like must have been same time I started having these jamming pains in my face, in my face.

**Q:** The headache that--?

**A:** The headache. If I bend over-- like if I go in the fridge, I'll open the fridge to take out anything, I got to come up a whole [00:12:43] on my head will be just spinning.

**Q:** So you actually see a spinning, like a rotation feeling or sensation?

**A:** Yes.

**Q:** Out of curiosity, do you recall-- so we call that vertigo when you see the world spinning around you, or rotating around you like a merry-go-round. Out of curiosity, do you remember mentioning that to either your primary doctor or the doctors in the hospital after you had the MRI?

**A:** I can't remember.

**Q:** Okay, just curious.

**A:** I can't remember mentioning that. But I know I mentioned to the nurse when she come that when I fall down, when I fall over, I get back up, my head is spinning.

**Q:** Okay, so you mentioned that to a nurse?

**A:** Yes, and [00:13:36].

**Q:** Was this a nurse in the emergency room or in the hospital, clinic?

**A:** No, the nurse at the-- the visiting nurses.

**Q:** Oh, visiting nurse? Okay.

**A:** Yes.

**Q:** After the hospital?

**A:** After the hospital.

**Q:** Okay. Moving on to a slightly different question, do you think that the silent stroke was connected to any of your medical conditions?

**A:** I don't know. I wonder. I got hypertension, I got constant back pains, I got diabetes. I don't know. I had a brain aneurysm, I don't know.

**Q:** Okay. The next question I want to ask is so I think you already have some-- sounds like you already have some background knowledge of stroke having worked with people who had had strokes. In terms of this one being a silent stroke, without any sort of really obvious symptoms, this may seem like an obvious question that I'm asking you, but I want to ask, do you think it puts you at risk for anything in terms of your health? And if so, what does it put you at risk for?

**A:** Well, it puts me at risk at if anything happened-- if I had a bigger stroke, I wouldn't be able to help myself and do the things that I like. I like helping myself. I like doing things for myself. I don’t like people doing nothing for me. Not even my children. My children just come clean, but I don’t like them to do nothing for me. I like my house a certain way and I like to clean my house a certain way and them don’t like how I clean my house or I like-- I like doing things myself. I like things in place. If I leave my house and I come back at my house, I could tell anybody that somebody went there. Because I just know when things move and I think in the same place. That is what I like.

**Q:** So it sounds like you're worried that this might put you at risk for having stroke that causes a lot of problems for you, particularly--

**A:** Yeah, like stroke that have--

**Q:** Like some disability with it?

**A:** Right, you lost your side, the one side that's weak and not-- but my mother had a couple of strokes and she didn’t lose nothing. She was still walking and everything. Like, she had a-- know how old people got strokes and the hand or the limb or something, my mother had strokes and she didn’t-- I don't know--

**Q:** She didn't develop weakness?

**A:** No. I don't know about the last stroke she had, because every time she-- every time that she have a stroke, my sister would call me and I would go home and I would see her. And I would take care for a couple of weeks and then come back. But the last stroke she had, the doctor sent her to the hospital. That was the final stroke. So, I don't know how it was because when they call me and I get there, she was already dead.

**Q:** I'm sorry to hear that. What I'm going to do is I'm going to ask you-- I'm going to actually give you a list of different medical conditions or symptoms that people might have, and I'm actually going to ask you if you think that having a silent stroke puts you at risk for any of those things. So does the silent stroke cause these issues and-- I'm actually just going to go down the list and you can say yes or no or I don't know or some other answer if it’s more complicated than that. And this is an opinion question, so I'm not expecting that you know any of this information already, but I want to see if you connect these in any way.

**A:** Okay.

**Q:** So again, the question is do you think that having silent stroke puts you at risk for any of these? So the first one I think you've already answered. Do you think silent stroke puts you at risk for having a stroke with symptoms?

**A:** Yes.

**Q:** Do you think that it puts you at risk of having a bleed in the head?

**A:** Yes, because I'm already tell me I got a clot of blood in my head.

**Q:** A bleed, what we call a hemorrhage, is when the blood vessel breaks.

**A:** Oh, it breaks, right.

**Q:** Do you think that having a silent stroke puts you at risk for that?

**A:** I think so.

**Q:** How about do you think it puts you at risk for a heart attack?

**A:** Yes.

**Q:** Do you think it puts you at risk at what's called heart failure, where the pump is not as strong? The heart’s not pumping blood as well through the body?

**A:** Could happen.

**Q:** Okay. Do you think that having the silent stroke puts you at risk of having high blood pressure or higher blood pressure than what you have now?

**A:** A higher one.

**Q:** Do you think so?

**A:** Could happen.

**Q:** Okay. Do you think that having a silent stroke puts you at risk for high cholesterol?

**A:** Maybe.

**Q:** Okay. Do you think that it puts you at risk for-- I know you already have diabetes, but do you think it puts you at risk for having your diabetes get worse?

**A:** Could be.

**Q:** I think you answered this one already, but do you think that having a silent stroke puts you at risk for headaches?

**A:** Yup.

**Q:** Do you think that a silent stroke puts you at risk for having vertigo, which is that spinning sensation?

**A:** Yeah, I've got all that now and my head spins so.

**Q:** Do you think that having a silent stroke puts you at risk for having seizures? That's when the electrical activity in the brain short circuits and people might fall to the ground and shake?

**A:** I know what seizure is that. I think so, and I hope not.

**Q:** Okay.

**A:** Because I don’t want seizures.

**Q:** Sure, of course. Do you think that a silent stroke puts--

**A:** I got two-- my sister and my nephew has got seizures all the time.

**Q:** Oh, really? Okay.

**A:** I don't know why that.

**Q:** Okay. Do you think having a silent stroke puts you at risk for falling?

**A:** Yeah. Because sometimes your head gets-- sometimes my head gets so [00:21:44] I think that I will fall, but I hold on. That's why I don’t fall down no more, I try to hold on. Because I don’t want that.

**Q:** Okay.

**A:** I get that little thing, that I pick up-- what I got to pick up. If there's anything that [00:22:08] when my grandchildren come I tell them to do it for me. I don’t want to fall.

**Q:** Do you think having a silent stroke puts you at risk for memory loss or having trouble remembering?

**A:** Some place people get strokes and they miss [00:22:33] and they can't remember certain things. Yes, I don't know if a silent stroke will do that, but I know a stroke will do that.

**Q:** Okay. Do you think a silent stroke puts you at risk for developing dementia?

**A:** I would hope not. But it could happen. But I would hope not.

**Q:** Do you think that a silent stroke puts you at risk for developing anxiety?

**A:** I don't think so. But I think so.

**Q:** You don’t think so, but you--?

**A:** I don't think so, but some people body is different to some so it may not happen to me, may not happen to you, but it may happen to somebody else.

**Q:** Okay, fair enough. Do you think that a silent stroke might put you at risk for depression?

**A:** Depression and dementia is like the same thing, but one is why you can remember things that happened years ago and things that happen right now you can't remember. So I would think so, in your head, stroke in your head do anything to your brain.

**Q:** Okay. And then the last one is do you think that silent strokes put you at risk for schizophrenia, which is when people have some sort of distorted perception of reality?

**A:** I would think so.

**Q:** Okay. And so the next questions I want to ask, there are only a few more questions left, is how did you-- when you received this diagnosis and afterwards, how did you describe this news to your family and friends, if you mentioned it to them at all?

**A:** I don’t hide my sickness from nobody. My friends not my children. I call them up and I tell them. In fact, I tell-- I call my son bring me here, and my grandson and he tells his son that he came to the hospital, I have a stroke. And his son calls his aunt, his nephew, his sister, they call everybody. So everybody called me at the hospital. How come that may know, how come that I now find out so I can tell. I call my friends, I tell them. My best friend knows the same time the doctor called me, I called her. And then I call my son or he called me. My daughter called him and she called my two sons in Barbados and them called me. So I don’t hide my sickness from nobody. It’s not right to hide sickness from no one.

**Q:** That's a very powerful thing, being able to share that with your family and have all the support you-- your friends as well.

**A:** Exactly. So everybody asks how it happened. I don't know how it happened, they want to know how it happened now, so.

**Q:** That's a question that they asked you, oh, you had a stroke. Why did that happen?

**A:** What happened? What have you been doing, and all this. I don't know what happened.

**Q:** Okay. That's an important question, I think and an important thing for people to discover and learn. How did you describe-- so when you left the hospital, how did you describe this to your doctors or to other people that you encountered? For example, you saw the neurologist, I think you mentioned you were going to go see the diabetes doctors. Did you mention to them, “Oh, you know, I was just in the hospital?”

**A:** Yeah. I tell I was just in hospital, just had a stroke. And they tell me go see [00:27:35] report.

**Q:** Okay. And thinking back, do you think-- I guess sort of thinking back in terms of when your primary care doctor first described the MRI to you and told you to go to the emergency room and also the whole hospital experience and the clinical visits afterwards, reflecting on that, how would you have wanted this to be explained to you in terms of having this stroke?

**A:** Okay. I wanted to explain to me how it happened, how I get it, and how it occurred.

**Q:** Okay. At this point in time, it’s been a few weeks, do you think that there's anything that you would change now that you've learned about this?

**A:** No. First give me-- oh, I was going to say exercise and my doctor tells me I can't do exercise for the next three weeks.

**Q:** Because of the stroke or because of something else?

**A:** No, after I had the stroke, and I saw her and I tell her that I was going in the gym with my grandson and she tell me that I can't go, give myself about three weeks before I go.

**Q:** Okay.

**A:** But I said okay. Because I said I probably need to lose some weight, I probably need to lose-- I eat very healthy, although I eat a lot, but I eat things like-- I eat things like old people would eat, a lot of starchy food and a lot of this or that. I eat rice once a week and that is on a Sunday. I mostly drink a lot of vegetable soup and not out of they can, that I boil. I boil it with like vegetables, [00:30:22] vegetables, and meat. Any kind of meat and vegetables. And that's it.

**Q:** Okay.

**A:** And for breakfast, I eat a sardine with [00:30:48] and lettuce and a cup of sea moss or cup of oatmeal or chicken salad that I make. And at lunchtime, I probably eat a yogurt and a banana for lunch. So, I eat that kind of food.

**Q:** Okay, so it sounded like after you learned about the stroke you were thinking a little bit about your diet and food and trying to get--

**A:** No, no. I using that before I had stroke.

**Q:** Right, but I'm just saying that when you had the stroke, you thought about it but then kind of came to the conclusion that actually your diet’s good, that in terms of your balance of food, but I guess you were thinking a little bit about losing some weight. You were thinking about exercise.

**A:** I was thinking about it before I had-- before I knew I had a stroke I was-- but I don’t want to go into the gym alone, so my grandson was-- he was planning to go in the gym from that week. He visits gym every day. And he’s your size. So, he left college and go to the gym. So he tell me, “Grandmother, I can sign you up in the gym and we will get together.” And my daughter-in-law, my granddaughter, they was going to the gym, too. So, since I had the stroke, but they tell me I can't go.

**Q:** To wait a few weeks before?

**A:** Yes, three weeks.

**Q:** Okay. Let me tell you, this is a little bit of a sort of hypothetical question. But if I told you that currently there are no national guidelines or no specific research studies that guide physicians in terms of selecting tests or treatments for silent stroke, how concerned would you be about that?

**A:** Real concerned. I'd be like no kind of treatments, no kind of research? So, how you go about this?

**Q:** I think I should clarify that we know a lot about stroke with symptoms, with neurologic problems. But this is kind of an interesting issue, that silent strokes, we're trying to figure out is that the same thing. Should we be treating it the same. And so that's one of the questions that we're trying to figure out. But knowing that, that we don’t necessarily have the guidelines or the research studies to say with 100 percent certainty this is the right thing to do, does that change how you perceive the advice that you received from your doctors?

**A:** Yeah, I've been studying about like-- you have a stroke, there's no medicine, nothing you could give for it, and nothing you can do for it? I would be concerned a lot.

**Q:** How important is it to you that this be studied more for us to figure those things out?

**A:** How concerned for--?

**Q:** Not concerned, but how important is it to you?

**A:** It’s very important. I would like to know.

**Q:** Okay. Let’s say we do some studies in the next year or so to try and figure out how to prevent stroke after someone has a silent stroke, or prevent other bad things that might happen. And we find a little bit of a benefit. So a small benefit, but definitely one that's there, with a medication or maybe like exercise, a lifestyle change. If those studies actually showed that might have a little benefit from, let's say, exercising twice a week or taking an aspirin or something, would that be enough to convince you to make those changes in your life?

**A:** Yeah.

**Q:** Okay, so kind of like a little bit?

**A:** I take an aspirin now, going to take me off the aspirin. I used to take an aspirin every day.

**Q:** But now you're taking a different medication, is that right?

**A:** Yes.

**Q:** Okay. So, we're almost done with the interview. I want to see after we've talked over the past half an hour, has your perception of silent stroke changed, your thoughts about it?

**A:** Yeah.

**Q:** How has it changed?

**A:** Because listening to you and some of the reports that you give, it helps me a little because then I know that you can take a stroke and certain things happen. And I still wondering why I have a stroke and I didn't have no kind of symptom to show me that I had a stroke. Not even remote [00:36:54].

**Q:** If the mouth twisted? Yeah.

**A:** I wouldn't know. Even I know I bite my tongue if I talk or eat, I will bite my tongue and it never-- it never happened to me. So when I start biting my tongue, what happening to me, I'm biting my tongue? And that was a little concern. So I know that stroke bring a lot of different things [00:37:35] I really had all these symptoms that I should had, you understand? I know this leg get weak, something.

**Q:** Okay. Now that we've come to the end of the interview, do you have any additional thoughts or questions on your mind about this topic?

**A:** Yeah. I would like to know if there is a treatment or if when they find a treatment, if I will know anything about it. If no one contact me, tell me anything about it before I have another one. Or just tell me we’ll call this first one, but I will know what to do.

**Q:** That’s great. Thank you for sharing that. I'm actually going to stop the recording right now and we can talk a little bit more about it.

**A:** That's okay.

**Q:** Thank you very much.

**A:** You're welcome.

END OF INTERVIEW

**Participant 8**

#### Q: Okay, so this is the interview with our eighth patient. Can you say hello?

#### A: Hello.

#### Q: Great. So what I'm going to do is I'm going to walk you through a number of questions. There are nine questions. And then there are a few kind of sub questions that come along with it. But the idea is just to express your thoughts and your ideas around this issue.

#### So we talked a little bit about your diagnosis about having a silent stroke, and so my first question for you is just to describe in words what do you know about it or what do you think about it?

#### [00:00:35]

#### A: What do you mean, what do I think about it? Q: What is your diagnosis? [00:00:42]

#### A: My diagnosis? They tell me that is stroke. Yeah. And I was surprised when they tell me, because before I didn't have some symptoms about stroke, only pick me up about on 24 April at night, two a.m., in morning, early morning, I remember. It was Wednesday, 24 April. And make me dizzy. Dizziness. And I need very careful wake up and start walk. And keep on the wall because my balance was so no good. And I try return to my bed and try be quiet, keep control about my dizziness. And again started. And I didn't want nobody pick up. I think that be past because people live. My husband sleep and my daughter sleep.

#### [00:01:48]

#### And about four hours in morning early, I have some acid stomach problem that I some– I don't know how to say.

#### Q: There's like some acid in the mouth.

#### [00:02:02]

#### A: Yeah. And I see when something problem with stomach, I see no good that something is happened and I pick up my husband and my daughter and we call emergency. We didn't ready that somebody of my family driving in emergency. We call emergency. And drive me to hospital in Tufts Medical Center when I am patient about 19 years.

#### And they first looking regular blood pressure, how is my blood pressure, keep control and every doctor which coming on that night ask me similar question. And I talk what happened. What happened. And they were, I think, they don't know what's happened, but when I tell to doctor, I don't know how call, Sullivan?

#### Q: Yeah, Dr. S.

#### [00:03:09]

#### A: Yeah. He was third doctor. Every time I answer same story about that night. And I tell to him that I remember only in November, December when I walk with my doggie, street is good, clean, normal. My shoes is for walking. And my left leg turn on the side and I fall on the right side. I try keep my head no broken and I a little broke my knee and my right arm. And I think that was okay, only I fall. And I didn't know what's happened.

#### And when I tell to doctor, when I tell this story, I was going to [4:03] Center on Route One on second day because my arm is, how say, swallow?

#### Q: Swollen, yeah.

#### [00:04:13]

#### A: Yeah. And then I see that there's something no good and doctor take X-ray and nothing. Bone is okay, but my ligament a little is ruin and they gave me some that I keep mobilization my hand a few days and keep ice. And when I tell everything about that to Dr. S, he send me to MRI on that night. And in morning– that was Wednesday morning about– we are waiting result about two hours. Yeah, about ten in morning coming fourth doctor, I didn't see him before. And he said, "You have been stroke."

#### Q: Is that the first time you heard that?

#### A: Yeah. And I was surprised. I was under shock how I had stroke, because I didn't feel nothing what is special. I know, my mother had stroke and I know symptoms how, what's happened with her and how she looking and what to do with her body, with her talk, face. For me, it's nothing. They take control. Again, every doctor look in my reflex of my legs, my arm, my face, my looking left/right. Everything was normal. And I didn't have symptoms. But MRI show that I had stroke.

#### [00:05:55]

#### And they tell maybe in the last three days that's happened and maybe most of about two weeks. And I try remember. They ask me, I don't know which doctor. They asked me did I remember something happen with me. I remember on the day when is something different when I work in library and when I walk around shelves and take books and one moment I feeling when my eyes, left/right, looking left, looking right, I feeling that my eye don't– this eye. I feeling in eye as small ball running. It's ball. And don't pain me, but for me was different. Only that.

#### I tried remember another day when I drove my granddaughter at night only that is two different situation when I feeling that something is happen with me. And I drove car. Was night. On the street had lights, but no much. And one moment I tell to my granddaughter "what is with my eyes? I don't see very good." Because before I drive car at night and looking normal, how we can, everybody looking. I don't use my eyeglass for far. I only use glass for reading.

#### [00:07:36]

#### Only that two something different with me, because they ask, some doctor ask me, "did you remember something is different?" And nothing more. Usually I have headache sometimes because I have been migraine before more. Now is lot, no much. But every time something happen with my head, I think about allergy, because I'm allergy on dust and pollen and mold. And I think that is allergy.

#### And nothing more.

#### Q: Thank you for explaining all of that. So it sounds like the fourth doctor was the one who told you that you had a stroke.

#### A: Yeah.

#### Q: Which was surprising for you because you didn't have any symptoms–

#### A: Yeah.

#### Q: –or stroke symptoms. Just to clarify, before that Dr. S and the previous two doctors, did anybody mention stroke as a possibility? Or was it just something that came up in the conversation with the fourth doctor?

#### A: I don't understand.

#### Q: That's okay. Did anybody mention the possibility of stroke?

#### A: No, no.

#### Q: Before that fourth doctor?

#### [00:08:47]

#### A: No, no. They were confused, what's happen with me because blood pressure keep control. A little is high because I was nervous. But I use pills. And after this, when they saw that my blood pressure is a little high, no much, they gave me one more pills for blood pressure and my blood pressure is now very good, normal, yeah.

#### Q: That fourth doctor, do you recall what type of doctor that was that described to you that you had a stroke?

#### A: What? Q: What type of doctor in terms of their specialty or expertise?

#### A: I don't know. I don't understand.

#### Q: That's all right.

#### [00:09:32]

#### A: This doctor, this very nice doctor, this Sullivan, which tell me that I– yeah, he saved me because he said "go MRI." And for him was something tell because I fall in November. And he as doctor, I'm not doctor, but he think that is some happen with my health, my body. On this side when I fall, that don't stroke, that was before three, four, three months. That something is change in my body, yeah. And thank you for him. Yeah, he founded something, what I didn't know what was, yeah.

#### Q: For you the stroke was a surprise. Did anybody else describe the stroke as being unexpected or incidental or a surprising finding?

#### [00:10:39]

#### A: I don't– I understand, but I don't know what you want. I am surprised. And after this, of three days while I stayed hospital, everything looking control, go X-ray again, looking my heart how work. And I made joke about it because I'm under shock. And we smile. [11:06] coming. Everything what I talk with smile. How I have stroke when I normal work, my leg, my arm, my face, everything normal. And when I come at home, when maybe relax, stay at home, don't go to work because my balance no good. After two months I started work. I couldn't walk very fast. Yeah, because when I come at home, positive shock passed but coming now negative and I am under depression. I was depression of this time. And I was happy when I started work, I feel more better now. I drive car. On first day, my family, "could you drive slow?" I don't live very far, about ten minutes with car drive.

#### Q: What advice did the doctors give to you after you were diagnosed with a stroke?

#### A: What advice.

#### Q: Yeah, what did they tell you to do? Did they change anything for you?

#### [00:12:20]

#### A: No, only this doctor which, that is older doctor, I don't know, I don't remember what's name. Because he said me, but I was under shock when he said me "you have stroke." And I shocked. And I forgot what his name. He only explain me, but my daughter, she try looking how I reaction and she ask doctor "is that because not big symptoms? Is that maybe mini stroke?" And he said, and that's true, stroke is stroke. He said "stroke is stroke." Doesn't matter big stroke or a little stroke. And only that. And he said me that and I got paper about everything that I read, my family read, and what I need to do about my now continue life. I didn't go to– I was only pills for blood pressure one more and for blood clot, how say, Plavix, on pills of Plavix. Is that **clopidogrel?**

#### Q: **Um hmm.**

#### A: **Yeah, clopidogrel.** And nothing more. I only got two pills more, for blood pressure, for blood clot.

#### Q: Okay. When that doctor or any of the other doctors were talking to you after you received the diagnosis of stroke, did there seem to be any uncertainty in terms of what to do? Or they were just like "well, this is a stroke, these are the things you should do"? Did they seem uncertain or hesitant?

#### [00:14:19]

#### A: My doctor, Dr. T, primary doctor, she was surprised. I don't know who tell to her. She got maybe on computer. [crying] I cry now. She coming at night in hospital, coming. She looked so– sorry for her what's happened because she didn't think that I have, that my body, my health is, was good. Keep normal blood pressure, cholesterol, everything. And I'm sorry for her when I see her, how she looked. She was so surprised, hard. And she feels sad what's happened with me.

#### Q: So she came to the hospital to visit.

#### [00:15:05]

#### A: Yeah, at night, at night. She coming. I woke in chair.

#### Q: To check in on you.

#### [00:15:10]

#### A: Yeah, yeah, yeah. And later, I have appointment with her and she explain me what I need to do. Normal, everything but what books write, a little keep control. I can eat everything, you can tell it's true. But keep control. No much sugar, no much fat. But I try, I try to keep control. And exercise and walk. And try– I'm a little more now emotion and every small problem make, ruin my health, yeah. I try isolation[?] of problem. I can't, when somebody tell my granddaughter has fever, I'm under stress. That is no good for my health.

#### Q: I think that is important, to try not to be stressed, to take care of your health. What are your concerns about having this diagnosis? When you heard that you had the stroke, even though it didn't have any symptoms that you connected to it. What are the concerns that came up for you? What are you worried about?

#### A: That I worried. Yeah, I think all time.

#### Q: What are you worried about though now that you have this diagnosis?

#### A: I think when is next? [laughter]

#### Q: When is next?

#### A: Yeah. I think about that. That's coming next, next stroke.

#### Q: So you're worried about having another stroke?

#### [00:16:44]

#### A: Yeah, yeah, yeah. I try don't, because everybody tell me "don't think about that." That's happened, that is past. But that coming no of my wish, coming itself, that's because I know of my mother, yeah. Sometimes coming one and coming another. Yeah.

#### Q: The fact that your stroke didn't have any symptoms, does that affect whether or not you think it's a serious condition, the stroke that you did have? So you were told you had a stroke. You didn't have any symptoms with that. Do you think you would be more worried if it had symptoms? Or are you still just as worried even with a stroke without symptoms.

#### [00:17:36]

#### A: I tried to live with that, but now is low, but after this when I stay at home, be quiet, I think more and coming worry. Every time worry. More worry. But how I started to work, that's better for me because I talk with my friend there and we try make joke about it, because when they see that I'm depression and I scared, they try nice talk with me.

#### Q: I'm glad you have your friends to support you.

#### [00:18:12]

#### A: Yeah, yeah, yeah. And I tell to them "don't get old." One of my friend's father he was passed away at 99, and he said every time when don't feel good, but don't tell me that is good, only tell, oh, my child, don't get old. And now I tell to my friend in library. Usually it's young people, I'm older of them. And no, you don't, you are young still. I know how old I am. But different how I feeling, yeah.

#### Q: Do you think the silent stroke was connected to any of your other medical conditions or anything related to your health? Anything in the past? Is there any connection between them?

#### A: I don't understand. You think that's my medication make me?

#### Q: No, I'm wondering if for you, in terms of how you think about this, do you think that the silent stroke was connected at all to other health issues that you might have?

#### A: I don't understand.

#### Q: Okay, that's all right. Let me ask you, you told me that when you learned about the diagnosis you were shocked. How else did you feel after that initial shock went away? What other feelings did you have?

#### [00:19:46]

#### A: Only think about that. And every time return– I try live healthy life about work, care about family, everything organized. I want that everybody's happy in house, prepare some surprise. But every time, back my last time because I came from country when was war and I think maybe many hard situation my body ruin. But then I was younger, before 20 and more years when started war, before 24 years war started. And I was stay alone in my city. I try only get out my husband and my daughters out. And I stay whole time there. I try. I think sometimes because I was health, very health. And my husband and I, our first year marriage, we try a health life. First about food, organize our job, our relics[?], later come children, care about them. Everything.

#### [00:21:07]

#### But I think on this time, I keep energy, that I survive war because they try me put out of my job. I stayed there, I fight with them. I didn't give that I lose job. And later they try that I leave my apartment and somebody take my apartment. I didn't. I have energy. I have energy for everything and when I left this country, when I came in America, I was under stress. I scare everybody on the street don't believe. There I didn't scare. Solve that with gun, with everything, nothing, I go. I didn't scare. But when I came here, I got stress and I scare when looking boy when play game with toy gun. I didn't get out from apartment. I tell to my husband.

#### And every Friday I was going to talk with doctor psychology at Tufts Medical Center, Andre Gargarian [?], very nice guy. He saved my life. And I talk every time with him about that, talk, talk, talk. And one day he said me that I go test for driver license. He said me that'd be good for me to relax. He said me, you'll sit in the car, slow drive, take music and enjoying around in America. Beautiful place when you look. And that's true.

#### [00:22:52]

#### And friend in library, friend which I met here, American friend and my people, I was better every day, every day. Sometimes I'm now depression. And when coming time I cry and spend with cry how I feel and go continue.

#### Q: Let me ask. I know you've told some of your friends about this, but how did you describe this news to your family and friends? When you left the hospital, how did you describe this to them?

#### A: What is this? Q: This meaning the silent stroke. How did you describe or explain that to your family or your friends?

#### [00:23:33]

#### A: Family knows, yeah. They know. And they little. But I think they try different face. "Mother, that is no nothing, that's silent stroke and it's passed. You don't think about it." They try me give me energy. That I think positive. And in library, too, yeah, they very surprised for that, send me card and tell that how they know me, that I will be winner of that. That I be winner for stroke, that stroke come back more.

#### Q: I don't understand that last part.

#### A: Yeah, they try, when send letter, card, that best wish and that I every day be better, better, better, and stroke stay on the back.

#### Q: I see.

#### A: Yeah. And they tell me, forget stroke. But stroke come back in my memory.

#### Q: So you had the hospitalization and actually you ended up seeing me in the clinic at a later time. Thinking back on the experience, how would you have wanted this to be explained to you? Was it okay how things were explained to you, or would you have preferred a different message or more information?

#### [00:25:06]

#### A: You think that did they good explain me about stroke? Yeah, that's good, normal. Yeah, very nice, yeah.

#### Q: In terms of how the information was presented to you though or explained to you, would you have wanted anything different?

#### [00:25:24]

#### A: No.

#### Q: No?

#### [00:25:25]

#### A: Normal. Yeah. I agree how they tell me. No, nothing much that I scare about that. Normal information. I need know, yeah, what's happen. That I, if I like myself and like life, I need to respect what doctor tell and try follow what is good that stroke don't come back.

#### Q: This is kind of an obvious question, but do you think that this silent stroke puts your health at risk?

#### A: Risk? I don't know.

#### Q: Okay. What I'm going to do is I'm actually going to give you a list of different symptoms or medical problems. And what I want you to do, this is just an opinion question, you don't have to know the answer to these. But I want to see how you think about this and see if you think that the silent stroke increases your risk or the likelihood that you might have one of these conditions that I'm mentioning. Okay? Do you understand that?

#### A: Something, but I scared that I don't– wrong understand.

#### Q: You definitely don't have to know the right answer. I just want to see just kind of explore your thought process.

#### [00:26:54]

#### A: Maybe I live here, here is a little different. When my family hear about that in ex-Yugoslavia, oh, they are so surprised and scared about that and don't believe that's happened with me because they know how I was healthy. What's happened.

#### Q: Let me ask about a few of these things. So for example, do you think that having the silent stroke puts you at risk for stroke with symptoms in the future?

#### [00:27:30]

#### A: I don't know, I don't know. I try don't think about that. I try, how everybody tell me to forget stroke, that "you didn't have stroke." That is therapy. [laughter]

#### Q: I appreciate you not necessarily wanting to think about it so much or sort of putting it behind you. But I'm wondering if you kind of step aside for a minute and just think a little bit about– let's say you were thinking about somebody else, a friend, who had a stroke but didn't have any symptoms, just like you had. Do you think that that person, if they had a silent stroke, would she be at risk for having another stroke?

#### [00:28:22]

#### A: Yeah, you remind me. I remember, I have friend in our country. She's doctor of psychology. Yeah, she finished her doctor. And she's a little younger of me. And I didn't talk, not yet, with her. I know that she first critic me how I live life. She will critic me. Please first don't worry, because she knows me, that I worry much about everything, everybody. Everything what happened there, everything what happen in America. I open news here, how be weather first I listen. So sad news about crime, killer and in my country, too.

#### [00:29:13]

#### And she said she had stroke. Silent stroke before. And she talk with my daughter more and tell everything what she tell to my daughter, my daughter tell me: Mother – her name is Ilyana – Ilyana had stroke, same, how– my daughter explained to her what's happened with me. Similar. And she said, everything be okay. She's very optimist person. She running. She work on the job sometimes have congress for psychology and pedagogy in Austria, Slovenia, go to Europe. Very active lady. She has nice, big house, weekend house and big garden. And she's active. And she tell to her mother need be only active more, active, running, running, running. And don't think about that. And she is good. She's good. She tell I didn't have stroke.

#### Q: So does that make you feel more hopeful–

#### A: Yeah.

#### Q: –seeing that she had the same experience–

#### A: Yeah. Yeah.

#### Q: –that she also had a silent stroke–

#### A: Yeah. Yeah.

#### Q: –and she's doing well?

#### [00:30:30]

#### A: And I didn't was still ready because I know that if she hear my voice which looking going to cry, she will critic me, be knowing me, and I don't yet, not yet call her. But of these days, I will call her and talk with her, yeah. She's very good person. She has many, many problems through war about family, children. And she survived. She was sick. Almost die. But she's winner about illness and about problem in life. And I need talk to her now.

#### Q: Are these changes that you've made in your life? Have you decided to be more active, physically active?

#### A: That I need more physical?

#### Q: That's what I'm wondering, if for you, if you've already decided to do that.

#### [00:31:25]

#### A: Yeah, I want a little of this time I feeling a little tired. Maybe this psychology. Depression make me tired. When I'm active, I feel more better. When I go work, everybody tell me "don't running, slow, you work slow." At home, I cook, I clean. When I feel tired, because I have problem with my vein, my leg, pain in leg, I quiet. But I think that is more better be active. Active, all time. Only while sleep, quiet.

#### Q: Did having the silent stroke convince you to be more active?

#### A: Yeah, yeah, yeah. Need more active, yeah. Need more active.

#### Q: Is there anything else that the silent stroke convinced you that you should be doing differently?

#### A: You think after silent stroke that I–

#### Q: I'm wondering if there's anything else you felt a need to change?

#### [00:32:29]

#### A: Yeah, need a little change because don't repeat. Yeah, more active.

#### Q: You're more active now. What else?

#### [00:32:38]

#### A: More active. More active house need, job need, family need. And make program when go to concert. I like music. I like go to theatre. And use this. And music in house. Then sing with grandchildren. Yeah, that is. And I feel more better. Yeah, when I'm quiet, I'm sick.

#### Q: I see. Have you changed anything else besides being more active?

#### [00:33:14]

#### A: I change now. I'm now more active.

#### Q: But is there anything else though besides being more active? Have you changed how you eat or the amount of sleep you get or medications?

#### [00:33:30]

#### A: Sleeping, one is sleeping is problem; I don't sleep much. But that's my personal. When I was young, I sleep about six hours, no more. And but over day I try, when I feeling ten minute sleeping, I sleep.

#### Q: Let me ask, so this is, I'm just going to mention a few things about the current status of what doctors know at this point in time in terms of the entire field of medicine. Oftentimes what we do is we do research studies and we see when somebody has a medical problem what type of things happen later. And if we have a good number of studies and have observed a lot of people, then sometimes we can develop guidelines, which are just general practices that we recommend for most people. For stroke, there are a lot of guidelines in terms of preventing a first-time stroke or preventing a stroke, a recurrent one, after having a first one.

#### But usually we think of stroke as being something where a part of the brain gets injured and somebody has symptoms. So having a stroke and not having symptoms is a little bit of a different entity. So some people extrapolate or think, okay, we can treat it like stroke, and some people think maybe we shouldn't be, maybe it's actually something that's different. And so, currently there are no national guidelines or international guidelines to say after having a silent stroke, this is what we should do – one, two, three – in terms of steps.

#### So does that concern you at all? Does that bother you, that there are not currently guidelines helping doctors figure out what to do for patients like you?

#### [00:35:51]

#### A: I don't know if I everything understand. But I tell only everybody we need believe doctor, what doctor tell, what suggest, because doctor has experience and follow everything. Sometimes I think when I watch on TV that is stories, something about people which try make healthy, stop pills, and eat only, drink tea and eat fresh plants. That's crazy. I can't be. [laughter] But I don't know, did I– general I understand what you tell. But I tell only every time, when I talk with my friend, if somebody's illness, sometimes we don't good patient, we don't listen. But we need listen and respect what doctor tell.

#### Q: How important is it to you that we learn more about silent strokes, that we research this or study this? Is that important to you?

#### [00:37:14]

#### A: Yeah, important is, yeah. Because for every patient is one more experience. Is that? Every patient, we don't say[?] my silent stroke of another, no say, something is different. Body's different. And every time is something new. You find something new. Yeah, doctor, yeah.

#### Q: We're hoping to do studies in the future where we can see when somebody has a silent stroke, what's the best way of preventing another one or preventing a stroke with symptoms. If we were to do those studies and we found that there's a particular treatment – say it's something like Plavix or aspirin or something else, or some other medication or maybe increasing activity – let's say that we found out that actually helps a little bit, would knowing that there's a little bit of a benefit be enough to motivate you to do that, to be more active or take a medication?

#### [00:38:29]

#### A: I don't know what is best. Only I think of these days that I try one or two weeks only drink juice, nothing more, juice of vegetable, of herb. Nothing. Nothing pills, nothing. I think about that. I think about that's be body clean of everything what is make worse body. Because if I eat fish, maybe fish, yeah, somebody tell fish is healthier. I don't know what is of meat healthiest. People which vegan, they think that is healthiest food. No meat, no nothing. And I think about that, that be good.

#### [00:39:16]

#### Because I watch one guy. I tell when they diagnose that he has cancer of kidney, and prepare him from surgery, that is his choice, he tell "no, no surgery." And he left city, go to live in wood. First week, drink only water. And he said that was hard time. Because he feeling hungry. And second week, he drank continue again water. He felt better because hunger go low because body adaptation new situation. And step by step, he started only what he found in the wood, of herb, of some plants. And when he was going continue, I watch guy on TV, he can't lie, he talk true. And he goes on control, they didn't find nothing. Cancer gone. And sometimes I think about that, only drink some juice drink and nothing more eat.

#### Q: You should probably have a more balanced diet besides that.

#### A: I don't know, I don't know.

#### Q: Let me ask. We're almost at the end of the interview. After having talked about this for a half an hour, have any your thoughts changed in terms of silent stroke?

#### [00:40:53]

#### A: For now? No. We talk and I try, all time I try understand that's happened, that's my body. That doesn't matter how I– because sometimes everybody tell, "oh, how happens, you are family which worry about healthy food all time." Because everybody think about food. But except food have something in other life, life which make stress, every problem. And I think that maybe every stress what I have, every day I have stress for something. And make me strong.

#### Q: What does it mean to you to have a stroke?

#### A: What does it mean? Q: Yeah, in terms of hearing that you had a stroke, what is its impact on you?

#### [00:41:56]

#### A: I don't know. That's happen. I try think. I try think about my vein, artery, what's work as in factory. How work and how coming, what coming and make some as clot, air clot. And I try think as machine, how body is robot and what's happen, where coming in the brain and coming push on some capulars and my trunk[?] I try think about that. And every day usually I tell my family, one week I will start only drink, that I see what will be with my circulation. Drink water and drink juice, nothing more.

#### Q: I think you should have something to eat as well.

#### A: Yeah, I know. I know that I need protein and other what the body.

#### Q: Do you have any questions or other things that you would want to say?

#### [00:43:09]

#### A: Only question. I don't know that's back some stroke and usually how long time usually if something back – after six months or some.

#### Q: Let me see if I can rephrase that. Are you wondering how soon stroke can happen again?

#### A: Yeah, yeah.

#### Q: Okay. We can talk more about that. That's I think an important question. Anything else on your mind? Or anything else that you would want to ask me?

#### [00:43:50]

#### A: No. Only that. Because I know that my mother had one and coming another 24 hours. But here it happened, five years, she lived normal life. She started walk as baby, we learned to her walk and she lived five years after stroke. And I don't know, did I tell you, I think about that same day when she got stroke, 24 April. After five years, on 24 April, she passed away. Same day when got stroke she passed away after five years. Only I think about that.

#### Q: I'll stop the recording right now.

#### A: Okay, thank you.

#### Q: Thank you.

#### END INTERVIEW

**Participant 9**

#### Q: Okay, so this is the interview with our ninth patient. Can you say hello?

#### A: Hello.

#### Q: Great, I think that should be okay. So the first question is very open-ended. It's just to have you tell me what you know about your diagnosis.

#### [00:00:16]

#### A: Well, I know very little except I had an MRI of my brain. And Dr. R in neurosurgery saw a white spot. And he said that was a TIA. And he didn't recommend that I see a neurologist, but I just thought I would.

#### Q: Okay, fair enough. What were you see Dr. R for?

#### [00:00:44]

#### A: Well, I have some problems in my lower lumbar area and I have a pinched nerve and I have a mass. And I'm having an MRI on Saturday to see if that mass has changed. And then I'm seeing him on Monday. So my concern about TIAs is that my mother had TIAs and she then eventually had a stroke. And she was unable to speak, and it took a year for her to die. And it was a terrible situation. So that's, I'm concerned about– and I know many people have TIAs and it's not necessarily fatal, but. So that's really all I have to say about it.

#### Q: Fair enough. And so this was a head MRI, a brain MRI was obtained by Riesenburger? A: Yes.

#### Q: In the setting of having this pain–

#### [00:01:41]

#### A: I had an MRI of my entire– my brain, my thoracic, my whatever this area is called–

#### Q: Spine.

#### [00:01:49]

#### A: –and my lumbar area.

#### Q: So it was part of that series–

#### A: Yes.

#### Q: –that you got this scan. And then he found his finding on the scan and let you know about that.

#### [00:01:57]

#### A: Yes, he showed me the little white spot.

#### Q: Besides mentioning that this was a TIA, did he say anything else about it?

#### A: No, he didn't. He said– no, he didn't say another thing.

#### Q: Okay, fair enough. Did he refer to it in any particular way in terms of descriptors? Did he say it was an unexpected finding or incidental finding?

#### [00:02:23]

#### A: No, he didn't. He said, actually he wasn't totally absolute that it was a TIA, but he said it could be. And that's all he said. I mean, he didn't suggest that I go and see anyone else about it. He didn't seem that concerned.

#### Q: When you heard that information, did you connect the symptoms that you went to him for, this pain, to that finding? Or did they seem separate?

#### [00:02:57]

#### A: No, I thought it was totally different.

#### Q: So he didn't suggest that you go see anybody, but did he give you any advice on things to do?

#### A: No.

#### Q: Okay, not particularly, okay. You mentioned that he wasn't absolute in terms of saying this is a TIA. Do you remember the words that he used to describe that? You said he called it a TIA, but wasn't absolute.

#### A: Yes.

#### Q: Did he say "this could be" or "I'm not sure, this might be"?

#### [00:03:28]

#### A: No, he didn't say I'm not sure. He may have said– I don't remember. He may have said "I think this is a TIA."

#### Q: Okay. How concerned did he seem to be about that finding, when he was relaying that information to you?

#### A: Not very concerned.

#### Q: Okay, so it sounded like your concern came up afterwards when you heard.

#### A: It's only because of my mother's situation that I was concerned.

#### Q: Fair enough. So afterwards, I know you saw Dr. T who's a stroke neurologist.

#### A: Yes.

#### Q: Did you go straight to him as a referral? Or did you end up talking to your primary care doctor first, or somebody else who made the referral for you?

#### [00:04:15]

#### A: I talked to my primary care physician. And they suggested, or she suggested that I come here. But her office was not able to get an appointment for me. Since I used to work with TR, she was able to get an appointment for me immediately. So that's how I came to see Dr. T. And then, when I was talking to him, I told him that I had never had taken an aspirin because I bleed easily. And he suggested that I should try to take an 82-milligram aspirin and see how it went. And that's what I've been doing since I saw him, which is about, I don't know, a month-and-a-half ago, I'm not sure.

#### [00:05:11]

#### And he didn't seem that concerned either. But he suggested that maybe I talk to you and join hit study.

#### Q: Fair enough. Let me take one more step back. Between seeing Dr. T and Dr. R, the surgeon, you had a conversation with your primary care doctor. What was that conversation like?

#### [00:05:32]

#### A: It wasn't very long, because she said I should see a neurologist. And she wasn't a specialist in strokes. So that's what I did. There wasn't much to that conversation.

#### Q: Fair enough. I'm not suggesting that there should have been.

#### A: Right, it's okay.

#### Q: I'm just curious to see how the discussion has evolved over time. So for Dr. T, the stroke neurologist who saw you, when he looked at the images and talked to you about this, how did he present the findings to you?

#### A: Well, he wasn't even sure it's a TIA.

#### Q: What sort of words did he use to–

#### [00:06:26]

#### A: Well, I think he said something like, "Well, it could be, but not necessarily. It could be something else." He didn't seem terribly concerned. [laughter]

#### Q: Okay, fair enough. And it sounds like he did give you some advice. He asked you to take an aspirin.

#### A: Yes.

#### Q: How certain did he seem to be about the advice that he gave to you?

#### [00:06:55]

#### A: Well, certain enough that I decided I would give it a try. I mean, he didn't think it was going to do any harm. Because the reason I haven't been taking aspirin, I mean, I know everyone takes an aspirin at my age, was really because of that bleeding situation. So that's why he suggested I give it a try and see how things went. I mean, he didn't seem overly concerned about the whole thing. He just thought it was a good idea.

#### Q: I do want to ask you about the bleeding question afterwards. With this conversation with Dr. T, is there anything that he emphasized to you or felt was an important message to convey?

#### [00:07:43]

#### A: Well, he did all these little tests and he said everything seemed to be all right. He didn't seem to think that I needed to see him again. And so, there isn't any sort of major thing that sticks out from that conversation.

#### Q: Fair enough. So it sounds like he expressed some doubt about, or some uncertainty as to whether or not the finding on the scan was a TIA or a stroke.

#### A: Right.

#### Q: Did he express any doubt about anything else?

#### [00:08:23]

#### A: No.

#### Q: That was the only thing?

#### [00:08:24] A: Yeah.

#### Q: Taking a step back and thinking about the interactions you've had with the neurosurgeon, your primary doctor and the neurologist, and maybe just thinking in general about kind of the world and how people might experience or approach this, what would your opinion in terms of how well you think doctors understand this diagnosis or this issue that you have?

#### [00:08:58]

#### A: Well, I mean, I assume that [laughter] they know what they're talking about. So I don't have any, I don't feel uncertain about or I didn't really question, well, gosh, are you sure that it may not be a, you know. I didn't really have any sort of doubt about it. I just went along with whatever Dr. R said because, well, what else am I going to do? I need– and the thing is, my lumbar issues really much more of a concern to me than the TIA. Because it's painful. And I don't experience any–

#### Oh, one of the things that Dr. R asked me is if I could remember any event that happened that may have caused this TIA. And I racked my brain, and I did have one, I forget exactly when it was, a year or a year-and-a-half ago. I had an event. I had lichen planus on the inside of my cheek, and someone did a biopsy on it. This is all at this hospital. And a few days later, I felt very dizzy and strange. And I came into the ER and they observed me for a while. And I think, but I'm not sure, I had a CAT scan. But no one saw anything on it.

#### [00:10:57]

#### And so, maybe CAT scans don't show that. Maybe only MRIs do. Because I was trying to think when this particular event might have been that I had this thing. So that's the only thing I could think about. I don't remember any sort of weakness in my arms or any, you know. My speech was kind of slurred because of this biopsy, my cheek. But actually, it was after several days of the biopsy that my speech became slurred. So that was really the reason I decided to come to the ER. Cs I thought maybe I was having a heart attack or something, that's why. But there seemed to be nothing wrong with me. I mean, it's somewhere in my records, so you know.

#### Q: How long ago was that?

#### [00:11:52]

#### A: Well, it's less than two years ago. And it's in, I mean, someone saw it, in my electronic record. So if you wanted to, you could look for it.

#### Q: Sure, sure. Did either of these three doctors use other terms besides TIA for this? It sounds like you didn't have any clear symptoms that you could connect to this finding, but did they call it a silent stroke or an asymptomatic stroke or something like that?

#### A: No. They just, the only word that was used, that Dr. R used was the TIA.

#### Q: And your primary care doctor and Dr. T, did they use any different words?

#### A: No.

#### Q: Taking a step back in terms of when you first heard this information and up to now, how did learning about this make you feel?

#### [00:12:58]

#### A: Well, I didn't respond– I wasn't terribly concerned. But I was a little bit concerned just because of the history in my family. So I wanted to find out as much about it as I could and if there was something I could do, like take an aspirin a day. [laughter] You know. Then I was happy to do that.

#### Q: You're referring to that in past tense. Has that changed over time, the level of concern? Has the concerned lessened or has it increased or stayed the same? [00:13:30]

#### A: No, I don't think about it really very much.

#### Q: Fair enough. In terms of the concern that you had when you did learn about this, what were your concerns?

#### [00:13:47]

#### A: Well, my concern, I was, I related it directly to my mother when she had her stroke. And what a dreadful year she had before she died. That she couldn't speak. She was just, her life was miserable.

#### Q: I'm sorry to hear that.

#### A: Yeah.

#### Q: It sounds like hopefully your situation might be different, at least in terms of the way this is presenting. But hearing about this, how bad do you think this is for your health, having this silent stroke, or whatever one calls it, TIA or something on the scan.

#### [00:14:37]

#### A: Well, I don't really know. I mean, I hear that there are people who have many TIAs and they get through life okay; they don't all necessarily end in having a major stroke. So I'm not really that worried about it.

#### Q: You kind of alluded to this before, but thinking about all of your health issues, how does this rank in priority amongst those health issues?

#### [00:15:08]

#### A: Oh, my highest priority now is to deal with my lumbar issue. Because that involves pain. And I've had several shots of cortisone and I don't think that's so wonderful. But I hope that somehow we can figure out what to do, because I don't want to spend the rest of my life being so uncomfortable. So that's my main, my prior, my most important concern.

#### My other issue is that I had a partial nephrectomy last year. But everything seems to be okay with my kidney. And it was outside the kidney. So I think that I've had CAT scans and X-rays of my lungs. Or, no– yeah. A CAT scan, actually. So I have that at the Brigham, and I'm being followed there. So I think those are really the only two issues.

#### [00:16:19]

#### But I'm not sort of spending a lot of time thinking about it. It doesn't invade my life.

#### Q: This particular issue.

#### [00:16:27]

#### A: Right. Well, any of these issues. I mean, I just, I'm aware of it in the back of my mind, but it doesn't consume me at all.

#### Q: That's important to know. So people refer to these in a number of different ways. Usually we reserve the term TIA for when somebody has symptoms but there's no evidence of injury to the brain on a scan. We refer to strokes most of the time when somebody has some evidence of a brain injury on the scan and symptoms that connect to it. There are a number of different names, but sometimes we refer to something as being a silent stroke if we look at a scan, see something that we think is most likely due to the same mechanism that causes stroke or something similar, but there's no clear symptom or episode that's connected to that. So that's we call it silent or asymptomatic. That's just a little background.

#### So now that you've been told that you have this, this finding on the scan, which we sometimes will call silent stroke, do you think this might cause other bad things for your health?

#### [00:17:40]

#### A: No.

#### Q: Not really? You don't think it has any implications for what might happen in the future?

#### [00:17:46]

#### A: Well, I mean, it has crossed my mind that I, you know. I'm 78. I may have more of these. And I just keep hoping that I'm not going to have a stroke.

#### Q: Sure. When you say more of these, you mean more–

#### A: TIAs.

#### Q: –evidence of injury to the brain without symptoms? Or do you mean you're worried about having brain injury with symptoms?

#### [00:18:12]

#### A: Oh. Well, I haven't really thought about it in those terms.

#### Q: Sure. Maybe I'm putting words in your mouth though, but it sounded like knowing about your mother's experience, that you're worried about something that might cause disability or illness or an early death. Is that fair to say or is that incorrect?

#### [00:18:36]

#### A: Yeah, that's correct. But as I said, it's not something that I think about every moment of the day. It's just something that I'm aware of, and I hope is not going to happen.

#### Q: So let me ask, just to clarify, in your opinion, do you think that having a silent stroke, something on the scan that indicates some prior injury in the past, do you think that puts you at risk for having a stroke with symptoms in the future?

#### [00:19:10]

#### A: I think it's possible, yes.

#### Q: Are there any other problems that you think might come from this?

#### [00:19:19]

#### A: No. Like being forgetful? No. [laughter] Dr. R asked– oh, yeah, when he asked me if I could remember any event that may have triggered this, and I said, Well, I have a difficult time remembering names, but I assume that's part of my age. I don't relate it to any sort of specific event.

#### Q: Sure, sure. That's a common symptom.

#### [00:19:44]

#### A: Yeah, exactly. So I don't feel, yes, that I'm losing it. But maybe I will. [laughter]

#### Q: Do you think this is connected at all to any of your other health issues? And if so, which ones?

#### A: No, I don't.

#### Q: How did you describe this to your family and friends, if you did?

#### [00:20:08]

#### A: I said that I might have had a TIA. That's basically it. I didn't make a big deal out of it.

#### Q: How did they respond to that?

#### [00:20:21]

#### A: Well, gee, they weren't overly concerned. [laughter] No, I mean, because I didn't present it as a scary thing. I just sort of– because even Dr. T said, he didn't say 100% that it was a TIA. And he said at my age, one often finds little white dots in the brain on MRIs. But I just latched on to the TIA because that's something I know about. And so.

#### Q: If one of these doctors had presented it to you in a way that seemed like they were very concerned, would that have changed how you approach this or thought about this? Would it have affected your level of concern or your approach?

#### [00:21:25]

#### A: Well, I would think so. I mean, if Dr. T had said, "Oh, gosh, now we have to really think about a very serious event taking place," I mean, I don't know. I would have been very concerned and I would be thinking about it all the time, wondering what there was that I could do to prevent it. I don't know what else to say, that's about it.

#### Q: No, no, that makes perfect sense. Did you describe this to any of your other doctors, and if so, how did you do that?

#### A: No, I only described it to my primary care physician.

#### Q: How would you have wanted this imaging finding, this test finding to explained to you? Was it explained to you in a way that you thought was good, or are there things you would have preferred?

#### [00:22:30]

#### A: Well, it wasn't a big deal. He showed me all the MRIs of my body and he just said, "Oh, look, there's, look at this white spot here. I think that's a TIA." But he didn't seem overly concerned about it, because he said, "Well, if you want to, you can go and see"– because I said, "Well, what should I do about it?" And he said, "Well, if you want to, you can go and see a neurologist." Didn't say, "Oh, you have to go and see a neurologist."

#### Q: This was the neurosurgeon?

#### A: Yeah, exactly.

#### Q: At this point in time, I know you've mentioned at least one thing, but do you think you will change anything now that you've learned about this?

#### A: No. I'm trying to do more exercise. [laughter]

#### Q: Why is that?

#### [00:23:26]

#### A: I don't know, because the thing is that I was very good about doing exercise until I had this pinched nerve. And I felt I was not living a healthy life, so I forced myself to swim every other day at least, if not every day. And so, I think that I'm hoping that that's a good thing. Because I still cannot do the sort of exercises that I have been doing for several years because of this pinched nerve situation. It really invades my life.

#### Q: Just to clarify, are you wanting to exercise more partly as a result of hearing about the silent stroke? Or are you wanting to exercise more independent of that?

#### [00:24:15]

#### A: I think it sort of made me realize that it's really important, I mean, that I have to continue to do something. And so, that's why I decided, well, since I can't do the other thing, swimming is a very good exercise, and it's not painful to do, really, compared to sitting on a bike or lifting weights or whatever. You know, so.

#### Q: Are there other things besides exercise that you want to change?

#### [00:24:48]

#### A: No. I don't drink very much, and I'm not changing that.

#### Q: And you mentioned Dr. T suggested you take an aspirin. Is this something that you've started doing?

#### A: Yes, ever since I saw him, which I forget when exactly that was. But I take one every day.

#### Q: How committed do you feel to that idea?

#### [00:25:10]

#### A: Oh, that's fine with me. I mean, I haven't had any negative results from it.

#### Q: You did mention concerns about bleeding. Is that correct?

#### [00:25:19]

#### A: Yes, because I used to– well, for many years I was told– because if I have any sort of little injury, I do bleed quite a lot. But I have to admit, actually, that it's not as bad as it used to be, because I used to take a huge amount of Excedrin because I kind of suffered from horrible headaches. And I think that had something to do with my bleeding issue. But I mean, I don't have that problem anymore.

#### Q: Were these migraine headaches?

#### A: Yes, kind of. It was never really diagnosed as migraines, but it was horrible anyway.

#### Q: Do you think there's any uncertainty or lack of clarity about how to deal with this particular issue?

#### [00:26:19]

#### A: Well, yeah. It's not exactly– I mean, I haven't been told that there are five steps that I should take to deal with it. No. I just, you know, have to wait and see what happens. But I don't really know what to do. I mean, that's why I sent to see Dr. T, to see, well, is there– and all we came up with is that I should take an aspirin.

#### Q: Is there some feeling like you think there should have been more steps?

#### [00:26:59]

#### A: No, I don't. I mean, if he thought that, he knows what he's talking about, so. [laughter]

#### Q: How urgent is it for you to do something about this? It sounded like you mentioned that this is not a major concern for you.

#### A: No.

#### Q: But do you feel like there's any urgency to make changes or to take any actions?

#### [00:27:22]

#### A: No, because I don't think there are any actions.

#### Q: Fair enough. Let me mention a couple things to you as we're moving towards the end of the interview, and I'll tell you a little bit about what the field knows so far. There are different types of guidelines. So medical societies in primary care, in neurology, in cardiology and so on will set up guidelines to help the whole field of doctors and nurses, whomever, give advice to patients to prevent different conditions. And so, these are often grouped into two categories. There's something that's called primary prevention, which is to say for people who are healthy or don't really have a heart attack or a stroke or anything like that, we'll tell them to do certain things in order to prevent a heart attack or a stroke. And then if somebody's had a stroke, there are things that we advise them to do to prevent another one from happening.

#### So this is a little bit of an in-between situation in some ways, in that we're discovering that some people have what we think could be, or maybe we are certain, are strokes on a scan, but there are no symptoms connected to it. So it's a little bit of a slightly different situation. And there are actually no specific guidelines for that situation on a national or an international level. There are no specific research studies that guide physicians in selecting tests or therapies or other measures when somebody's discovered to have a silent stroke.

#### So hearing that, does that change how concerned you would be about this particular issue or having one of these?

#### [00:29:15]

#### A: No, it doesn't.

#### Q: It doesn't, okay. It doesn't make you more or less concerned than before.

#### [00:29:21]

#### A: No. The thing is, I mean, no one– when you tell me that there are these guidelines, I mean, then I sort of thought, well, no one has given me any of these guidelines, except the suggestion of taking the aspirin.

#### Q: Right. So just to be clear, for this particular situation, in terms of having a silent stroke, there are *not* guidelines for that.

#### A: Okay.

#### Q: So there's actually some variability. There's some heterogeneity in terms of how different specialists, different primary care physicians, different surgeons, whomever, there are different approaches that people will take to this issue for individual patients. Does that affect actually how you perceive or trust the advice given to you by your doctors?

#### [00:30:21]

#### A: No. I don't think.

#### Q: Not particularly? A: No.

#### Q: You still trust that they're doing–

#### A: Yes.

#### Q: –the best by you in terms of their guidance.

#### [00:30:28]

#### A: Yes, I do. So far.

#### Q: How important would it be for you that this be studied more or that people try and figure out more about this?

#### [00:30:39]

#### A: I think it's very useful. I mean, because if you find out something that could help people either prevent having these things or help them get, I mean, you know, if I have another one of these and things get worse, I mean, I'd like, if there's something people figure out that can be done about it, obviously I think it would be a good idea.

#### Q: Extending that thought a little further, let's say we are able to do studies and we find some health benefits, even if they're relatively small, whether it's a lifestyle change, like doing more exercise, or taking a medication like aspirin, would you be motivated enough by a study that finds even just a little benefit to take another pill or change your lifestyle?

#### [00:31:31]

#### A: Yes, I would.

#### Q: So you think it would be important enough [simultaneous conversation]

#### A: Yeah, I do.

#### Q: So we're actually pretty much done with the interview. I was just going to ask, after having gone through this discussion, if your thoughts or perceptions on silent strokes have changed. And if so, how?

#### [00:31:52]

#### A: No, I don't think they have. I mean, I feel as though I'm sort of taking it very lightly. I think that I've done as much as I can to find out about it. And you know, if there's some new information that comes up about any thing I can do, then I'll be happy to do that.

#### Q: To hear about it and do that.

#### [00:32:19]

#### A: Yes. But I feel that I've done everything I possibly can to find out about the situation. And I will just carry on. If something new comes up, then I'll be happy to, you know, to–

#### Q: I'll stop the recording right after this, but do you have any particular questions or thoughts on your mind, things that are important for you to find out?

#### [00:32:54]

#### A: No, I don't think so. I think we've covered things.

#### Q: For future studies, do you think there are any questions that you would want to ask or want to know the answer to?

#### [00:33:05]

#### A: Well, I mean, if you are doing a study and you find out something concrete, I'd like to hear about it.

#### Q: Okay, fair enough. Sounds good. I'll stop the recording right now.

#### END INTERVIEW

**Participant 10**

**Q:** Okay, so we're going to get started with the interview. This is our tenth patient. Can you say hello?

[00:00:07]

**A:** Hello.

**Q:** Great. So, these are all open-ended questions, and I might ask you a few follow-up questions just to explore your thoughts a bit more. So again, the study is about findings on brain scans that might look like strokes, usually without symptoms or any sort of over-connection to what people presented with initially. And so the first question I have for you is to just have you tell me a little bit about what you know about the imaging findings or about your diagnosis?

[00:00:37]

**A:** Well, I was caught unawares when the word stroke came up, and it provoked a response from my primary care physician and she was concerned, and I was too because I don't know if these things have-- if there have been many, just you know, a lot of them that I didn't know they were a stroke. I really don't know the symptoms and what not, so maybe I could have had one and not even realized it.

**Q:** Who do you identify as the person who kind of first mentioned this to you or who gave the diagnosis to you?

**A:** Of the stroke?

**Q:** Yeah?

[00:01:24]

**A:** I believe it was Dr. S. I may be wrong about that, but I think it was Doctor-- it was somebody here who brought up the possibility. Then I was referred to seeing you.

**Q:** And so Dr. S is one our headache specialists, one of our neurologists. What were the circumstances under which you saw him?

[00:01:51]

**A:** I was having a problem with my left hand just grasping; dropping things that I'd never done before. So, when I came in, they gave me some tests and what not, and his finding was to move me on to see you to see if there was something else that he was unaware of or missing or a second set of eyes just to see.

**Q:** So did Dr. S order the brain scan or was it your primary doctor that ordered the brain scan?

**A:** Dr. S.

**Q:** And what did he explain to you about the test at the time?

[00:02:33]

**A:** He was just looking for any type of-- the reason for me having this test was to see if there was any abnormality or something that could indicate why I was having this problem with my hand. Nothing really specific, it’s just one of those let’s take a test and see what happens.

**Q:** Sure. Did he mention the possibility of stroke at that time, or he did not mention that necessarily?

**A:** I don't believe he mentioned stroke. I think he said that some of the testing that he was doing was uneventful, so he wanted to further it. But I don't think he did because I definitely would have-- but when I had a meeting with my primary care physician and then I guess whatever transcript was in the data, it was like whoa.

**Q:** Okay, so it was your primary care doctor who presented the term stroke to you?

**A:** Yes.

**Q:** And how did your primary doctor describe that to you, or sort of what wording or description did he or she provide?

[00:03:49]

**A:** She was somewhat taken aback, I think. And she had been my primary care for a while and I took by her demeanor there was a concern there. It was something that maybe she wasn't expecting. And that triggered a series of events that got me here.

**Q:** Sure. Did she use the term unexpected or incidental? Or was it more her reaction?

[00:04:28]

**A:** It was more-- I picked it up as more of her reaction.

**Q:** And that reaction of surprise is what sort of translated to you as being an expression of concern about stroke?

**A:** Oh definitely, yeah.

**Q:** So, when you observed that reaction of her being surprised and concerned, what sort of impact did that have on you in terms of your thought process?

[00:04:54]

**A:** None.

**Q:** It didn't?

[00:04:56]

**A:** No. It is what it is. I mean, you take-- tests define certain things, and if something else comes up from that test, you live with it. So it wasn't--

**Q:** So it didn't make you anxious or surprised or scared or anything like that?

**A:** No.

**Q:** You were just kind of like, okay, well this is a new piece of information, let's see where it goes?

[00:05:16]

**A:** Yeah. More data would have been-- okay, now you're going in this direction. What do we do now?

**Q:** Fair enough. So you mentioned that you had these symptoms with your hands. And I'm going to ask you some questions as if I hadn't talked to you before about this. But I'd just like to talk a little bit about those symptoms that first brought you to your primary doctor and then to Dr. S, about the left hand.

[00:05:44]

**A:** I'd be grabbing a dish towel, or I'd be holding a pen or screwdriver at work and then it would just-- I felt that I had it, but it just released. Clumsy, okay? Well, then it kept happening a little bit more frequently. And my wife noticed it one time, we were in the kitchen, and I just reached for the towel and I turned and it hit the floor. I'm saying, “This is-- this isn't normal. I'm very sure-handed.” I believe I'm ambidextrous, I might even be left-handed, but it concerned me that there's something causing this outside of, you know, forgetfulness. I mean, you shouldn’t have to remember how to grip a towel, but that's one of the things that lead me to say is there something wrong?

I'm not hypochondriac, I don't have tests for a hangnail or a blemish. But I just-- this here kind of concerned me.

**Q:** Before you went to see your primary doctor, or Dr. S, did you have any theories or any sort of initial guesses as to what might cause those symptoms?

[00:07:03]

**A:** No. No, there was-- I never really gave it much of a thought because I didn't want to dote on the fact it could be something wrong. I was just going to say let's see what the professionals say and deal with it that way. I'm not really big on the what ifs and what, you know, what could be something. You'd drive yourself crazy.

**Q:** Yeah. And then now that you've gone through these evaluations with your primary doctor, Dr. S and me as a stroke neurologist, do you think there's a connection between those original symptoms and the diagnosis of a silent stroke or the findings on the brain scans?

[00:07:54]

**A:** If you say there is a connection, or if you believe there's a connection, you are far more versed in it than I am. I really don't know how-- I'm not aware of what stroke is. I mean, just a very-- what it causes. But, my impression for stroke was you're struggling-- you're useless. I mean, you lose all ability to use a certain part of your body. Seeing it was just I couldn’t pick something up, the severity of strokes never really entered my mind.

**Q:** Okay. When your primary doctor first mentioned this to you, looking at Dr. S report, mentioned the possibility of stroke, what sort of advice did she give to you?

[00:08:48]

**A:** To have additional testing, let’s see what's-- like I say, she's my primary care so she said let's go with the pros.

**Q:** Okay. Did she talk about any particular tests?

**A:** No. She just said, “Let’s deal with someone who it’s their specialty.”

**Q:** Okay.

**A:** Similar to if I have a bad back, she sends me to this person.

**Q:** Fair enough. And she ended up sending you back here for the second evaluation with me in terms of a neuralgia evaluation?

[00:09:30]

**A:** The evaluation with you was initiated by Dr. S. I just kept my doctor in the loop. I see her like four times a year.

**Q:** So that plan was already set in place and--

**A:** It was already set in place.

**Q:** --mentioned to her and--

[00:09:41]

**A:** She was just making-- she's waiting, it was waiting on the results the same as I was.

**Q:** Okay. Out of curiosity, did she imply to you that there were definitely going to be new tests done, or did she say let's see what the stroke doctor has to say?

[00:10:01]

**A:** Let’s see what-- let’s see what we've already found.

**Q:** Okay. Is there anything-- actually, in terms of that advice, how certain did she seem to be as she was giving that advice to you? Was she saying, “Yes, this is definitely what you should do,” or was she saying, “Oh, this is probably something you should do, or I'm not entirely sure what's the right thing to do here. But you might as well go see the stroke specialist?”

**A:** Well, the relationship I have with her is that she doesn't have to prod me on something like that. I trust her very, very much.

**Q:** Great.

**A:** And I have a great deal of respect for her. And so knowing the type of person I am, she doesn't, you know, harp on something. She’ll make a recommendation and I trust her implicitly. “If that's what you want me to do, I'll do it.”

**Q:** Great. Is there anything that she emphasized after that visit or during that visit?

[00:11:06]

**A:** No, not really. Just let’s see what happens and we’ll-- once we have everything we need to-- once we have as much as we get, then we’ll deal with it.

**Q:** Sure. Did she express any doubt about anything?

[00:11:27]

**A:** Like I say, I think she was a little bit surprised when the word stroke was mentioned. I mean, I have hypertension, my blood pressure does run high. I'm taking medication for that. I have been to cardiologists here at Tufts and they have given me medication for it. So, stroke really wasn't-- I wouldn't say she wasn't concerned, wasn't something that she would harp on or go to that stroke, because it’s just maintaining my blood pressure.

**Q:** Sure. So, it is a little bit of a new concept for a lot of people, this idea that stroke or what the medical term for it is is brain infarction, kind of like myocardial infarction is the medical term of heart attack. It’s kind of a new concept for a lot of people that this can occur without symptoms, really without sort of overt symptoms. And in general, reflecting on the experiences you've had with three doctors and perhaps others, just from your opinion, how well do you think doctors understand this diagnosis or this process?

[00:12:55]

**A:** I really don't know because when I had heart problems back then, you just dealt with it. I mean, I'm not sure what they should know. I'm not sure how comfortable any doctor is with something as specialized as a stroke.

**Q:** Fair enough. So then you eventually came to see me and we talked a little bit about that and we talked about your symptoms, as well as the findings on the scan. And this is not meant to be a quiz, but do you recall any of the sort of messages that I gave to you at that visit or anything that I emphasized for you?

[00:13:47]

**A:** One of the spots you saw was-- well, I asked you if this could have been caused by head trauma. I've taken a couple of shots to the head and you said no. That this was-- could be from-- I believe it was cholesterol?

**Q:** I think cholesterol or blood pressure, I'll have to look back in the record and see.

[00:14:11]

**A:** Because I asked you and you said no, because there would have been something on the side of the head, but these spots were in a certain location that it was definitely what I was thinking could have--

**Q:** And did I seem to connect the symptoms that you had, the left hand, to any of the spots on the scan?

[00:14:34]

**A:** Not that I remember. A lot of times when I see a doctor, he tells me what he thinks, and well, that's why I trust you. I don't really question their thing. I mean, if I get a cut on my hand and he gives me an aspirin and says, “Take two and see him in the morning,” well then wait a second. You know, are you going to stitch this or not?

**Q:** Right, sure. Fair enough. So, now thinking back on the past several weeks, past couple months, and thinking about being given this diagnosis of silent stroke, how does learning about this make you feel just in terms of your overall response?

[00:15:30]

**A:** Well, it gives you an awareness of dos and don'ts. Just basically a wakeup call to take care of yourself better.

**Q:** Okay. So you're sort of seeing it as an opportunity of as oh, okay, well I have this--

**A:** It's a learning experience. I mean, I don't delve into it to find all of the little minutiae of what's going on because that would drive me crazy.

**Q:** Okay, fair enough.

[00:15:58]

**A:** I mean, I got something, I got something. I don't have to know-- I don't need the exactitude of what everything is because I could get hit by the train on the way home. So I don't really--

**Q:** Need to dwell on that necessarily?

**A:** Right.

**Q:** Okay. Do you have particular concerns about having this diagnosis?

[00:16:18]

**A:** No.

**Q:** No? Okay. You just sort of are not dwelling it. Know that you have it and you're sort of developing a plan to address that? If I can have you reflect on that just a little bit, how bad do you think having this is for your health?

**A:** For my health?

**Q:** Yeah?

[00:16:43]

**A:** I have interstitial cystitis and I've had it for a while. There's nothing they can do for it, they tried everything. My thought process is with the stroke, cause for stroke, I have enough-- I have enough other problems that this one here, even though it is-- could be life threatening, okay, my quality of life is fine and so I don't-- it hasn’t really altered how I see anything. I have enough-- I'm in constant pain. I have bone spurs on my hip, okay. You heard my shoulder crack when you did this.

**Q:** Sure, sure, right.

[00:17:25]

**A:** Okay? I'm on the upside of 60. Longevity isn't in my-- genetically in my family. So, I don't--

**Q:** Okay, yeah.

**A:** When I get up and my feet hit the floor in the morning, as long as my feet hit the floor, and I'm not behind bars, I'm a happy man.

**Q:** Fair enough. So quality of life sounds like it’s something that is really important. And so things that are part of your health that affect quality of life take some priority? Is that a fair assessment?

[00:17:51]

**A:** Yes. I'm not going to dwell on every little thing that I may not have control over. Because that would drive me crazy, and it’s-- it is something that I watched my sister go through and I said I will not be like that.

**Q:** She would dwell on health issues for--?

[00:18:21]

**A:** Anything she would dwell on. She has a history of depression and what not, and I think one of those things was she's been to McLean a few times. And I saw how that affected her, or how she would take one issue and just with laser vision just on that issue only and I saw how it affected her. I said I'm not going to do that.

**Q:** Fair enough. Asking it in a little bit of a different way, this is a little bit of an arbitrary question, but if you were to rank your health priorities, where would having a silent stroke rank on that list?

[00:19:03]

**A:** Oh, I think it would be definitely way up there.

**Q:** So it’s up there?

**A:** Oh yeah, definitely.

**Q:** But there are other things that supersede it or other things that surpass it?

[00:19:12]

**A:** Oh no, this is number one.

**Q:** Oh, it is? Okay.

**A:** This is number one. I don't take it lightly, but I can't let it live for me type of thing.

**Q:** Sure. So you don't want to obsess about it, but it has a high priority. Is that a--

[00:19:27]

**A:** Yeah, like anything, once I'm made aware of something that is bad for me, I do whatever I can to avoid it and get it better.

**Q:** Okay, fair enough. Thinking a little bit about having a diagnosis or a medical problem can lead to other things for your health, now that you've been told that you had a silent stroke, do you think this might cause other bad things for your health? And if so, what sort of things?

[00:20:06]

**A:** Well, did I definitely have a silent stroke? That's the question I have. So I'm not aware of what it is, but it’s-- it is what it is.

**Q:** Okay. We can talk a bit more about it afterwards because I know it’s a bit of a challenging concept and it’s hard to counsel about it, too, for a lot of physicians. But assuming that is the case, that we're 100 percent sure that you had a silent stroke, so stroke without symptoms, are there particular problems that you think might follow that or that you would want to avoid just based on your--

[00:21:10]

**A:** Yeah, keeling over by being silent from another stroke and that that would be the last one.

**Q:** So having a stroke with symptoms you think is something that might follow a silent stroke?

**A:** I mean, I've torn up my ankles many times, my knees and what not. So what it is, that tells me through-- what can you avoid so you don't do this again? I don't know what I can do to avoid another stroke because it seems I'm lucky enough to still be here. I mean, some people might have one stroke and they're gone.

**Q:** We do things, like we try to improve our health or enact changes or plans in order to reduce the chances of something bad happening, future health problems. In your opinion, having a silent stroke, what do you think are the chances of having future health problems as a result of that? So for example, what do you think in your mind is the chance of having a stroke with symptoms after having a silent stroke? So is it a very high chance, sort of intermediate, a low chance? Really not at all?

[00:22:28]

**A:** A concern?

**Q:** More just the chances of something happening, the probability, the likelihood of something bad occurring? Do you think you're fighting against a very-- something that's definitely going to happen or something that--

[00:22:42]

**A:** Is it inevitable? I don't believe so. It will make me aware of the signs of the stroke, what's, you know--

**Q:** Okay, so you want to be able to learn about the stroke symptoms or the signs that might occur that can clue you into something happening?

[00:23:03]

**A:** I mean, is it affecting another area of my health that I would have no idea that they were intertwined? No, this is-- it’s eye opening, but I'm not going to be concerned about it. I can't do that.

**Q:** Okay.

[00:23:29]

**A:** I can't walk on eggshells thinking it’s going to happen. Because the stress of that alone might-- could lead to--

**Q:** Sure, sure. At this point in time, do you think that this is connected to your other health issues? And if so, which ones?

[00:23:49]

**A:** No, my other issues, I have no idea how they’d be related to the stroke. I don't know how having a stroke would cause pain in my bladder.

**Q:** Okay, fair enough.

[00:24:07]

**A:** I mean, I don't know how having a stroke had anything to do with my tear in the supraspinatus. I mean, it's just--

**Q:** Sure, okay. How did you describe this to your family and friends, if you did? Did you talk to anybody in your family about--

[00:24:25]

**A:** Yeah, I talked to my wife. I said, “They think I might have had a stroke.”

**Q:** How did she respond to that?

**A:** She said, “Oh, great,” and that was it.

**Q:** A very short conversation?

[00:24:34]

**A:** Very, very short conversation. There was no soul searching or anything. “Oh, you had a stroke? Well, basically, don't have another one.”

**Q:** Okay, fair enough. Just move on, make sure you're doing the right things.

**A:** Just move on.

**Q:** Okay.

[00:24:50]

**A:** I mean, you didn't go out and grab a book, you know, *Strokes for Dummies* or something like that, to see what you could do. It's like okay, well.

**Q:** Okay, fair enough. Have you seen other doctors since seeing me?

[00:25:05]

**A:** No.

**Q:** Okay. I was just going to ask if you had-- how you describe this to other doctors, but it sounds like you haven't had that opportunity?

[00:25:11]

**A:** I had one meeting-- I was in here for gastroenterology, it was a follow-up from something a year or so ago. I didn't mention it to him.

**Q:** Okay, fair enough. You didn't think it was necessary to mention that?

[00:25:26]

**A:** No, I deal with, you know, my primary care, the people she sends me to. I don't walk in and say, “How you doing? Hey, I had a stroke.” [laughter]

**Q:** Sure, fair enough. How would you have wanted this finding to be explained to you? Is there anything that you would have wanted different, or anything, additional information, that you would have liked?

[00:25:53]

**A:** No. I just wanted to-- a degree of certainty that everybody was on the same page and that yes, this is what it was.

**Q:** Okay. And do you think you will change anything at this time now that you have this diagnosis? You kind of mentioned that this was a wakeup call?

[00:26:17]

**A:** Yeah. I mean, I'll-- you know, I'll research it a bit. You know, if I could get a bit more literature on it. But like I say, one thing I will not do is I will not obsess over it.

**Q:** Sure. That sounds like that's something that's important to you.

**A:** I'm not going to let-- at this point, I'm not going to let this rule how I live.

**Q:** How much do you want to make a change as a result of having this diagnosis or hearing about it?

[00:26:47]

**A:** Oh, I'd be willing to do whatever I can, as long as, you know, I know I'm on the right track. I mean, I don't know if it’s-- is it diet related, is it physical health related? I mean, can I stop another stroke from coming by diet and exercise? It’s something I never thought about because I never thought I had one.

**Q:** How urgent is it for you to take action about this? Is it something that you think you need to start making changes now or it can wait for a bit? You want to wait for other things to settle down before taking action? What's your sort of sense of urgency in terms of making a change?

[00:27:40]

**A:** I'll look at things differently. I'll definitely try to keep my composure. I've been having problems with-- I guess you'd call it anger management, stress. So I'll just sit back and say, “You know, you had a stroke, dummy. Don't get pissed off so easy because that's not good for you.” So just those little behavioral things, just let the water roll off your back and just stay even keeled.

**Q:** Fair enough. And we’ll talk more about this, but what sort of things do you want to do besides leveling your sort of stress level and dealing with anger management issues? Are there other things that you want to implement as changes in your life?

[00:28:42]

**A:** No, I think I don't really understand-- I'm having a hard time wrapping my head around what I can do when I really don't know what I can do. I mean, is there a stroke diet? Is there a stroke regimen that you could-- you know, if you do this particular thing, if you swam or if you exercise or if you change behavior, will that reduce the chance of having another one? That's-- I don't-- I don't know that yet. But if I do have that, then I would definitely right off the bat start doing that.

**Q:** So from what I'm hearing you say, it sounds like you feel like there's some uncertainty or lack of clarity in terms of how to approach this or how to take a step forwards?

**A:** Yeah.

**Q:** Okay. Let me-- we're coming close to the end of the interview, but I want to mention a few things to you, and then we’ll talk a little bit more about this after the interview. So, about what the field knows so far, so in stroke neurology, cardiovascular medicine, internal medicine and so on, there's a lot of information about how to prevent strokes before they occur, which we call primary prevention. And then also how to prevent a stroke from happening again once somebody presents with stroke symptoms, whether it’s what we call a transient ischemic attack, which is a precursor to stroke or a stroke that has overt symptoms that might persist for a period of time before resolving, if they do resolve.

So, the idea of silent stroke, or stroke without symptoms, is actually probably somewhere in between that, in that we see evidence of the brain having been injured by the same process, or what we think is the same process, but it's not necessarily clear which set of guidelines to follow, which set of marching orders to take.

And so currently, there are actually no national guidelines or specific research studies that guide physicians in selecting tests or therapies or certain lifestyle modifications for silent stroke specifically. So knowing that, does that make you more concerned about this condition? Does that worry you or does that--?

[00:31:21]

**A:** No, but what it does do, it’s-- if there is information out there that will be helpful to me, I definitely look into it to see what I can do. I know one of the things is my wife keeps telling me all the time, calm down. You're going to wig out. I mean, little-- big things don't bother me. If the room was on fire, I couldn’t care less. You just get low, you head to the exit and leave, okay? Someone does something stupid, a guy-- a kid doesn't-- a woman gets on the train and a young guy doesn't get up to give her the seat, I want to crack him because where’s your manners, you know? There's a woman. You're sitting there with your buds in and everything. You're all about yourself, and give this woman a seat. That drives-- that drives me nuts.

**Q:** Fair enough. And knowing that there aren't specific guidelines or research studies, would that affect how you perceive or trust the advice of your doctors in terms of what they suggest for you to do?

[00:32:37]

**A:** I've been very lucky to have doctors that I've trusted. I can't say that-- oh, I shouldn’t say that. There's one particular guy that I think he’s a quack. No, I put a lot of trust in the doctors’ findings.

**Q:** Okay, so that probably wouldn't necessarily change it too much then?

**A:** No.

**Q:** How important is it to you that the condition be studied more in order to figure out the optimal strategies to approach it?

[00:33:14]

**A:** Well, I really don't want to make this the center of how I'm going to live the rest of my life. I don't want to concentrate on this into-- let’s just say forget about the other stuff. This isn't the big thing to me. Maybe it should be, I don't know. But right now, there are other things that physically I have. I mean, like I say, I'm in pain. That registers with me that something’s got to be done. But the stroke thing? I mean, should it be number one on my priority list? Probably should, but right now it isn't. And I think that's how I deal with it so I don't obsess with it.

**Q:** Okay, fair enough. If studies were done and they found some health benefits, let's say they're relatively modest or relatively small benefits, with either medications or lifestyle changes, do you think you'd be motivated by these studies to, for example, take a new medication like another daily pill or to change your lifestyle, like it’s increasing exercise or modifying the diet?

[00:34:43]

**A:** I would prefer to modify the diet and the exercise without taking another medication. I mean, I have a medicine cabinet full of medications just for me, and I looked at it and I said, “This is what old people have.” And then I'm saying like, well, I am old, but I mean, I look at daily pills, there's like seven of them. I'm saying, “Well, wait a minute, this is--“

**Q:** Out of curiosity, so I'm hearing a couple of different messages from you about priorities and I understand or sort of hear you in saying that you don't want to obsess about this, about the idea of having a silent stroke. But at a different point in the conversation, you also describe that it is number one in terms of the priority list. And so it seems like there's a degree of conflict there in terms of trying to figure out how to approach it?

[00:35:39]

**A:** I look at it from like the other physical problems I have, okay? It's just another one of the problems I have, but it certainly is more important than I walk with a limp because my hip is screwed up. It’s more important than my knees. I can't climb ladders anymore. There's physical activities I can't do, okay? And it’s more important than having-- my pain is being managed, I wish that the Elmiron worked, but it only works for 50 percent of the people, I wasn't one of them. So with all the other things that I deal with on a daily basis, yes this is because this is the one that could, you know, put me in the earth. That's what I mean by--

**Q:** Yeah. So it sounds like, and correct me if I'm wrong, but it sounds like you recognize that there can be a major impact from this issue. And so in that sense, it is a high priority. But at the same time, there are other things that are certainly kind of commanding your attention and are sort of more present because, for example, pain as a symptom. What I'm wondering, is there-- thinking about-- and again, I know you might not want to think about this-- but reflecting on how you're approaching this particular issue, is there some fear there, like fear of thinking about this? Because you've mentioned the experiences of your sister kind of obsessing about a number of different issues. Are you worried that you would become obsessed about this type of thing because of the potential consequences?

[00:37:21]

**A:** Yes.

**Q:** Okay and so you don't want to go down that road?

[00:37:25]

**A:** No, I won't go down that road.

**Q:** You want the sort of simplest approach to dealing with it and then just kind of check that off your list and then kind of one and done?

[00:37:35]

**A:** I don't want this to be the 800 pound gorilla on my back for the rest of my life. I'm aware that it’s there, but forewarned is-- and I've been. So, like I say, if there are measures that I can take without more pills and what not, then I will take them. But I'm not going to say just-- I'm not going to have it be like I have a stroke. I'm not going to go out for pizza because it might lead to a stroke. I couldn't do that.

**Q:** Yeah, okay. Fair enough. No, that's very helpful to hear. So actually, I'm almost done with the interview. I just wanted to see if actually after going through the process of the interview if your thoughts about silent strokes have changed at all? And if so, how?

[00:38:27]

**A:** Yeah, the severity of what could happen. I mean, this-- I don't want to be in a wheelchair. I don't want to-- I want to be able to speak. I don't think I could-- I'd have a very hard time dealing with that. I would, but it’d be hard. But I can't make myself have this be all my life is going to be about. I just can't-- I couldn’t live with every decision, every act, everything I do, concerned about will this cause a stroke, cause a stroke? Like to be aware of some of the things to avoid, but I'm not going to let this drive the car.

**Q:** Sure. Do you have any other questions or additional thoughts that you want to add before I stop the recording?

[00:39:34]

**A:** Well, one of the things that-- and it’s related to this, and other procedures or other procedures I've had with other doctors in different departments, is that the word stroke is very, very serious. And maybe it was the way I saw it, but the way it was told to me was rather kind of abrupt. It was like, “Oh, what are you talking about?” Same, the reaction from my primary care physician, from Dr. M [?] was like, wait a minute.

I mean, when I started with gastroenterology a couple of years ago, the girl-- not the girl, I shouldn't say that-- the doctor, the female who saw me first, I thought I was going to get measured for a body bag. I mean, she was talking about cancer and what not. And I'm saying, “Whoa, you just went to step three. I'm here for step one.” So I think a lot of things is that the way some things are presented to you.

[00:40:58]

When I went to the urologist here, I mentioned something, he says, “Oh, that couldn’t be.” So he just blindly just whatever I thought, “No, no, that can't be it.” I mean, you asked me a question, I told you what's happening and you told me it didn't happen.” So those-- but in this particular instance, here with neurology, I think it's been handled very, very professionally and that's why I have, you know, trust in the people here.

**Q:** I'm glad, I appreciate that.

[00:41:33]

**A:** And I appreciate that Dr. S, with all the testing that he conducted, was-- wasn't vain enough to say, “You don't need to see someone else, I have the answer,” that someone would go to somebody else for a second opinion, maybe even a third opinion needed, that helped. Because I know that some people are just like-- some doctors I've dealt with, they're like the old Irish priests, their way or the highway.

**Q:** Fair enough, I hear you. Well, I'll stop the recording right now.

END OF INTERVIEW

**Participant 11**

**Q:** So, this is the interview with our 11^th^ patient. Can you say hello?

**A:** Hello.

**Q:** Most of these questions are open-ended. I'll ask you a few more specific questions depending on how talkative you feel. It usually takes about 30-40 minutes for the interview. So, the first question is very open-ended, but a question just to have you tell me what do you know about your diagnosis?

[00:00:32]

**A:** I know nothing about my diagnosis. I didn't even know I had the strokes until they let me know that I had two stroke spots on my brain. I guess they were blood spots. I don't know, they were bleeding or there was a little blood. That's how they noticed, I guess. I had an MRI for my aneurysm and they found the stroke spots. I had no idea I had even had a stroke.

**Q:** These were actually strokes were a blood vessel gets blocks, rather than a bleeding.

**A:** Does it get unblocked?

**Q:** They get unblocked, exactly, yes.

[00:01:12]

**A:** So, it's already unblocked?

**Q:** Exactly, yes. So, that has long since passed. But you were mentioning this was done in the setting of having some screening brain MRIs?

**A:** Because I have an aneurysm, yes, 4 millimeter.

**Q:** So, who told you about the strokes initially, was it the neurosurgeons or somebody else?

[00:01:38]

**A:** Yes. Yes, it had to be the neurosurgeons. I went to go see-- well, now I don't even remember now that you asked me, because the neurosurgeons-- my have an aneurysm, it runs in the family. Three of my family members have already passed from aneurysms. I went for an MRI. I was going every six months, and it hasn't changed at all. So, now I'm going every year. It must have been them that let me know, because they're the only ones that do my MRIs.

**Q:** Had you had a problem with the--

**A:** My doctor had referred me to you.

**Q:** Going back a little bit further, have you had a problem with the aneurysms, like did they bleed or did you have headaches or is it just because of your family--

[00:02:30]

**A:** No. Because of my family I decided to have myself checked, because my younger sister, once she had hers I had myself checked and lo and behold, they found one.

**Q:** Do you remember--

**A:** So, I was really glad that I--

**Q:** Had it checked, yes, of course. Do you recall who in the neurosurgery office told you about the strokes and the screening?

**A:** No, I don't, because I see so many-- I mean, I have diabetes, I have hypertension, I have so many things that-- I have liver problems that-- oh yeah, I forget. My memory is not-- that's what goes a lot is my memory.

**Q:** Fair enough. How did he or she present the diagnosis to you? What did they say?

[00:03:18]

**A:** They just let me know that they found two stroke spots. I think they called the blood spots at first. I know they said they found two blood spots on my brain when they did the MRI that consisted of-- they said there were stroke spots and they had referred me to a neurologist, you.

**Q:** Did they ask you any questions or describe anything about them?

[00:03:46]

**A:** Well, I told him, I said, strokes? I never knew. I didn't feel anything or-- and no, they just referred me to you.

**Q:** Did they ask you about any prior symptoms or events, episodes?

**A:** No. I guess they just figured I would talk to you, since you are a specialist.

**Q:** Out of curiosity, do you recall anybody ever using the terms, "incidental" or "unexpected" when first describing the nature of the strokes?

**A:** No. It was unexpected to me, because I die-- I was the one that brought that up. I said, I don't remember having-- because I've had my members who have had strokes so I know. I knew right away when my brother had one he didn't really know, I don't think, because his slurring, his speech was way off. So, I said, I think you had a stroke and you need to go get it checked. But that's what I would have-- if I would have felt something like that, like pains in my arms or slurred speech or something, then I would have thought, I would have said, I think, maybe, I would have went and got checked.

**Q:** Let me ask you a different question. This idea of the strokes being unexpected or being a bit of a surprise, how does that impact you or what sort of--

[00:05:17]

**A:** It's very scary, because I have two younger children, because I had them late in life, 40. So, I have a 12-year-old and a nine-year-old, she just turned nine, and I'm going to be 50. I want to be around for a while. So, with the aneurysm and my other problems, this is scary, because if I had two and didn't even know about it, there are good chances that I'll have another one if I don't change. I'm sure I'm going to have to change my eating habits. So, I've been trying and things have been looking good. Even my blood sugar has gone-- I was at 10, my A1C was at 10, and that's high. And I've already brought it down to 8.5, and that's only within months. So, I'm really trying. I walk all day long, just about every day. Everywhere I go I walk. I eat a lot more vegetables and fruits, because I used to-- I love junk food. So, I've cut out-- ice cream is my biggest thing, so I took out half the fat and no added sugar in my ice cream. So, I just cut out a lot of stuff and referred to fruit and vegetables.

**Q:** Thinking back to that first visit with the neurosurgeons, did they give you any advice on things to do besides seeing a specialist.

**A:** No.

**Q:** Did they seemed concerned about this?

[00:07:06]

**A:** I really have a bad memory, but nothing that would strike me, you know, like none at all really, just having me come see you.

**Q:** I'm going to ask you some questions now as if I don't know the answer, but this is just to get your thoughts on the recording. So, you eventually came to see me as a stroke specialist.

**A:** Yes.

**Q:** Do you remember some of the advice that was given to you at that time in terms of things to do?

[00:07:39]

**A:** Like I said, my memory is bad. I kind of like use my common sense about things. Quit smoking I'm sure was one of them, because I told you that I smoked.

**Q:** I think that is something that we did talk about. Do you recall how concerned I seemed to be about you having said this?

[00:08:03]

**A:** You were concerned. At least you were asking me, I understand that's your job, but I like talking to you. I thought that was good, because I worry myself a lot about this stuff.

**Q:** When I talked to you at that time, did I seem to express any doubt about the diagnosis or--

**A:** Not at all. You wanted to help me. Just like the one prescription that you did give me, I don't know, you must had to have been talking to me, because I've been doing really well since, at least a lot more health wise, since I started seeing you.

**Q:** Which prescription was that?

**A:** I forget what it's called.

**Q:** Is that the cilostazol.

**A:** Cilostazol, that was it. I mean, that brought my blood pressure down. I don't know if it has anything to do with-- I mean, my blood pressure is down, my blood sugar is down and I've lost five pounds. So, just that one medication is really helping me. I suppose it has to be the medication. Yeah, I changed some things. I've always walked, but I just, I'm more health conscious now. I think it's because I'm actually coming to see you. I take whatever you say to heart.

**Q:** Did you feel that way in terms of being health conscious before learning about the strokes or was this a trigger?

[00:09:41]

**A:** No. I think it was a trigger, because I had already had the aneurysm, and this is all going on in my brain. I think it was, even though I didn't know I had the strokes, now that I know, I feel a lot more aware of things. I try to be more aware of things, what I eat and what I do. My smoking has come down a lot. I used to smoke over a pack a day. Now, I'm like a half a pack a day now. I've cut down a lot. To me, that's a lot.

**Q:** What are your concerns about having this diagnosis?

[00:10:28]

**A:** I worry, because I have two younger children that I love to death. They have nobody but me and my sisters and that family, but I worry about them a lot. If something happens to me, they're still young.

**Q:** How bad do you think this is for your health?

**A:** I think it's very-- well, I think it's very bad if I don't stick with the changes that I've made. I don't want to go back to just not thinking about what I eat, not thinking, because I'm afraid that I'll have another stroke. This time I will know and I'll end up in the hospital or even dead, who knows, especially with the aneurysm. Like, I used to worry about my aneurysm, because my sisters, they just passed. The aneurysms just blew. I knew you don't even really have time to get to a hospital.

**Q:** So, you know about aneurysms and bleeding from the experience of your family. I think mentioned your uncle had had a stroke, is that correct, or that your brother?

[00:11:45]

**A:** My father, my brother, yes. My brother and my father had three strokes.

**Q:** Is that how you had learned about stroke as an issue that you learned from their experience?

[00:11:57]

**A:** Yes. Because my father was half-paralyzed and everything, yes.

**Q:** Had anybody ever talked about strokes without symptoms or is that a new idea?

**A:** No. That was something I couldn't even believe it. But at that point I was thinking, well, gees, it could have been that bad. I didn't even know I had them. But who knows. Just like they call things the silent killer, like high blood pressure, you have no knowledge that your blood pressure is high. Yeah, you see spots or you get headaches, but you don't know.

**Q:** Thinking about your other health issues, how does this rank in terms of priority?

**A:** It's top priority, because the aneurysm I get checked every year now. I still worry about it, but strokes, I don't know, it just seems a lot more, like it's a lot easier for me to have a stroke than from my aneurysm to blow, because of my lifestyle.

**Q:** What things about your lifestyle do you think are leading to having strokes?

[00:13:10]

**A:** The smoking is a big one, and just the way I eat, anything, because a lot of things run in my family. So, even no matter how hard I try, just in my head I think maybe things are going to happen, because they're hereditary a lot of stuff. I always think no matter how good I take care of myself, maybe the damage is already done, you know what I mean? Like, I've already had two, so maybe I'm prone to have them more without even knowing, and that could make it a lot worse, who knows. I don't know, that's for sure. I'm not a doctor.

**Q:** So, now that you've been told that you have had a silent stroke, do you think this might cause other bad things for your health? Like do you think there are specific problems?

[00:14:09]

**A:** Yes, I do.

**Q:** What sort of problems do you think it would cause?

**A:** I have no idea, but that worries me that I think about these things a lot. Like, just the way I eat, because I'm not perfect at all.

**Q:** Sure, nobody is.

**A:** And I don't like salads. So, just trying to-- like, I eat a lot of tomato sandwiches now, things like that. I scared to touch bacon. But I love the stuff though. Maybe I keep thinking if I start eating them, you know-- should I even take the chance of eating one bacon sandwich, things like that.

**Q:** I think you said this stroke didn't cause any symptoms, but you're worried about stroke that might have symptoms in the future, ones that--

[00:15:07]

**A:** Yes, and just things that I do in general. Smoking is like, I try to hard, but I just can't quit. It's too hard for me.

**Q:** It is a challenge. It's a long-term challenge, but it's one that I think you're making big progress on.

[00:15:24]

**A:** Well, you're board. I work for the school department, so all summer I don't work, summer vacations, I mean, even the weeks, Christmas, April, May, February.

**Q:** Besides strokes with symptoms, do you think there are other health issues that this might cause?

**A:** I have no idea. I know nothing about it. I'd like to know though.

**Q:** Yes, we can definitely talk more about that.

**A:** Because I do have other issues, health issues.

**Q:** Besides cigarette smoking and diets, do you think it's connected to any other health issues, having these silent strokes?

**A:** I have no idea. I have no idea at all. Like I said, I would like to know though. I'd like to try and do things that-- I'd like to not do things that are prone to having strokes, but I wouldn't know what that was.

**Q:** How did you describe this news to your family and friends?

[00:16:31]

**A:** I just had my sister left that I'm close to. So, I mean, me and her talk all the time and she knows I worry. She knows. We help each other out.

**Q:** Did you talk to her about this? Did you mention this to her?

**A:** Oh yeah, I tell her about all of my doctor's appointments.

**Q:** How did you frame that to her? How did you explain--

[00:17:00]

**A:** Just Sandra, can you believe that I had two strokes. I called her and I said, "Listen, this happened to me." So, she's like, "Well, you have to take it easy." We just care about each other, because half the family is already just gone. My mother just passed away two years ago. I mean, my brother and sister and father they all passed away within one year, but that was 10, 11 years ago. So, we had my mother and she just passed away.

**Q:** How did you describe this to your other doctors?

**A:** I just let them know.

**Q:** Did they have any questions for you?

**A:** Surprisingly, not really, no. My PCP, I don't know, I don't really get along-- not that I don't get along with her, I just don't-- we're not social. I mean, I'm closer to my diabetic nurse than I am my PCP.

**Q:** I see, in terms of the rapport?

**A:** Yes.

**Q:** Thinking back on the experience of learning about this, is there any way that you would want this finding to be explained to you differently?

[00:18:27]

**A:** No, it's fine just the way-- even though they just came out and just told me, they didn't ask me any questions or anything. I mean, I would have liked to have known some things. You get nervous when you hear things like that. And they don't ask questions. They don't give me any medicine. So, you're like-- it sounds serious, I had two strokes. Are you going to give me any medicine to start me doing something? So, that kind of worried me. And then that makes me thing, maybe it ain't that bad. If my doctors don't really ask me, how bad can it be? But I know just through common sense, I know that it's bad. Especially where I have an aneurysm and I'm always stressed out because I'm always thinking about these things.

[00:19:30]

And I know stress is very bad too. So, I always think I'm in a bad situation.

**Q:** You alluded to this before, but do you think you'll change anything at this time now that you know that you've had a silent stroke?

**A:** Well, you see, the things is I really don't know what's changed. I feel like I'm doing the right things, trying to do anyway, so what more can I do? Except the stress, the stress is what I need to try and relax. Always thinking about this stuff stresses me out.

**Q:** Do you think there is any uncertainty or lack of clarity about how to deal with this or how to reduce your risk?

[00:20:16]

**A:** Well, yes, lack of clarity and-- yes, I do. Because like I said, I'm no doctor and I know nothing about these things.

**Q:** Where do you go to find out more information?

[00:20:30]

**A:** Well, the library, most likely.

**Q:** How urgent do you think it is for you to take some action or make changes?

**A:** I think it's very urgent, where my health isn't its greatest.

**Q:** What kind of things would you want to do?

**A:** As in per se, what?

**Q:** Let me give you some hypotheticals. Let's say there are many potential options, all of which are helpful. Maybe it's taking medication or exercising more or modifying the diet, cutting down on cigarette smoking--

**A:** Modifying the diet, because I try to lose weight-- like, I lost five pounds, but that was because I started doing a lot more extra work. But I feel like I'm heavy. I'm trying to lose-- it's my gut. Why is my gut so big, I'm always thinking? I don't eat a lot anyway. So, that's why I assume I'll be going for an MRI on my stomach, because I have some cirrhosis damage in my liver. I'm just trying to-- and I told the doctor I want to know why I can't lose this gut.

**Q:** She is looking into that?

[00:22:02]

**A:** Because I do a lot of walking. I have really strong legs, very muscular. Why can't I lose-- what is with my belly being so big?

**Q:** Let me tell you a few things. Just to kind of let you know a little bit about where the field is so far, and then actually, after the interview I am happy to talk more about this and give you some advice. One issue that we have is that there are strokes that have symptoms and for strokes with symptoms there are two ways that the medical field approaches this. One is that primary care doctors try to help out everybody to identify issues that can lead to stroke, like high blood pressure, high cholesterol, diabetes, cigarette smoking, having abnormal heart rhythms, and then they try to initiate changes, whether it's lifestyle changes or medications that will prevent stroke before it happens.

Then there are specialists like me, neurologists, who after somebody has had a stroke will go through some testing and investigations and also figure out why the stroke occurred so that we can prevent another one from happening again. So, that goal is preventing a stroke from recurring, from happening a second time. And those two strategies are actually a little bit different. They're called primary prevention and secondary prevention. There has been a lot of studies. So, there are actually a lot of national guidelines in terms of what to do. Interestingly, for silent strokes, for strokes without symptoms, there are not actually any guidelines for them. There is not a consensus in terms of how physicians should approach this. Whether they should prescribe a medication or give lifestyle advice. That actually isn't entirely clear yet. There are also no specific clinical trials. I look at people with silent strokes in the majority of situations and try and figure out how to prevent things afterwards. So, learning about that, the sort of lack of guidelines, the lack of treatment studies, how concerned would you be about that sort of lack of clarity or that lack of information?

[00:24:34]

**A:** I'd be worrying-- what was the question, I'm sorry. You know what it is, all the meds that I take in the morning make me tired. They really make me drowsy. It's a bit of a complicated question and it depends a little bit on how you perceive the medical field and how decisions are made. But oftentimes, doctors will make decisions based on guidelines or based on clinical studies. Sometimes they'll make decisions to guide you based on experience, as well. So, what I'm telling you is actually there are no guidelines and no clinical studies specifically for silent stroke. There are studies for regular symptomatic strokes. And what some people will do is they'll actually extrapolate from that. They'll say, okay, these are the same. Maybe I should advise you to do the same things as if you had had strokes with symptoms. Then there are other people who are like, well, we're not really sure, so maybe I shouldn't be advising those types of things. So, there is not agreement. People disagree in terms of what the right strategy is. My curiosity is in hearing your thoughts about does that bother you that there is no consensus or is that okay?

[00:26:02]

**A:** I think it bothers me because-- I don't even know what I'm going to say now. I'm sorry.

**Q:** That's okay. It's a bit of a complicated question. Does that affect how you perceive or trust advice from doctors if there are no guidelines--

**A:** Yes. Well, guidelines, I don't understand. What is the difference between a stroke with symptoms and a stroke without? I mean, isn't a stroke-- aren't they both--

**Q:** The same?

**A:** Yeah.

**Q:** That's a good question. That's what a lot of people believe?

[00:26:44]

**A:** Yeah, what kind of a stroke is a silent stroke? Is it because I didn't get no face sagging or no face-- or anything, no arm feelings? Like, what is the difference between the strokes?

**Q:** How important do you think it will be for this to be studied more so that there is more clarity?

[00:27:09]

**A:** I think it's very important because you can die. Living with strokes, having half of your body paralyzed is nothing simple, and it's very heart breaking, I'm sure. I always wonder like with my father, how does he feel, knowing he can't-- his face [00:27:38], you know what I mean? When he looks in the mirror what does he say to himself, things like that.

**Q:** Sort of self-perception?

**A:** Yeah.

**Q:** If studies found that there were ways of reducing the risk of having a stroke after a silent stroke, a symptomatic stroke, and let's say this was by lifestyle changes, like increasing exercise or changing diet or maybe by taking medication, do you think that you would-- even if the studies only have a little bit of a benefit, do you think that would be enough to convince you to do those things?

**A:** Yes.

**Q:** Does it make a difference between whether it's a lifestyle change or medication?

[00:28:21]

**A:** No. I just want to know what I can do to make myself not have another stroke, you know what I mean, to better my life or to prolong it.

**Q:** After being asked these questions, has your perception on this idea of silent strokes changed, and if so, how?

[00:28:46]

**A:** No, because I'm still worrying about the same things. Because we have a conversation, I didn't give any information on how do I change things, what do I do. We just talked.

**Q:** Do you have any other questions or additional thoughts before I stop the recording?

**A:** No. I'd like to know what do you think would help me help myself.

END OF INTERVIEW

**Participant 12**

**Q:** So, this is the interview with our 12^th^ patient. Can you say, hello?

**A:** Hello.

**Q:** The first question is just to have you tell me what you know about your diagnosis?

[00:00:14]

**A:** I know that I had a stroke and I have no symptoms, physical symptoms.

**Q:** How did you learn about this?

**A:** I had to have an MRI of my brain and that's when the doctor noticed it on the MRI.

**Q:** Do you remember which doctor it was initially?

**A:** Dr.--

**Q:** It's okay if you don't remember the name.

**A:** I forget his name.

**Q:** Describe the scenario.

[00:00:43]

**A:** I forget his name. It was in Easton. He's the one that told me that he had seen something and sent me over here to Tufts.

**Q:** So, why did he get that brain MRI initially?

**A:** From a pinched nerve. I had seen-- I went in for a pinched nerve on my left side and he-- my primary care doctor had sent me in for a pinched nerve. I had seen one doctor for the pinched nerve and they did some type of test and said that it looked like I had had a stroke and sent me in for the brain scan, and that's where I am today.

**Q:** Tell me a little bit about how those symptoms started?

[00:01:28]

**A:** I just kept having pain going down my left arm. It went from my neck all the way down my arm.

**Q:** Was that happening all the time or was that coming and going?

[00:01:43]

**A:** It was a constant pain all of the time down my arm.

**Q:** How long did you have that before you told somebody about that?

**A:** I had it for a very long time. I just was ignoring the pain and just thought it would go away on its own, until I just said I couldn't take it anymore and decided to have it checked.

**Q:** So, you first went to your primary care doctor?

**A:** Yes.

**Q:** And then your primary care doctor sent you to the neurologist?

**A:** Sent me to-- it wasn't a neurologist, it was like a sports doctor.

**Q:** Like sports medicine kind of specialist?

**A:** Yes.

**Q:** And how it's the doctor in Easton, is that?

[00:02:29]

**A:** Yes. Then I seen a neurologist in Easton, and then he sent me over here.

**Q:** So, the sports doctor is the one who sent you to the neurologist in Easton?

**A:** Yeah, because they're right downstairs from each other.

**Q:** I guess they kind of work together?

**A:** Yes.

**Q:** What convinced them to get a brain MRI, do you know?

[00:02:51]

**A:** Because he wanted to see where-- first it was like for the MRI for my pinched nerve, and then when he seen about the pinched nerve, then when he did the test in the office and he said oh, wait, I think you had a stroke--

**Q:** The test in the office was examining you or is it electrical?

**A:** Just like examining me with my hands out in the air and that's when he said, wait, you have signs of a stroke.

**Q:** So, this was the neurologist in Easton. He examined you in the office and then he got concerned by that?

[00:03:26]

**A:** No. The other guy, the sports medicine doctor did that.

**Q:** So, it was the sports medicine doctor.

**A:** It's a little confusion.

**Q:** So, the sports medicine doctor did an examination in the office and he is the one who thought that there were some signs that might have suggested that you may have had a stroke?

**A:** Yes.

**Q:** Was he the one who ordered the MRI or was it the neurologist?

[00:03:54]

**A:** The neurologist did.

**Q:** So, he sent you to the neurologist because of that concern and the neurologist ended up ordering the MRI to evaluate further?

**A:** Yes. He did an MRI and he did this pin thing on my arm. He did a whole bunch of tests and then he scheduled for the MRI, and then that's when he seen the results of the MRI, and then that's when he sent me here to test.

**Q:** Did the sports medicine doctor or the neurologist describe what they found on your examination that made them think that there might have been a stroke?

**A:** The neurologist just described that they had seen some abnormality on the MRI and signs of a stroke and they needed some better clarification and wanted me to see somebody over here in Tufts, so that way there they could get some clarifications.

**Q:** Was the MRI done before you saw the neurologist or was it after you saw the neurologist?

[00:05:02]

**A:** I want to say after.

**Q:** Out of curiosity, how did-- so, I think you mentioned a little bit about the sports medicine doctor telling you that he thought there might be some signs of stroke on the examination. Did he say anything else at that time?

[00:05:23]

**A:** He had just said that he didn't want to alarm me, he didn't want to get me scared or anything, but he just advised me that he wanted me to go down and see the neurologist downstairs to make an appointment just to be sure to be safe.

**Q:** How did the neurologist talk to you about that? How did he present the issue to you or his thinking about it?

**A:** He had just said that he wanted to run tests to be sure. He didn't want to assume anything also, because that was a sports medicine doctor, so he wanted to be sure. So, he ran tests, he did blood work, he did everything. And then when he did the MRI and he did see that and he did the thing in my arm with the pins in my arm, then that's when he sent me over here, because it did show.

**Q:** The pins in your arm, do you mean testing with a little sharp pin or do you mean testing with electrical stimulation?

[00:06:17]

**A:** He did the electrical thing in my arm. And then he did--

**Q:** The EMG?

**A:** Yeah.

**Q:** Then also with the pins?

**A:** Yeah, with the pins.

**Q:** So, he did those studies. He did the study in the office and also the MRI. Did he call you afterwards to tell you about the MRI?

[00:06:40]

**A:** No. I had to go into the office to see him.

**Q:** So, you saw him the second time?

**A:** Yeah. I seen him three times, and then the third time is when he said, I'm sending you into Tufts and then that way there they can further evaluate you and let you know-- they can do their studies, because that's when they wanted me to see Dr. R(?).

**Q:** And did he say-- when he was describing all of the information in front of him at that time, besides saying to go to Tufts, did he say anything about the stroke itself, like whether he was certain it was a stroke or not certain or anything about the scenario?

**A:** He wasn't sure, because it just showed like the signs of it. It looked as if there was a stroke. He wasn't too sure and he wanted a second opinion at the same time.

**Q:** He wasn't sure if it was connected to your symptoms or he wasn't sure that it was a stroke?

[00:07:36]

**A:** He wasn't quite sure it was a stroke, so he wanted a separate opinion.

**Q:** Did either of them use the terms, "incidental" or "unexpected" to describe finding that on the MRI?

**A:** No.

**Q:** Thinking back on everything right now, the initial symptoms that you've had with the pain in your left arm, do you think that it has any connection to what they found on the scan?

[00:08:13]

**A:** Yeah, kind of probably, because at that time I was having the pain in my arm, and it's not as bad as it was, like with the tingling and the pain that I was having, because I don't have that pain in my arm anymore the way that it was. So, I believe it probably does have something to do with what was found.

**Q:** So, after seeing those two doctors, you came to see Dr. R. How did she frame things for you? What did she tell you about her thoughts?

**A:** After doing her studies and everything she had done, she had confirmed that it was a stroke that I had and she had-- did I do another MRI, I can't remember.

**Q:** I think at some point and time you had a second MRI.

**A:** I think I did, yeah, because she wanted to be sure. But she confirmed everything that I did. She just didn't know when I had it, because I have no physical-- it doesn't show physically that I had a stroke.

**Q:** Then did she recommend anything in particular or did she emphasize anything?

[00:09:32]

**A:** She had just said-- she wanted me to watch my cholesterol just in case, just to see if that might have been. And then she had me do a whole bunch of testing just to see, because she wanted to try to find out exactly what could have caused the stroke. She was trying to find the bottom line of why I had the stroke. And then that's when we started doing the testing on my heart and found the PFO.

**Q:** So, the test to the heart was an echocardiogram, is that right?

**A:** Yes.

**Q:** The heart ultrasound?

**A:** Yes.

**Q:** That was the type that you had to swallow a probe, right, or was it on the surface of the skin?

[00:10:13]

**A:** I did both. I've done both.

**Q:** Do you recall any of the other tests that she ordered?

**A:** I know I've done an MRI, I have done both ultrasounds for the heart, I also did where I had to wear the heart monitor for 30 days. I've done so many tests I just forget.

**Q:** Did she prescribe any medications for you at that time?

[00:10:48]

**A:** She prescribed the Lipitor and she said to continue with the aspirin, and I believe that was it at that point.

**Q:** When she was giving you this advice-- we talked a little bit about the sports medicine doctor and the first neurologist in Easton suggesting a little bit uncertainty about whether or not this is a stroke or what are the right things to do, but when Dr. R was ordering these tests for you and saying that she was certain about this being a stroke, when she is ordering the tests and giving you these recommendations about medications and controlling the cholesterol, how certain did she seem to be in terms of that advice?

[00:11:36]

**A:** She was pretty certain. That's when she also had mentioned that she wanted me to meet with you so that way there she could also get another opinion. And that's when I started meeting with you, and that's when she also was getting the test results back from the ultrasounds. She was also getting the test results back from the heart monitor that I was on. And then I started meeting with you and then that's when we started finding out everything else that was going on with my heart.

**Q:** How concerned did she seem to be when she is conveying this information to you and these recommendations?

**A:** She was pretty concerned. She said that it wasn't like a life threatening. She was pretty-- she didn't think that I was going to have another one like tomorrow or anything like that, but she just was pretty sure that with the Lipitor, was pretty confident that I was going to be okay and things like that.

**Q:** Did the other two doctors, the sports medicine doctor and the first neurologist in Easton, did they seem concerned about this as well, or were they less concerned?

[00:12:58]

**A:** Well, the sports medicine doctor, he was a little concerned, that's why he sent me over to the neurologist. The neurologist, he was concerned-- I could tell he was pretty concerned, but he tried to keep calm at the same time because he didn't want to get me worked up at the same time. That's why he sent me over here to Tufts right away, he sent me here.

**Q:** So, there was something about the way he was talking that you're able to tell, but it wasn't directly in what he was saying in terms of the level of concern?

[00:13:30]

**A:** Yeah, because he wasn't trying to get me worried at the same time.

**Q:** Talking to Dr. R, did she express doubt about anything or uncertainty about anything?

**A:** No.

**Q:** So, you saw, I guess including your primary care doctor, five doctors about this, or six doctors if you include the cardiologist, about this idea of silent stroke. Just in general, if you kind of take a step back, how well do you think doctors understand this issue or understand this diagnosis?

[00:14:17]

**A:** It's kind of hard to say because it just was silent, so it was just like it took everybody by surprise, even myself, my family, everybody, just the way that it occurred. So, nobody actually knew what happened, when it occurred, so it kind of puzzled everybody at the same time. It just was puzzling, to be honest with you. Nobody knew anything, so nobody had any answers at the right time. So, when we would ask questions or when my husband would ask questions, nobody had any answers because nobody actually knew anything, because it was so silent. Nobody knew anything at that point. So, everyone could run the test and try to find out the answers.

**Q:** How did learning about the diagnosis make you feel?

**A:** Kind of scared, scared and nervous at the same time.

**Q:** What were you scared about?

[00:15:21]

**A:** Just scared because you didn't-- like I didn't know that that one had happened. I didn't know if another one was going to happen and when that other one was-- if another one was going to happen again or where I was going to be and who I was going to be around.

**Q:** Thinking ahead, what are your concerns about having this diagnosis, having had a silent stroke?

**A:** Like, what are my concerns about it?

**Q:** Yeah?

**A:** My concerns would be just to, I don't know, just like to probably try to, I don't know-- it's hard because it's silent. Like the stroke that I had was the silent one, so it's kind of hard to have concerns about something that is silent. So, I don't know how to answer that, to be honest with you.

**Q:** I think you had mentioned… it sounds like you were being worried about having another stroke and then you were worried about the concept of who you might be around or if someone is going to be around. Tell me if I am incorrect in reading between the lines, but are you sort of worried about having a stroke with symptoms, is that the idea?

[00:16:55]

**A:** Well, yeah, that's one, and also, if I'm with my kids, for instance, if I'm out with my daughter who is eight years-old, how are her reactions going to be and things like that.

**Q:** If for some reason you behave differently or if your body behaves differently, how much you react to that?

**A:** Yeah.

**Q:** How bad do you think this is for your health?

[00:17:33]

**A:** I think if I continue doing what I'm supposed to do-- I thought before I was doing what I was supposed to do and it just like hit me-- but I think going further knowing that I did have this I think I can be more aware that it could happen again. So, and I think my family and my friends and everybody arounds me knows that it could happen, so they're aware of everything. So, they know what to look out for and that type of things.

**Q:** Thinking about your health in general, how concerned are you about your health after learning about this occurring?

**A:** I'm more concerned, just because it was kind of like a wake-up call. Nobody is promised tomorrow, so knowing that, even when Dr. R said I could actually be sitting here today and then it could happen and I wouldn't be here tomorrow type of thing, like she said, it could happen at any time because it was so silent. So, I try to do what I can, like more better with my health, because I have the kids and my family and things like that.

**Q:** You're pretty healthy and I don't think you have a lot of other health issues, is that correct?

**A:** Right.

**Q:** I was going to ask thinking about other health issues, whether you have them now or whether you're worried about health issues in the future, how does this rank in terms of priority?

[00:19:36]

**A:** Pretty high, just because going through this is like a big thing. It's pretty, pretty big, just because it could have taken my life, not knowing, because I am young and nobody expected this. I didn't have no symptoms of it. Just with the kids being around and nobody knowing what could have happened. If it did happen, nobody knew what it was, so they could have just been like, oh, what's going on with Jen.

**Q:** I apologize that this is upsetting. It can be scary. The next question is a little bit kind of along those lines too, and I apologize if this is upsetting. I want you to sort of imagine, actually think a little bit about what sort of health problems you might be worried about a stroke causing. For example, this stroke didn't cause you any, as far as we can tell, any obvious symptoms or major disability or anything like that. So, in terms of what you're afraid of and what worries you, what sort of problems do you think another stroke could cause or that this stroke might cause?

[00:21:15]

**A:** Probably like a disability, like not being able to go to the park to my kids, not being able to-- even like not being able to move, not being able to talk or communicate, anything along that lines, or even like sometimes not being able to-- just having to lay in a bed or that type of thing. I want to be able to still do the things I do now with my kids, with my family and that type of stuff.

**Q:** In your opinion, having talked to several doctors about stroke and the cause of your stroke, the PFO, what do you think are the chances that you might have future health problems because of having this silent stroke?

[00:22:19]

**A:** Hopefully, everything going forward is worked out good. But I'm not sure, I don't know, because I thought I was a pretty healthy person before all of this, except for just being anemic, but I've always been that. I never really had any health issues, so I thought I was pretty healthy, besides the little bit of things. So, at this point, I don't know.

**Q:** You mentioned describing this to your family. How did you describe this news to your family?

**A:** Mostly, to be honest with you, I actually couldn't, because I just was like, I didn't know how to tell them. My husband actually told most of my family and my friends and stuff, and then my father, I told my father. But my husband did most of the talking and told everybody, so I really didn't say much to people.

**Q:** Do you know how he framed it or how he described it to others?

**A:** No, not really. He said most of it, because he tried to be strong for me. He tried not to get me upset, just because when we spoke with Dr. R she had mentioned also to try to not get me-- like try to be calm, not to be stressed, like try to-- not anything to cause another stroke, because at that point nobody was sure what caused the stroke, if it was stress-related, what exactly it was. So, I had to be calm, non-stressed, so he didn't say anything. He tried to be relaxed around me and tried not to get me upset. So, he tried to be the strong one at that time.

**Q:** When you talked to your father about this, how did he respond to that?

[00:24:34]

**A:** He was upset, but he didn't show-- at that point, he was a strong person for me also. I could tell in his voice that he wanted to break down, but he didn't. He was the strong person. He probably broke down afterwards. But he stayed on the phone, because my father lives in Florida, so he's not here. So, basically, he stayed on the phone with me, let me let everything out, talk with me, consoled me and just told me everything was going to be okay. If anything, he would be here. Even when I told him everything, like even when I told him I had to have the surgery on my heart, everything, he said he was going to be here for all of that, everything. But I'm sure he probably had his moments when he wasn't talking with me. But he was also there for me to be strong also.

**Q:** I'm glad that you are able to talk to him about that. It sounds like your family has been very supportive about this, which is great.

[00:25:52]

**A:** Yes, they have.

**Q:** I don't want you to have to recall the whole conversation with your father, but do you recall a particular way in which you described it to me or particular words that you used to describe?

**A:** Because after every appointment-- because I had told him everything that was going on. I've always talked with him and told him after every appointment. He always said, okay, I'm going to call you or you call me. When I had gotten the news he had just said, okay, make sure you call me or I'll call you. So, I spoke with him and I explained to him and I explained to him that the doctor had seen that I had had a stroke. He was quiet for a few minutes and he said, oh, they did? And I said, yeah. And I could hear in his voice it was kind of like--

**Q:** Wavering a bit?

[00:26:50]

**A:** Yeah. And he just got quiet and he got-- just stayed quiet for a few minutes, and the he had said, so, what's going to happen? What are they going to do? And he was asking a lot of questions and he was asking questions that I didn't have the answers to. And I just said told him that they were going to run tests and things like that and I explained to him what Dr. R had explained to us. Then I just started getting upset and everything and then that's it. At that time, he just told me to relax because he didn't want me to get upset and he wanted me to stay calm and things like that and not be upset around the kids and things like that. That was basically, our conversation.

**Q:** What were the questions that he asked that you didn't have the answer to at that time?

[00:27:44]

**A:** Just basically, what it was caused from, when did it happen, what do they think it was caused from, what else are they going to be doing about it, how long-- when will they have answers, things like that. This was at the very beginning when everything was just--

**Q:** Yes, when you first got the news. Since going to these evaluations and then eventually coming to see me, have you had to describe this to any of your other doctors?

**A:** No.

**Q:** Thinking back on it, would you have wanted things to be explained to you, sort of conveyed to you differently?

[00:28:33]

**A:** No. I think the way that everything went was good. Just because at that point when it all started nobody-- like I said before, nobody had answers because it was a silent one and nobody knew why, nobody knew how, nobody knew when. So, I think everybody handled it good. Everybody was confused at the time. Nobody knew anything, including myself, my family and the doctors obviously, nobody knew anything. So, I think we were all trying to work together to try to figure out what was going on. So, I think everything worked well.

**Q:** The last few questions I'm going to ask you about have to do with making changes and doing things for one's health. I think your story has some differences to some other people that I've talked to in that you had a cause of your stroke identified. We found that you had patent foramen ovale and then you opted to have it closed surgically. If you don't mind, I might just ask you a little bit about that. So, I think both Dr. R and I kind of talked a little bit about that option and how they're different options, and that there is no one right or wrong answer, but a lot of it depends on personal preferences. I'm asking you this question as if I don't know the answer. But just to kind of get you to sort of describe it a little bit for the recording, what encouraged you and inspired you to make the choice that you made?

[00:30:22]

**A:** To have the surgery?

**Q:** Yes.

**A:** One is because I was nervous because when I spoke with Dr. R and when IO spoke with you with having the first stroke already with the blood clot that had already went up and already having another one. So, that was one of the decisions that helped me to make that decision of doing it. The second thing is because with already having you and Dr. R saying, yes I needed to have this done, it was two doctors saying yes to go forward to have this done to prevent another stroke from occurring. So, that's what encouraged me more to go ahead and do it. Another thing is because if in the future things were to happen, maybe my heart later on in the future wouldn't be as strong to have this done and then something else could have happened later on. So, I wanted to go and have it done now so that way there I would be good for later on in the future.

**Q:** So, while you're healthy?

**A:** Yes.

**Q:** Are there other things besides that particular treatment that you'd like to make changes to now that you have this diagnosis?

[00:31:54]

**A:** No, I don't think so.

**Q:** Thinking back on talking to different people, whether it's doctors or family, do you think there is an uncertainty or lack of clarity about how to deal with the silent stroke?

[00:32:12]

**A:** No.

**Q:** We're pretty much at the end. After having talked a little bit about this in the context of the interview, has your perception of silent strokes changed at all?

**A:** No. It's scary, to be honest with you. It is scary not knowing when you're going to have one, if I'm every going to have another one again. Knowing that I had this silent one, and like I said, not knowing if I'm going to have one again, and if I do have another one, is that one going to show physical signs next time? And if I'm going to be around or who I'm going to be around if I do have another one. That's the scary part.

**Q:** We can definitely talk about that after the interview when we debrief. I will mention actually just a few things. It's the issue of silent strokes or strokes without symptoms is actually one where we're still trying to figure out the best ways of approaching this. I think for you your situation is a little bit different in that the way in which you sought care was such that you did end up seeing neurologists, as well as many stroke specialists and we advised you in a way that guided you towards having testing done and the treatment to prevent stroke. But there is actually some uncertainty amongst different doctors in terms of how to approach this. I think a lot of neurologists tend to think that well, if a stroke occurs, this is the brain and we have to protect the brain, so it's worth investigating it more and looking into ways to prevent additional ones from occurring. Because that is one of the things that we do know and that we're certain about that.

When somebody has had a silent stroke, there is an increased risk of having a stroke with symptoms. So, at least some proportion of doctors believes that the right thing to do is to help you uncover what the cost was and help you figure out other things that you can do or have done that you can do or have time to reduce the risk of having a stroke with symptoms. So, that's definitely something that we can talk about. But it is something that we do know that people are more at risk in the future. One thing that might be surprising is that it's actually really common, so for people who are above age 50, which is not their age group, but people above age 50, it's about 1 in 5 people has a silent stroke, which is about 10 times as common as people who have a stroke with symptoms. So, that's for a lot of people kind of startling number, because it just indicates it's a pretty common issue and one we're still figuring out the best ways of approaching that, because primary doctors when they address this they try to address how to prevent the stroke from happening in the first place, and then for neurologists we deal with it after it's already happened and those recommendations are a little bit different.

Hearing about that aspect of that, do you have any questions about that or anything that it would make you think about or wonder about?

[00:35:54]

**A:** No.

**Q:** Why don't we stop the recording right here.

END OF INTERVIEW
